# Analysis of the *Legionella longbeachae* Genome and Transcriptome Uncovers Unique Strategies to Cause Legionnaires' Disease

**DOI:** 10.1371/journal.pgen.1000851

**Published:** 2010-02-19

**Authors:** Christel Cazalet, Laura Gomez-Valero, Christophe Rusniok, Mariella Lomma, Delphine Dervins-Ravault, Hayley J. Newton, Fiona M. Sansom, Sophie Jarraud, Nora Zidane, Laurence Ma, Christiane Bouchier, Jerôme Etienne, Elizabeth L. Hartland, Carmen Buchrieser

**Affiliations:** 1 Institut Pasteur, Biologie des Bactéries Intracellulaires, CNRS URA 2171, Paris, France; 2 Department of Microbiology and Immunology, University of Melbourne, Victoria, Australia; 3 Centre National de Référence des Legionella, Université de Lyon, INSERM U851, Faculté de Médecine, IFR 128, Lyon, France; 4 Institut Pasteur, Plate-forme Génomique, Pasteur Génopole Ile de France, Paris, France; Université Paris Descartes, INSERM U571, France

## Abstract

*Legionella pneumophila* and *L. longbeachae* are two species of a large genus of bacteria that are ubiquitous in nature. *L. pneumophila* is mainly found in natural and artificial water circuits while *L. longbeachae* is mainly present in soil. Under the appropriate conditions both species are human pathogens, capable of causing a severe form of pneumonia termed Legionnaires' disease. Here we report the sequencing and analysis of four *L. longbeachae* genomes, one complete genome sequence of *L. longbeachae* strain NSW150 serogroup (Sg) 1, and three draft genome sequences another belonging to Sg1 and two to Sg2. The genome organization and gene content of the four *L. longbeachae* genomes are highly conserved, indicating strong pressure for niche adaptation. Analysis and comparison of *L. longbeachae* strain NSW150 with *L. pneumophila* revealed common but also unexpected features specific to this pathogen. The interaction with host cells shows distinct features from *L. pneumophila*, as *L. longbeachae* possesses a unique repertoire of putative Dot/Icm type IV secretion system substrates, eukaryotic-like and eukaryotic domain proteins, and encodes additional secretion systems. However, analysis of the ability of a *dotA* mutant of *L. longbeachae* NSW150 to replicate in the *Acanthamoeba castellanii* and in a mouse lung infection model showed that the Dot/Icm type IV secretion system is also essential for the virulence of *L. longbeachae*. In contrast to *L. pneumophila*, *L. longbeachae* does not encode flagella, thereby providing a possible explanation for differences in mouse susceptibility to infection between the two pathogens. Furthermore, transcriptome analysis revealed that *L. longbeachae* has a less pronounced biphasic life cycle as compared to *L. pneumophila*, and genome analysis and electron microscopy suggested that *L. longbeachae* is encapsulated. These species-specific differences may account for the different environmental niches and disease epidemiology of these two *Legionella* species.

## Author Summary

*Legionella longbeachae*, found in potting soil, and *L. pneumophila*, present in aquatic environments, are opportunistic human pathogens that cause Legionnaires' disease, a severe and often fatal pneumonia. The analysis and comparison of the genome sequences of four *L. longbeachae* genomes together with the study of its gene expression program and virulence pattern in different infection models provides important new insight on the organism's lifestyle and virulence strategies. *L. longbeachae* harbors a unique repertoire of secreted substrates, many of which encode eukaryotic like domains that may help the pathogen to subvert host functions and cause disease. Curiously, *L. longbeachae* may also be able to interact with plants. Several proteins present mainly in plants and phytopathogenic bacteria and several enzymes that might confer the ability to degrade plant material were identified in its genome. Interestingly, *L. longbeachae* encodes a chemotaxis system but no flagella, in contrast *L. pneumophila* encodes flagella but no chemotaxis system. It will be an interesting aspect of future research to understand these peculiarities. Finally, the genome sequence and analysis reported here will aid in understanding how *L. longbeachae* causes disease and will open new possibilities to develop tools for rapid identification and risk prediction of *L. longbeachae* infection.

## Introduction

*Legionella longbeachae* is one species of the family *Legionellaceae* that causes legionellosis, an atypical pneumonia that can be fatal if not promptly treated. While *Legionella pneumophila* is the leading cause of legionellosis in the USA and Europe, and is associated with around 91% of the cases worldwide, *L. longbeachae* is responsible for approximately 30% of legionellosis cases in Australia and New Zealand and nearly 50% in South Australia [1] and Thailand [2]. Two serogroups (Sg) are distinguished within *L. longbeachae* but most of the human cases of legionellosis are due to Sg1 strains [3],[4]. Interestingly, unlike *L. pneumophila*, which inhabits aquatic environments, *L. longbeachae* is found predominantly in potting soil and is transmitted by inhalation of dust from contaminated soils [4],[5].

Little is known about the biology and the genetic basis of virulence of *L. longbeachae* but a few studies suggest considerable differences with respect to *L. pneumophila.* In contrast, the intracellular life cycle of *L. pneumophila* is well characterized (for recent reviews see [6]–[8]). *L. pneumophila* replicates within alveolar macrophages inside a unique phagosome that excludes both early and late endosomal markers, resists fusion with lysosomes and recruits endoplasmic reticulum and mitochondria. Within this protected vacuole *L. pneumophila* replicates and down-regulates the expression of virulence factors. It has been proposed that nutrient limitation then leads to the transition to transmissive phase bacteria that express many virulence-associated traits allowing the release and infection of new host cells [9]. This biphasic life cycle is observed both *in vitro* and *in vivo* as exponential phase bacteria do not express virulence factors and the bacteria fail to evade the destructive lysosomes and are delivered to the endocytic network and destroyed [9],[10]. The ability of *L. pneumophila* to replicate intracellularly is triggered at the post-exponential phase together with other virulence traits. Less is known about the intracellular life cycle of *L. longbeachae* and its virulence factors. Unlike *L. pneumophila* the ability of *L. longbeachae* to replicate intracellularly is independent of the bacterial growth phase [11]. Phagosome biogenesis is also different. Like *L. pneumophila*, the *L. longbeachae* phagosome is surrounded by endoplasmic reticulum and evades lysosome fusion but in contrast to *L. pneumophila* containing phagosomes the *L. longbeachae* vacuole acquires early and late endosomal markers [12].

Efficient formation of the *L. pneumophila* replication vacuole requires the Dot/Icm type IV secretion system (T4SS) [13]–[16] and probably more than 100 translocated effector proteins that modulate different host cell processes, in particular vesicle trafficking [17]–[19]. While *L. longbeachae* possesses all genes necessary to code a Dot/Icm T4SS [20], it is not known whether it is also essential for virulence and whether *L. pneumophila* and *L. longbeachae* share common effectors.

Another interesting difference between these two species is their ability to colonize the lungs of mice. While only A/J mice are permissive for replication of *L. pneumophila*, A/J, C57BL/6 and BALB/c mice are all permissive for replication of *L. longbeachae* [12],[21]. Resistance of C57BL/6 and BALB/c mice to *L. pneumophila* has been attributed to polymorphisms in Nod-like receptor apoptosis inhibitory protein 5 (*naip5*) allele [22]–[24]. The current model states that *L. pneumophila* replication is restricted due to flagellin dependent caspase-1 activation through Naip5-Ipaf and early macrophage cell death by pyroptosis. Why *L. longbeachae*, in contrast to *L. pneumophila*, is able to replicate in macrophages of all three different mouse strains is still not understood.

In this study we report the complete genome sequencing and analysis of a clinical *L. longbeachae* Sg1 strain isolated in Australia and compare this genome to three *L. longbeachae* draft genome sequences (one Sg1 and two Sg2 strains) and the published genome sequences of four *L. pneumophila* strains [25]–[27]. In addition, we performed transcriptome analysis and virulence studies of a T4SS mutant of *L. longbeachae*. This has allowed us to propose answers for the questions raised above and brings exciting new insight into the varying adaptation to different ecological niches and different intracellular life cycles of *Legionella* species.

## Results/Discussion

### The *L. longbeachae* genomes are highly conserved and are 500 kb larger than those of *L. pneumophila*

**Figure 1:**
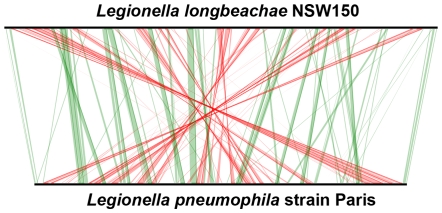
Whole-genome synteny map of *L.longbeachae* strain NSW150 and *L. pneumophila* strain Paris. The linearized chromosomes were aligned and visualized by Lineplot in MAGE. Syntenic relationships comprising at least 8 genes are indicated by green and red lines for genes found on the same strand or on opposite strands, respectively. IS elements (pink), ribosomal operons (blue) and tRNAs (green) are also indicated.

The *L. longbeachae* NSW150 genome consists of a 4,077,332-bp chromosome and a 71,826-bp plasmid with an average GC content of 37.11% and 38.19%, respectively (Table 1). A total of 3512 protein-encoding genes are predicted, 2046 (58.3%) of which have been assigned a putative function (Table S1, Figure S1). The *L. longbeachae* chromosome is about 500 kb larger than that of *L. pneumophila* and has a significantly different organization as seen in the synteny plot in Figure 1 and Figure S2. Moreover only 2290 (65.2%) *L. longbeachae* genes are orthologous to *L. pneumophila* genes, whereas 1222 (34.8%) are *L. longbeachae* specific with respect to *L. pneumophila* Paris, Lens, Philadelphia and Corby (defined by less than 30% amino acid identity over 80% of the length of the smallest protein, s Table S2). It was previously suggested that plasmid-encoded functions such as a two-component system, are important for *L. longbeachae* virulence [28]. Although no similarity was detected between the *L. longbeachae* plasmid here characterized and the 9kb partial plasmid sequence reported of strain *L. longbeachae* A5H5 [28], similar plasmids seem to circulate among different *Legionella* species, as 30 kb of the plasmid of strains Paris, Lens and NSW150, 18 kb of which encode transfer genes (*traI – traA*), encoded ORFs showing high amino acid sequence similarity (Figure S3).

**Table 1:** General features of the completely sequenced *L. pneumophila* and *L. longbeachae* genomes.

	*L. longbeachae*	*L. pneumophila*			
	NSW 150	Paris	Lens	Philadelphia	Corby
Chromosome size (kb)^a^	4077 (71)	3504 (131.8)	3345 (59.8)	3397	3576
G + C content (%)	37.1 (38.2)	38.3 (37.4)	38.4 (38)	38,27	38
G + C content of CDS (%)	37,4	39,1	39,4	38,6	38,6
No. of genes^a^	3660 (75)	3136 (142)	3001 (60)	3002	3259
No. of protein coding genes^a^	3512 (67)	2878 (140)	2878 (60)	2942	3206
Percentage of CDS (%)	84,5	87,9	88	90,2	86,8
Average length of CDS (pb)	1015,2	994,6	935,9	960,7	959,4
No. of 16S/23S/5S	4/4/4	3/3/3	3/3/3	3/3/3	3/3/3
No. transfer RNA	46	44	43	43	43
Plasmids	1	1	1	0	0

**a** Updated annotation; CDS  =  coding sequence; in parenthesis data from plasmids.

With the aim of gaining further information on genome content and diversity of *L. longbeachae* we selected three additional strains, two isolated in the USA one in Australia for genome sequencing and analysis. *L. longbeachae* strain ATCC39642 (Sg1), strain 98072 (Sg2) and strain C-4E7 (Sg2) were deep sequenced using the Illumina technology and then compared to the genome of strain NSW150. We obtained a coverage of 93–96% for each genome with respect to the NSW150 genome (Table 2). The sequences were assembled into 93, 106 and 89 contigs larger than 0.5kbs that were further analyzed regarding gene content and single nucleotide polymorphisms (SNP). High quality SNPs were detected by mapping the Illumina reads on the finished NSW150 genome sequence. This revealed a high conservation in genome size, content, organization and a low SNP number among the four *L. longbeachae* genomes (Table 2). Interestingly, in contrast to *L. pneumophila* where strains of the same Sg may have very different gene content [25],[29], the two strains of *L. longbeachae* each belonging to Sg1 or Sg2, respectively, showed highly conserved genomes. Comparison of the two Sg1 genomes identified 1611 SNPs of which 1426 are located in only seven chromosomal regions mainly encoding putative mobile elements, whereas the remaining 185 SNPs were evenly distributed around the chromosome (Figure S4). In contrast, the SNP number between two strains of different Sg was higher, with about 16 000 SNPs present between Sg1 and Sg2 strains (Table 1, Figure S4). This represents an overall polymorphism of less than 0.4%, which is significantly lower than the polymorphism of about 2% between *L. pneumophila* Sg1 strains Paris and Philadelphia. The low SNP number and relatively homogeneous distribution of the SNPs around the chromosome (Figure S4) suggest recent expansion for the species *L. longbeachae*.

**Table 2:** General features of the *L. longbeachae* draft genomes obtained by new generation sequencing.

	*L. longbeachae*			
	NSW 150	ATCC39462	98072	C-4E7
Chromosome size (Kb)	4077 (71)	4096	4018 (133.8)	3979 (133.8)
No. of 16S/23S/5S	4/4/4	4/4/4	4/4/4	4/4/4
G + C content (%)	37.1 (38.2)	37.0	37.0 (37.8)	37 (37.8)
No. of contigs >0.5-300 kb	complete	64	65	63
N50 contig size*	complete	138 kb	129 kb	134 kb
Percentage of coverage**	100%	96.3	93.4	93.1
Number of SNP with NSW150	–	1611	16 853	16 820
Plasmids	1	0	1	1

*N50 contig size, calculated by ordering all contig sizes and then adding the lengths (starting from the longest contig) until the summed length exceeds 50% of the total length of all contigs (half of all bases reside in a contiguous sequence of the given size or more);

**for SNP detection

### The *dot/icm* type IVB secretion system is highly conserved, and many other secretion systems are present

*L. pneumophila* has a rather exceptional number and wide variety of secretion systems for efficient and rapid delivery of effector molecules into the phagocytic host cell underlining the importance of protein secretion for this pathogen. This also holds true for *L. longbeachae*. We identified the genes coding the Lsp type II secretion machinery, however, 45% of the type II secretion system substrates described for *L. pneumophila* [30],[31] are absent from *L. longbeachae*. Furthermore, the twin arginine translocation system (TAT) and three putative type I secretion systems (T1SS) are present. However, the Lss T1SS might not be functional in *L. longbeachae* as only LssXYZA are conserved (55 to 82% identity with strain Paris) and the two essential components LssB (ABC transporter-ATP binding) and LssD (HlyD family secretion protein) are missing. In contrast, the two additional putative T1SS, encoded by the genes *llo2283*-*llo2288* and *llo0441*-*llo0444* appeared to be functional. Furthermore, two HlyD-like proteins (Llo2901 and Llo0979) localized next to ABC transporters (Llo2900 and Llo0980-Llo0981) were present, but no contiguous outer membrane protein was found. However, these proteins could also be part of T1SS and function together with a genetically unlinked outer membrane component, similar to what is seen for the Hly T1SS of *Escherichia coli* and may thus constitute two additional T1SS. Finally, *L. longbeachae* encodes four type IV secretion systems (T4SS). The Lvh T4ASS of *L. pneumophila* is absent from *L. longbeachae* but we identified three other type-IVA secretion systems. One T4ASS is present on the plasmid and the other two are embedded on putative mobile genomic islands (GI) in the chromosome. *llo1819*-*llo1929* (GI-1) of around 120 kb is bordered by Ser and Arg tRNAs and carries a gene coding for a phage integrase (*llo1819)*. The second cluster (GI-2) of 106 kb spans from the integrase coding gene *llo2859* to *llo2960ab* and is also bordered by a Met tRNA. Most of the proteins encoded on GI-2 are of unknown function. However both islands code for several proteins, which may be dedicated to stress response. On GI-1, Llo1862 and Llo1863*llo1863* are homologous to DNA polymerase IV subunit C and D respectively, involved in the SOS repair pathway. On GI-2 are the OsmC-like protein Llo2923, the putative universal stress proteins Llo2926, Llo2927, Llo2929 and the predicted trancriptional regulator Llo2913 with S24 peptidase domain. Indeed, the S24 peptidase family includes LexA, a transcriptional repressor of SOS response genes to DNA damage. Several transporters were also identified on GI-2: Llo2918 of the MFS superfamily, the Na/H exchange protein Llo2930 and the putative T1SS proteins Llo2900 and Llo2901 discussed above. It possesses in addition a putative restriction/modification system encoded by *llo2865, llo2866* and *llo2867*.

Central to the establishment of the intracellular replicative niche and to *L. pneumophila* virulence is the Dot/Icm type IV secretion system. This T4BSS is also present in *L. longbeachae* and the general organization of the genomic region encoding it is conserved with protein identities of 47 to 92% with respect to that of *L. pneumophila*. This is similar to what has been reported previously for other *Legionella* species [20]. In *L. longbeachae* the *icmR* gene is replaced by the *ligB* gene, however, the encoded proteins have been shown to perform similar functions [32],[33]. Here we found that IcmE/DotG of *L. longbeachae* is 477 amino acids larger than that of *L. pneumophila*. DotG is part of the core transmembrane complex of the secretion system and it is composed of three domains: a transmembrane N-terminal domain, a central region composed of 42 repeats of 10 amino acid and a C-terminal region homologous to VirB10. The central region of DotG from *L. longbeachae* comprises approximately 90 repeats. It will be challenging to understand the possible impact of this modification on the function of the type-IV secretion system.

### The *dot/icm* type IV secretion system of *L. longbeachae* is essential for virulence in *Acanthamoeba castellanii* and in pulmonary mouse infection

**Figure 2:**
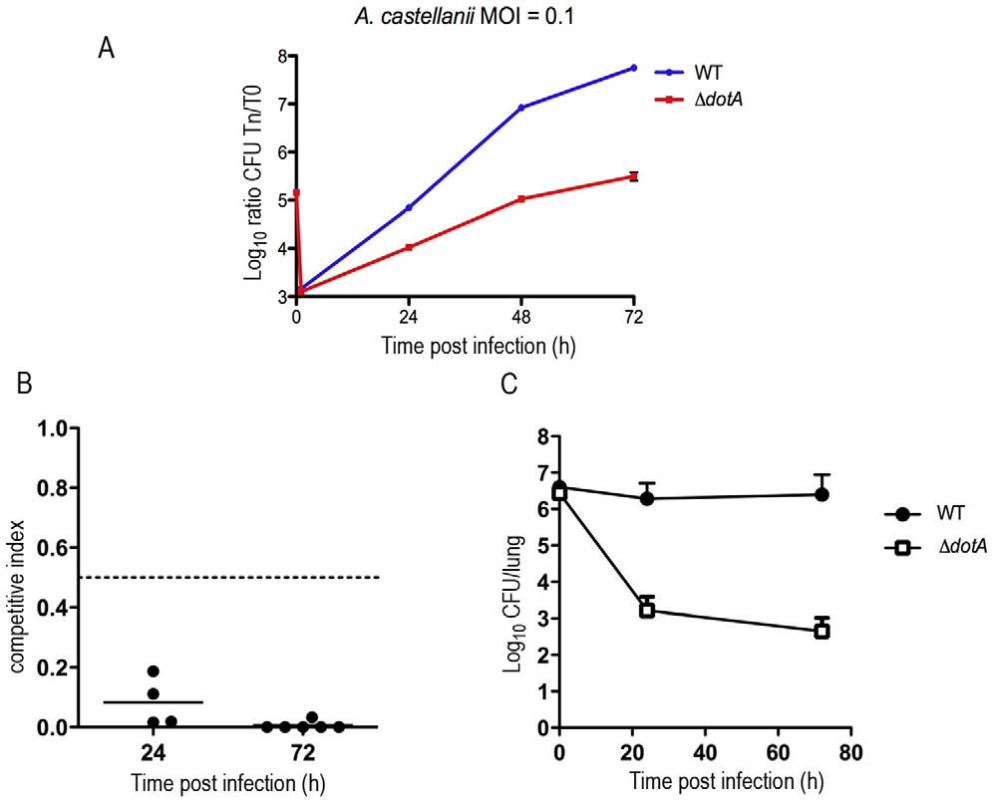
Intracellular growth of the wild-type and the *dotA* mutant strain in mouse and amoeba infection. (A) Intracellular replication of *L. longbeachae* in *Acanthamoeba castellanii*. Blue, wild-type *L. longbeachae* strain NSW150; Red, ***dotA***::Km mutant. Results are expressed as log_10_ CFU. Each time point (in hours, x-axis) represents the mean ± SD of two independent experiments. Infections were performed at 37°C. (B) CI values from mixed infections of A/J mice. Mice were inoculated with approximately 10^6^ CFU of each strain under investigation and were sacrificed at 24 h or 72 h after infection to examine the bacterial content of their lungs. Competition experiment between ***L. longbeachae*** and the ***dotA***::Km mutant representative of 2 independent experiments. (C) Single infections of A/J mice with *L. longbeachae* wt and the ***dotA***::Km mutant strain. Results are expressed as log_10_ CFU. Note: to maintain numbers in the lung *L. longbeachae* must be replicating Non-replicating bacteria are cleared in this infection model over 72 h (*eg.* dotA mutant) [21].

To test whether the Dot/Icm T4SS is essential for virulence of *L. longbeachae* we constructed a deletion mutant in the *L. longbeachae* NSW150 gene *llo0364*, homologous to *dotA* of *L. pneumophila* and tested its ability to replicate compared to the wild type strain in *A. castellanii* and the lungs of A/J mice. We found that *L. longbeachae* NSW150 infects *A. castellanii* in a comparable manner to *L. pneumophila* and that the *dotA* mutant was strongly attenuated for intracellular growth in *A. castellanii*, similar to what is seen for a *L. pneumophila dotA* mutant (Figure 2A). Recently Gobin and colleagues established an experimental model of intratracheal *L. longbeachae* infection in A/J mice [21]. Here we compared the ability of the *L. longbeachae dotA* mutant to compete with wild type *L. longbeachae* in the lungs of A/J mice. In mixed infections, we observed that the *dotA* mutant was outcompeted by the wild type strain 24 h and 72 h after infection (Figure 2B). The competitive index of the *dotA* mutant was calculated by dividing the ratio of mutant to wild type bacteria after infection with the ratio of mutant to wild type bacteria in the inoculum. A competitive index of less than 0.5 is considered a significant attenuation [34]. The competitive index was less than 0.5 at both time-points indicating rapid loss of the *dotA* mutant following infection. In single infections, the *L. longbeachae dotA* mutant was also dramatically attenuated for replication (Figure 2C). Thus, the Dot/Icm secretion system was essential for the virulence of *L. longbeachae*.

### *L. longbeachae* and *L. pneumophila* encode different sets of secreted Dot/Icm substrates and virulence genes

Despite the high degree of conservation of the Dot/Icm T4SS components between *L. pneumophila* and *L. longbeachae* the Dot/Icm substrates were not highly conserved. Indeed 66% of reported *L. pneumophila* Dot/Icm substrates were absent from *L. longbeachae* (Table 3 and Table S3). Instead, we predicted 51 new putative Dot/Icm substrates specific for *L. longbeachae* that encode eukaryotic-like domains and all but one contained the secretion signal described by Nagai and colleagues [35] and many also the additional criteria defined by Kubori and colleagues [36] (Table 4). Interestingly, the distribution of both, the conserved and the newly identified substrates of *L. longbeachae* among the four sequenced strains was highly conserved (Table 3 and Table 4). Both *L. pneumophila* and *L. longbeachae* replicate within a vacuole that recruits endoplasmic reticulum. Several effector proteins have been shown to contribute to the ability of *L. pneumophila* to manipulate host cell trafficking events resulting in this association. The effector proteins SidJ, RalF, VipA, VipF, SidC, YlfA and LepB which contribute to trafficking or recruitment and retention of vesicles to *L. pneumophila* vacuoles were conserved in *L. longbeachae*, but VipD, SidM/DrrA and LidA which interfere also with these events are absent from the *L. longbeachae* genome; however VipD and SidM/DrrA are also not present in all the *L. pneumophila* genomes sequenced.

**Table 3:** Distribution of selected Dot/Icm substrates of *L. pneumophila* in the *L. longbeachae* genomes.

*L. pneumophila*				*L. longbeachae*				Name	Description
Phila-1	Paris	Lens	Corby	NSW150	A	B	C	**	
*lpg0012*	*lpp0012*	*lpl0012*	*lpc0013*	*llo0432*	+	+	+	*cegC1*	Ankyrin repeat
*lpg0038*	*lpp0037*	*lpl0038*	*lpc0039*	*–*	–	–	–	*ankQ/legA10*	Ankyrin repeat
*lpg0103*	*lpp0117*	*lpl0103*	*lpc0122*	*llo3312*	+	+	+	*vipF*	GNAT family
*lpg0171*	*lpp0233*	*lpl0234*	*–*	*–*	–	–	–	*legU1*	F-box motif
*lpg0234*	*lpp0304*	*lpl0288*	*lpc0309*	*llo0425*	+	+	+	*sidE/laiD*	Unknown
*lpg0257*	*lpp0327*	*lpl0310*	*lpc0334*	*llo2362*	+	+	+	*sdeA*	Multidrug resistance protein
*lpg0276*	*lpp0350*	*lpl0328*	*lpc0353*	*llo0327*	+	+	+	*legG2*	Ras guanine nucleotide exchange
*lpg0376*	*lpp0443*	*lpl0419*	*lpc2967*	*–*	–	–	–	*sdhA*	GRIP, coiled-coil
*lpg0390*	*lpp0457*	*lpl0433*	*lpc2954*	*llo2824*	+	+	+	*vipA*	Unknown
*lpg0402*	*–*	*–*	*–*	*–*	–	–	–	*ankY/legA9*	Ankyrin, STPK
*lpg0403*	*lpp0469*	*lpl0445*	*lpc2941*	*–*	–	–	–	*ankG/ankZ/ legA7*	Ankyrin
*lpg0436*	*lpp0503*	*lpl0479*	*lpc2906*	*–*	–	–	–	*ankJ/legA11*	Ankyrin
*lpg0483*	*lpp0547*	*lpl0523*	*lpc2861*	*llo2705*	+	+	+	*ankC/legA12*	Ankyrin
*lpg0621*	*lpp0675*	*lpl0658*	*lpc2673*	*–*	–	–	–	*sidA*	Unknown
*lpg0642*	*lpp0696*	*lpl0679*	*lpc2651*	*–*	–	–	–	*wipB*	Unknown
*lpg0695*	*lpp0750*	*lpl0732*	*lpc2599*	*–*	–	–	–	*ankN/ankX legA8*	Ankyrin
*lpg0940*	*lpp1002*	*lpl0971*	*lpc2349*	*–*	–	–	–	*lidA*	Unknown
*lpg1227*	*lpp1235*	*lpl1235*	*lpc0696*	*–*	–	–	–	*vpdB*	Acyl transferase/hydrolase
*lpg1328*	*lpp1283*	*lpl1282*	*lpc0743*	*–*	–	–	–	*legT*	Thaumatin domain
*lpg1355*	*lpp1309*	*–*	*–*	*–*	–	–	–	*sidG*	Coiled-coil
*lpg1488*	*lpp1444*	*lpl1540*	*lpc0903*	*–*	–	–	–	*lgt3/legc5*	Coiled-coil
*lpg1588*	*lpp1546*	*lpl1437*	*lpc1013*	*–*	–	–	–	*legC6*	Coiled-coil
*lpg1642*	lpp1612*	*lpl1384*	*lpc1071*	*llo1144*	+	+	+	*sidB*	Rtx toxin, lipase
*lpg1701*	*lpp1666*	*lpl1660*	*lpc1130*	*–*	–	–	–	*ppeA/legC3*	Coiled-coil
*lpg1718*	*lpp1683*	*lpl1682*	*lpc1152*	*–*	–	–	–	*ankI/legAS4*	Ankyrin
*lpg1884*	*lpp1848*	*lpl1845*	*lpc1331*	*–*	–	–	–	*ylfB/legC2*	Coiled-coil
*lpg1950*	*lpp1932*	*lpl1919*	*lpc1423*	*llo1397*	+	+	+	*ralF*	Sec-7 domain
*lpg1953*	*lpp1935*	*lpl1922*	*lpc1426*	*–*	–	–	–	*legC4*	Coiled-coil
*lpg1978*	*lpp1961*	*lpl1955*	*lpc1464*	*–*	–	–	–	*setA*	Putative Glycosyltransferase
*lpg2137*	*lpp2076*	*lpl2066*	*lpc1586*	*–*	–	–	–	*legK2*	STPK
*lpg2144*	*lpp2082*	*lpl2072*	*lpc1593*	*–*	–	–	–	*ankB/legAU13ceg27*	Ankyrin, F-box
*lpg2155*	*lpp2094*	*lpl2083*	*lpc1604*	*llo3096*	+	+	+	*sidJ*	Unknown
*lpg2157*	*lpp2096*	*lpl2085*	*lpc1618*	*–*	–	–	–	*sdeC*	Unknown
*lpg2176*	*lpp2128*	*lpl2102*	*lpc1635*	*–*	–	–	–	*legS2*	Sphingosine-1-phosphate lyase 1
*lpg2222*	*lpp2174*	*lpl2147*	*lpc1689*	*–*	–	–	–	*lpnE*	Sel-1 repeats
*lpg2298*	*lpp2246*	*lpl2217*	*lpc1763*	*llo1707*	+	+	+	*ylfA/legC7*	Coiled-coil
*lpg2300*	*lpp2248*	*lpl2219*	*lpc1765*	*llo0584*	+	+	+	*ankH/legA3/ankW*	Ankyrin, NFkappaB inhibitor
*lpg2322*	*lpp2270*	*lpl2242*	*lpc1789*	*llo0570*	+	+	+	*ankK/legA5*	Ankyrin
*lpg2452*	*lpp2517*	*lpl2370*	*lpc2026*	*–*	–	–	–	*ankF/legA14/ceg31*	Ankyrin
*lpg2456*	*lpp2522*	*lpl2375*	*lpc2020*	*llo0365*	+	+	+	*ankD/legA15*	Ankyrin
*lpg2464*	*–*	*lpl2384*	*–*	*–*	–	–	–	*sidM/drrA*	Unknown
*lpg2465*	*–*	*lpl2385*	*–*	*–*	–	–	–	*sidD*	Unknown
*lpg2490*	*lpp2555*	*lpl2411*	*lpc1987*	*llo0796*	+	+	+	*lepB*	Coiled-coil, Rab1 GAP
*lpg2508*	*lpp2576*	*lpl2430*	*lpc1963*	*–*	–	–	–	*sdjA*	Unknown
*lpg2511*	*lpp2579*	*lpl2433*	*lpc1959*	*llo3098*	+	+	+	*sidC*	PI(4)P binding domain
*lpg2556*	*lpp2626*	*lpl2481*	*lpc1906*	*llo2218*	+	+	+	*legK3*	STPK
*lpg2584*	*lpp2637*	*lpl2507*	*lpc0561*	*–*	–	–	–	*sidF*	Unknown
*lpg2718*	*lpp2775*	*lpl2646*	*lpc0415*	*–*	–	–	–	*wipA*	Unknown
*lpg2793*	*lpp2839*	*lpl2708*	*lpc3079*	*–*	–	–	–	*lepA*	Coiled-coil
*lpg2829*	*lpp2883*	*–*	*–*	*–*	–	–	–	*sidH*	Unknown
*lpg2830*	*lpp2887*	*–*	*–*	*–*	–	–	–	*lubX/legU2*	U-box motif
*lpg2831*	*lpp2888*	*lpl4276*	*–*	*–*	–	–	–	*VipD*	Patatin-like phospholipase
*Lpg2999*	*lpp3071*	*lpl2927*	*lpc3315*	*–*	–	–	–	*legP*	Astacin protease

*pseudogene, lpp1612a et 1612b; A: *L. longbeachae* strain ATCC39462; B: 98072; C: C-4E7.

**Table 4:** Putative new type IV secretion substrates specific for *L. longbeachae.*

NSW150	ATCC39462	98072	c-4E7	Motif	A	B	C
*llo0037*	+	+	+	ankyrin	+	42,86	60,00
*llo0087*	+	+	+	ankyrin	+	57,14	53,33
*llo0115*	+	+	+	ankyrin	+	28,57	53,33
*llo0246*	+	+	+	ankyrin	+	28,57	66,67
*llo0990*	+	---	---	ankyrin	+	28,57	46,67
*llo1043*	+	+	+	ankyrin	+	28,57	46,67
*llo1142*	+	+	+	ankyrin	+	28,57	53,33
*llo1168*	+	+	+	ankyrin	+	28,57	53,33
*llo1371*	+	+	+	ankyrin, coiled-coil	+	28,57	66,67
*llo1395*	+	+	+	ankyrin	+	42,86	53,33
*llo1618*	+	+	+	ankyrin	+	28,57	66,67
*llo1646*	+	+	+	ankyrin	+	28,57	40,00
*llo1651*	+	+	+	ankyrin	+	14,29	60,00
*llo1715*	+	+ *	+ *	ankyrin	+	28,57	40,00
*llo1742*	+	+	+	ankyrin	+	57,14	46,67
*llo1894*	+	+	+	ankyrin	+	28,57	66,67
llo2133*	+	+	+	ankyrin	+	0,00	33,33
*llo2476*	+	+	+	ankyrin	+	14,29	46,67
*llo2668*	+	+	+	ankyrin	+	14,29	46,67
*llo3081*	+	+	+	ankyrin, patatin-like phospholipase	+	28,57	60,00
*llo3093*	+	+	+	ankyrin, STPK	+	0,00	66,67
*llo3343*	+	+	+	ankyrin	+	14,29	33,33
*llo3353*	+	+	+	ankyrin, NUDIX hydrolase	+	28,57	53,33
*llo0114*	+	+	+	LRR	+	14,29	40,00
*llo1314*	+	+	+	LRR	+	0,00	40,00
*llo2165*	+	+	+	LRR	+	42,86	66,67
*llo2494*	+	+	+	LRR	+	28,57	66,67
*llo3116*	---	---	---	LRR	+	57,14	26,67
*llo3118*	---	---	---	LRR	+	28,57	66,67
*llo1139*	+	+	+	STPK	+	14,29	33,33
*llo1681*	+	+	+	STPK	+	42,86	73,33
*llo2132*	+	+	+	STPK, coiled-coil	-	14,29	73,33
*llo2984*	+	+	+	STPK	+	14,29	53,33
*llo3049*	+	+	+	STPK	+	14,29	66,67
*llo1984*	+	+	+	STPK	+	14,29	33,33
*llo1427*	+	+	+	F-Box	+	14,29	66,67
*llo2109*	+	+	+	F-Box	+	28,57	60,00
*llo0448*	+	+	+	U-Box	+	28,57	73,33
*llo1404*	+	+	+	PPR	+	28,57	20,00
*llo2643*	+	+	+	PPR, coiled-coil	+	28,57	46,67
*llo2200*	+	+	+	TTL	+	14,29	53,33
*llo2327*	+	+	+	SH2	+	28,57	73,33
*llo2352*	+	+	+	PAM2	+	42,86	60,00
*llo1196*	+	+	+	Snare	+	0,00	73,33
*llo2381*	+	+	+	Snare	+	42,86	60,00
*llo0793*	+	+	+	Phosphatidylinositol-4-phosphate 5-kinase	+	28,57	66,67
*llo3288*	+	+	+	Ras-related small GTPase domain	+	14,29	60,00
*llo2329*	+	+	+	Ras-related small GTPase, Miro-like domain	+	28,57	60,00
*llo2249*	+	+	+	Miro-like domains	+	57,14	80,00
*llo1716*	+	+	+	Ras-related small GTPase, Miro-like domain	+	28,57	73,33
*llo1892*	+	+	+	Putative Immunoglobulin I-set domain	+	14,29	40,00

(A) Presence of a hydrophobic residue or a proline in positions -3 or -4 according to [35]. (B) Enrichment in amino acids that have small side-chains (alanine, glycine, serine and threonine) at positions -8 to -2 according to [36]. (C) Percentage of Polar aminoacids that are favored at positions −13 to +1 according to [36].

Although *L. pneumophila* also communicates with early and late endosomal vesicle trafficking pathways [37]–[39], a major difference in the phagosome maturation of the two species is that the *L. longbeachae* phagosome acquires early and late endocytic markers. Several proteins identified specifically in the genome of *L. longbeachae* may contribute to these differences. First, *L. longbeachae* encodes a family of Ras-related small GTPases (Llo3288, Llo2329, Llo1716 and Llo2249) (Figure S5), which may also be involved in vesicular trafficking and account for the specificities of the *L. longbeachae* life cycle. Remarkably, Llo3288, Llo2329 and Llo1716 are the first small GTPases of the Rab subfamily described in a prokaryote. *L. pneumophila* is also known to exploit monophosphorylated host phosphoinositides (PI) to anchor the effector proteins SidC, SidM/DrrA, LpnE and LidA to the membrane of the replication vacuole [34], [40]–[44]. *L. longbeachae* may employ an additional strategy to interfere with the host PI as Llo0793 is homologous to a mammalian PI metabolizing enzyme phosphatidylinositol-4-phosphate 5-kinase and it is tempting to speculate that this protein allows direct modulation of the host cell PI levels.

As another strategy to alter host trafficking pathways, *L. pneumophila* is able to target microtubule-dependent vesicular transport. AnkX/AnkN, for example, prevents microtubule-dependent vesicular transport interfering with the fusion of the *L. pneumophila*-containing vacuole with late endosomes [45]. AnkX/AnkN is absent from *L. longbeachae*, however *L. longbeachae* did encode a putative tubulin-tyrosine ligase (TTL) Llo2200, which adds to the 19 bacterial TTL identified to date. TTL catalyzes the ATP-dependent post-translational addition of a tyrosine to the carboxy terminal end of detyrosinated alpha-tubulin. Although the exact physiological function of alpha-tubulin has so far not been established, it has been linked to altered microtubule structure and function [46]. Besides AnkX/AnkN, a large family of ankyrin repeat constitutes *L. pneumophila* Dot/Icm substrates. Interestingly, 23 of the 29 ankyrin proteins identified in the *L. pneumophila* strains are absent from the *L. longbeachae* genome, however *L. longbeachae* encodes 23 specific ankyrin repeat proteins (Table 4).

*L. pneumophila* is also able to interfere with the host ubiquitination pathway. The U-box protein LubX, which possesses *in vitro* ubiquitin ligase activity specific for the eukaryotic Cdc2-like kinase Clk1 [36], is absent from *L. longbeachae*. However, *llo0448* encodes a predicted U-box protein. None of the three *L. pneumophila* F-box proteins, which may also exploit this pathway, are conserved in *L. longbeachae*, but we identified two new putative F-box proteins Llo1427 and Llo2109 (Table 4). Thus, although the specific proteins may not be conserved, the eukaryotic-like protein-protein interaction domains found in *L. pneumophila* are also present in *L. longbeachae.*

*L. longbeachae* also encodes several proteins with eukaryotic domains that are not present in *L. pneumophila*. One is the above-mentioned protein Llo2200 encoding a TTL domain. A second is Llo2327, the first bacterial protein that encodes an Src Homology 2 (SH2) domain. SH2 domains, in eukaryotes, have regulatory functions in various intracellular signaling cascades. Furthermore, *L. longbeachae* encodes two proteins (Llo1404 and Llo2643) with pentatricopeptide repeat (PPR) domains. This family seems to be greatly expanded in plants, where they appear to play essential roles in organellar RNA metabolism [47]–[49]where they appear to play essential roles in RNA/DNA metabolism, where. Only 12 bacterial PPR domain proteins have been identified to date, all encoded by two species, the plant pathogens *Ralstonia solanacearum* and the facultative photosynthetic bacterium *Rhodobacter sphaeroides*.

### *L. longbeachae* encodes putative toxins

Recently, a family of cytotoxic glucosyltransferases produced by *L. pneumophila* (Lgt) and related to the group of clostridial glucosylating cytotoxins has been described [50],[51]. The three studied enzymes Lgt1/2/3 target one host molecule, eEF1A, and have been implicated in inhibition of eukaryotic protein synthesis and target-cell death [52]. *L. longbeachae* encodes two putative specific cytotoxic glucosyltransferases Llo1721 and Llo1578. They share only low homology with the *L. pneumophila* Lgt proteins with 23% protein identity over 62% of the protein length and 36% protein identity over 32% of the length, respectively. However, the DXD motif that is critical for enzymatic activity of clostridial enzymes is conserved suggesting that these enzymes might also be active in *L. longbeachae*. We also identified Llo3231 as another putative specific glucosyltransferase with a DXD motif, distantly related to the *L. pneumophila* SetA protein (23% protein identity over 67% of the protein length). SetA is known to cause delay in vacuolar trafficking [53], however its glucosylating activity remains to be established. In contrast, *L. longbeachae* does not encode a homologue of the *L. pneumophila* structural toxin protein RtxA, however we identified a homolog of the TcaZ toxin (Llo1558) present in the insect pathogen *Photorhabdus luminescens* [54].

### Many metabolic features of the genome of *L. longbeachae* reflect its soil habitat

*L. longbeachae* encodes a variety of proteins probably devoted to the metabolism of compounds present in plant cell walls, going in hand with the fact that that bacterium can be isolated from composted plant material. The main components of the plant cell wall are cellulose, hemicellulose and pectin. Cellulose utilization by microorganisms involves endo-1,4-beta-glucanases, cellobiohydrolases and β-glucosidases, that act synergically to convert cellulose to glucose. Examination of the *L. longbeachae* genome sequence revealed the presence of twelve such cellulolytic enzymes. Five glucanases, four cellobiohydrolases and three β-glucosidases are present. Interestingly, *L. pneumophila* also encodes two putative endo-1,4-beta-glucanases and one putative β-glucosidase but does not encode any cellobiohydrolase.

Within the plant cell wall, the cellulose microfibrils are linked via hemicellulosic tethers to form the cellulose-hemicellulose network, which is embedded in the pectin matrix. To gain access to cellulose in plant material, pectin and hemicellulose hydrolysis is necessary. Interestingly, *L. longbeachae* encodes three pectin lyases (Llo1693, Llo1410, Llo1162). The last two proteins possess a signal peptide and may therefore be secreted. Pectin lyases are virulence factors usually found in phytopathogenic microorganisms that degrade the pectic component of the plant cell wall. In addition to these specific enzymes and similar to *L. pneumophila*, *L. longbeachae* encodes a protein homologous to endo-1,4-beta-xylanase. Endo-1,4-beta-xylanase hydrolyses xylan the most common hemicellulose polymer in the plant kingdom and the second most abundant polysaccharide on earth. So, unlike *L. pneumophila,* which does not possess cellobiohydrolase and pectin lyase, *L. longbeachae* seems to be fully equipped to utilize cellulose as a carbon source (Table 5). Soil bacteria also often hydrolyse chitin by the means of chitinases to use it as a carbon source. Chitin originates mainly from the cell wall of fungi and cuticles of crustaceans or insects. In line with the fact that *L. longbeachae* is isolated from soil, we found two chitinases (Llo0050, Llo1558) that are predicted to be secreted proteins. However, the homologue of ChiA from *L*. *pneumophila* that was shown to be involved in infection of lungs of A/J mice [31] is absent from *L. longbeachae*.

**Table 5:** Predicted *L. longbeachae* enzymes that may be involved in cellulose degradation.

Gene	Annotation	SignalP	Predicted localization (PSORTb^+^)	ATCC39462	98072	c-4E7	Homology with *L. pneumophil*a
*llo2355*	Putative endo-1,4-beta-glucanase	+	Unknown	100%	99%	99%	–
*llo3308*	Putative endo-1,4-beta-glucanase	+	Unknown	100%	99%	99%	*lpp1893*/*lpg1918*/*lpl1882*/*LPC_1372*
*llo3305*	Putative endo-1,4-beta-glucanase	+	Unknown	100%	99%	99%	*lpp0546*/*lpg0482*/*lpl0522*/*LPC_2862*
*llo1381*	Putative endo-1,3(4)-beta-glucanase	+	Unknown	100%	99%	99%	–
*llo0032*	Putative cellobiohydrolase	+	Unknown	100%	99%	99%	–
*llo0965*	Putative cellobiohydrolase	+	Extracellular	100%	98%	98%	–
*llo1892*	Putative cellobiohydrolase	+	Extracellular	100%	99%	99%	–
*llo2999*	Putative cellobiohydrolase	+	Unknown	100%	99%	99%	–
*llo1023*	Putative beta-glucosidase	+	Unknown	100%	99%	99%	*lpp1193*/*lpg1191*/*lpl1199*/*LPC_0658*
*llo0330*	Putative beta-glucosidase	–	Cytoplasmic	100%	99%	99%	–
*llo2462*	Putative beta-glucosidase	–	Cytoplasmic	100%	99%	99%	*lpp0946*/*lpg0885*/*lpl0916*/*LPC_2408*
*llo0816*	Putative endo-1,4-beta-xylanase	+	Unknown	100%	99%	99%	*lpp0767*/*lpg0712*/*lpl0749*/*LPC_2581*
*llo1693*	Putative pectin lyase	–	Unknown	100%	96%*	96%*	–
*llo1410*	Putative pectin lyase	+	Extracellular	100%	99%	99%	–
*llo1162*	Putative pectin lyase	+	Extracellular	100%	99%	99%	–

*: frameshift at the N-terminus

+ PSORTb bacterial protein localization prediction tool (http://www.psort.org/psortb/)

Interestingly, *L. longbeachae* encodes a putative cyanophycin synthase (Llo2537) and therefore may be able to synthesize cyanophycin. Cyanophycin is an amino acid polymer composed of an aspartic acid backbone and arginine side groups. It serves as a storage compound for nitrogen, carbon and energy in many cyanobacteria. *Acinetobacter baylyi* strain ADP1 was the first non-cyanobacterial strain shown to synthesize cyanophycin, a metabolic capacity that is still restricted to only few prokaryotes [55]–[58]. *L. longbeachae* also harbors a putative cyanophycinase (Llo2536) enabling the degradation of cyanophycin to dipeptides and a dipeptidase (Llo2535) necessary to hydrolyze beta-Asp-Arg dipeptides. *L. longbeachae* may thus be able to completely utilize cyanophycin, providing a mechanism for energy supply under substrate-limited conditions.

### Genome and electron microscopy analysis indicates that *L. longbeachae* encodes a capsule

**Figure 3:**
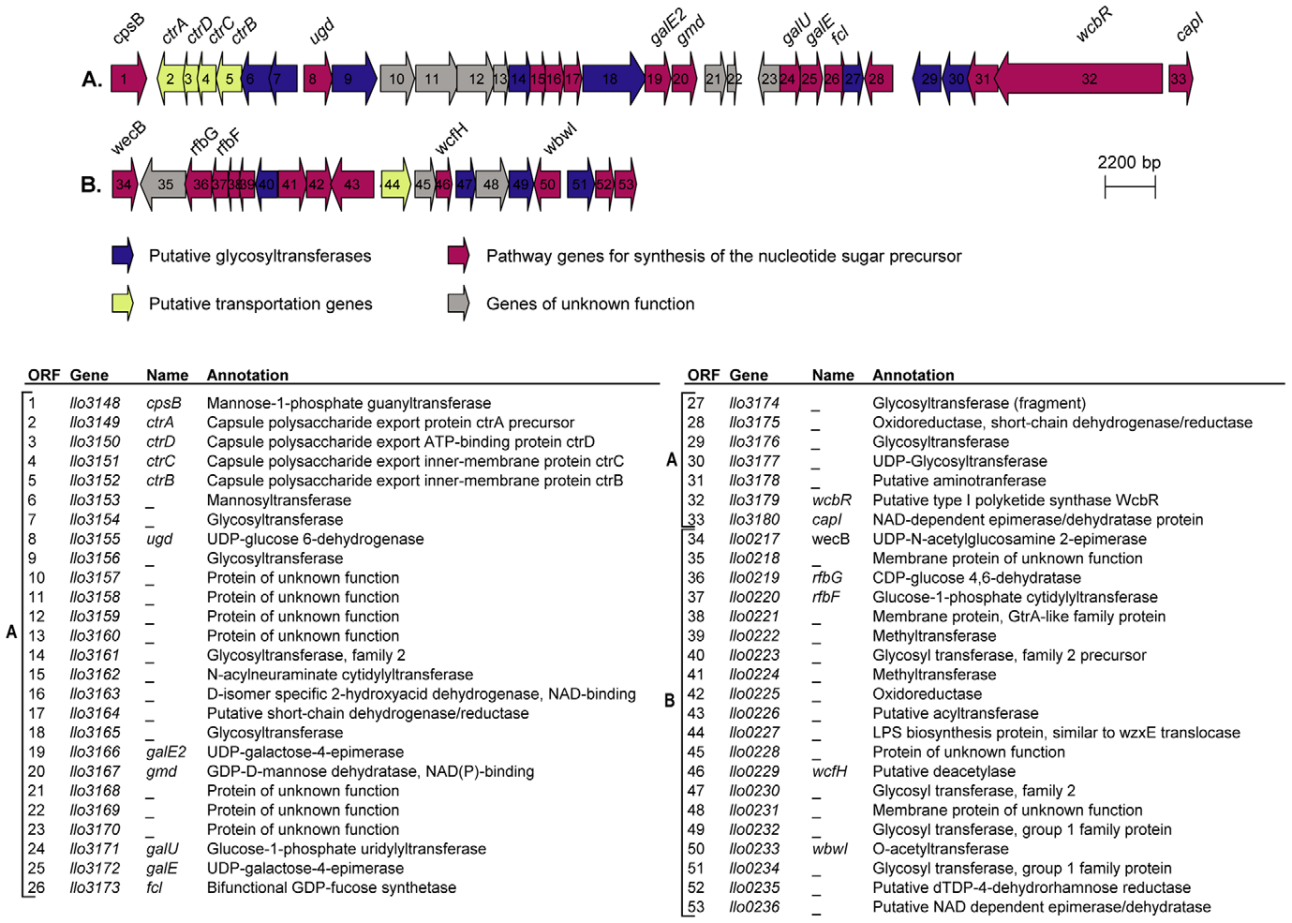
Putative capsule and LPS encoding loci in the genome of *L. longbeachae*. (A) 48 kb chromosomal region highly conserved in the four *L. longbeachae* genomes sequenced putatively encoding the capsular biosynthesis genes. (B) 24 kb chromosomal region differing between Sg1 and Sg2 isolates putatively encoding the lippolysaccaride biosynthesis genes of *L. longebachae*. Colors indicate different classes of genes: magenta, synthesis pathway of nucleoside sugar precursors; blue, glycosyltranferase; yellow transportation; grey, genes of unknown.

**Figure 4:**
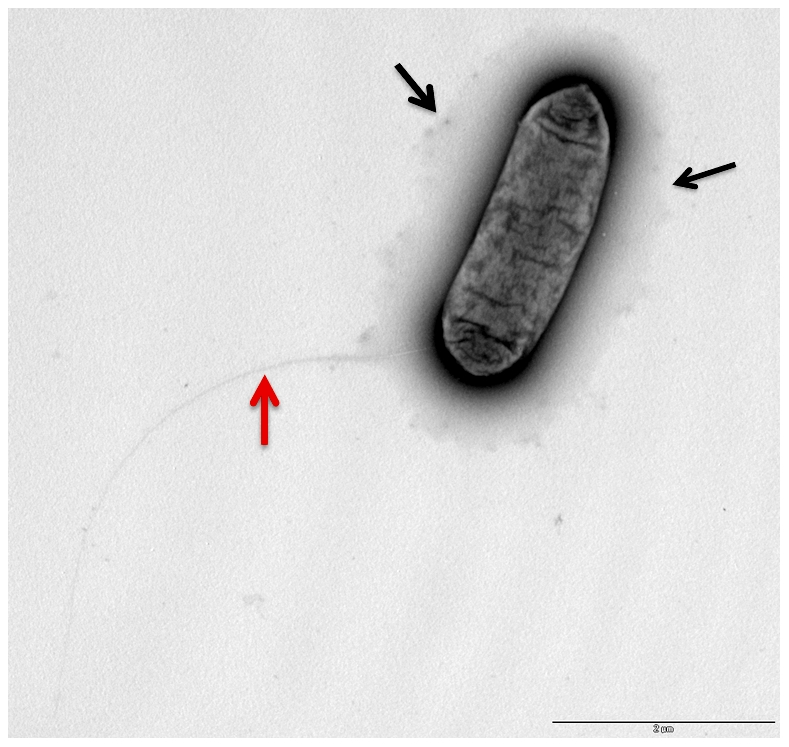
Electron microscopy showing the presence of capsule like structures. Transmission electron micrographs of *L. longbeache* cells cultured in BYE broth to post exponential growth phase (OD600 3.8). Black arrows, puative capsule structures, red Arrow, putative pili.

In the genome of *L. longbeachae* NSW150 we identified two gene clusters encoding proteins that are predicted to be involved in production of lipopolysaccharide (LPS) and/or capsule (Figure 3). Neither shared homology with the *L. pneumophila* LPS biosynthesis gene cluster. One region of 48 kb spans from *llo3148* to *llo3180* (Figure 3A) and the second of 24 kb from *llo0217* to *llo0236* (Figure 3B). In total they contain 26 genes for synthesis of the nucleotide sugar precursor, 12 genes encoding putative glycosyltransferases, 5 polysaccharide translocation genes including homologs of the *ctrABCD* capsule transport operon of *N. meningitidis*, and 10 genes of unknown function (Table S4). The finding that *L. longbeachae* might be encapsulated was further substantiated by electron microscopy analysis. Figure 4 shows that a capsule-like structure surrounds the bacteria.

Gene clusters encoding the core lipopolysaccharide of *L. pneumophila* and *L. longbeachae* are not conserved; however we identified in the genome of *L. longbeachae* homologs of *L. pneumophila* lipidA biosynthesis genes. Llo2684, Llo1461, Llo2686 and Llo0524 are homologous to LpxA, LpxB, LpxD and WaaM lipidA biosynthesis proteins with respectively 84%, 68%, 60% and 78% of identity. Predictions deduced from the sequence analysis of strain NSW150 did not clarify which region was coding for the LPS and which for the capsule. Further insight into the LPS and capsule encoding regions came from the comparison of this region among the four *L. longbeachae* genomes sequenced. The 24 kb region B is identical between the two Sg1 strains sequenced and identical between the two Sg2 strains analyzed, but the Sg1 and Sg2 strains differed from each other in an approximately 10 kb region carrying glycosyltransferases, methyltransferases, and LPS biosynthesis proteins (Figure S6). In contrast the putative capsule encoding region A was highly conserved among all four strains sequenced except for a region carrying three genes, that differed among all four strains independent of the Sg. However, as it is not known whether the Sg specificity of *L. longbeachae* is defined by its capsule or by LPS, further studies are necessary to clearly define the function of the proteins encoded in these two genomic regions.

### *L. longbeachae* does not encode flagella explaining differences in mouse susceptibility as compared to *L. pneumophila*

**Figure 5:**
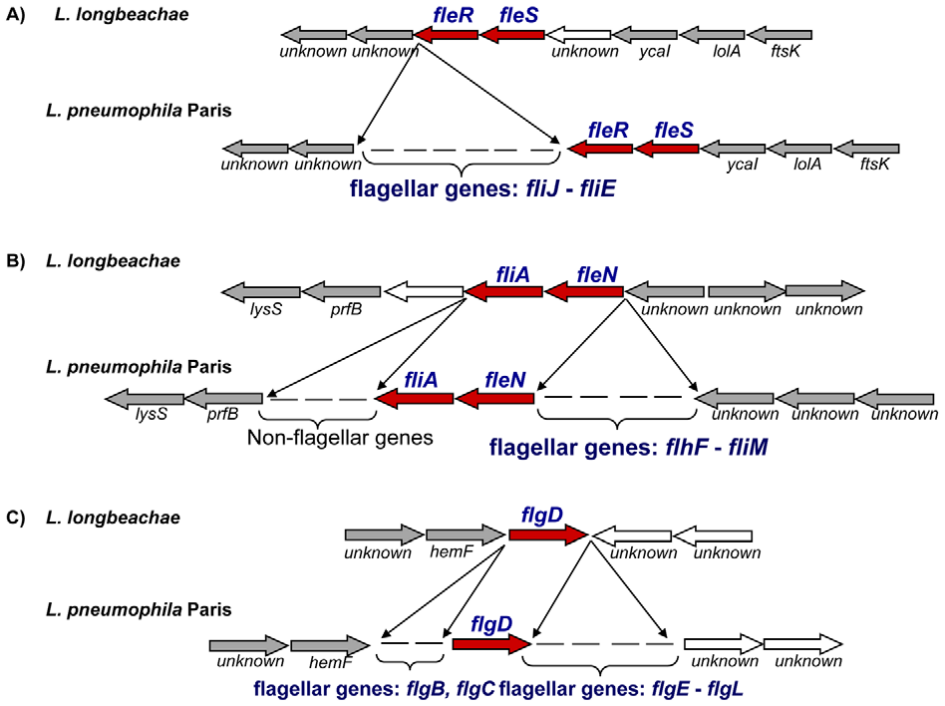
Alignment of the chromosomal regions of *L. pneumophila* and *L. longbeachae* coding the flagella biosynthesis genes. The comparison shows that all except the regulatory genes are missing in *L. longbeachae*. Red, conserved regulator encoding genes, grey arrows orthologous genes among the genomes, white arrows, non orthologues genes.

Cytosolic flagellin of *L. pneumophila* triggers Naip5-dependent caspase-1 activation and subsequent proinflammatory cell death by pyroptosis in C57BL/6 mice rendering these mice resistant to infection with *L. pneumophila* [22]–[24], [59]–[62]. In contrast, caspase-1 activation does not occur upon infection of C57BL/6 and A/J mice macrophages with *L. longbeachae,* which is then able to replicate. One possible explanation has been that due to a lack of pore-forming activity, *L. longbeachae* flagellin may not have access to the cytoplasm of the macrophage where it is thought to be involved in caspase-1 activation. Alternatively, *L. longbeachae* flagellin may not be recognized by the Naip5 pathway [11]. Genome analysis clarified this issue, as we found that *L. longbeachae* does not carry any flagellar biosynthesis genes except the sigma factor FliA, the regulator FleN, the two-component system FleR/FleS and the flagellar basal body rod modification protein FlgD. Interestingly, as shown in Figure 5, all genes bordering flagellar gene clusters were conserved between *L. longbeachae* and *L. pneumophila*, suggesting deletion of these regions from the *L. longbeachae* genome. Furthermore, not a single homologue of flagellar biosynthesis genes could be identified in other parts of the genome. Analysis of the three additional genome sequences of strains *L. longbeachae* ATCC39642, 98072 and C-4E7 confirmed the results. To further investigate this unexpected result, we designed primers in the conserved flanking genes to analyze these genomic regions in 15 *L. longbeachae* strains. All strains tested, eleven of Sg1 and four of Sg2, displayed the same organization as the sequenced strain (Table S5). According to these results, we propose that *L. longbeachae* fails to activate caspase-1 due to the lack of flagellin, which may also partly explain the differences in mouse susceptibility to *L. pneumophila* and *L. longbeachae* infection. The putative *L. longbeachae* capsule may also contribute to this difference.

Although *L. longbeachae* does not encode flagella, it encodes a putative chemotaxis system. Chemotaxis enables bacteria to find favorable conditions by migrating towards higher concentrations of attractants. The chemotactic response is mediated by a two-component signal transduction pathway, with the histidine kinase CheA and the response regulator CheY, putatively encoded by the genes *llo3302* and *llo3303* respectively, in the *L. longbeachae* genome. Furthermore, two homologues of the ‘adaptor’ protein CheW (encoded by *llo3298, llo3300*) that associate with CheA or cytoplasmic chemosensory receptors are present. Ligand-binding to receptors regulates the autophosphorylation activity of CheA in these complexes. The CheA phosphoryl group is subsequently transferred to CheY, which then diffuses away to the flagellum where it modulates motor rotation. Adaptation to continuous stimulation is mediated by a methyltransferase CheR encoded by *llo3299* in *L. longbeachae*. Together, these proteins represent an evolutionarily conserved core of the chemotaxis pathway, common to many bacteria and archea [55],[63]. A similar chemotaxis system is also present in *L. drancourtii* LLAP12 [64] but it is absent from *L. pneumophila*. The flanking genomic regions are highly conserved among *L. longbeachae* and all *L. pneumophila* strains sequenced, suggesting that *L. pneumophila*, although it encodes flagella has lost the chemotaxis system encoding genes.

We also observed using electron microscopy (Figure 4) that *L. longbeachae* possesses a long pilus-like structure. Indeed, all genes necessary to code for type IV pili are present in the genome of *L. longbeachae* and are, with 63–88% amino acid similarity, highly conserved between *L. longbeachae* and *L. pneumophila*. Taken together genome analysis revealed interesting features of the *Legionella* genomes: both encode pilus-like structures, in contrast *L. longbeachae* encodes a chemotaxis system but no flagella, and *L. pneumophila* encodes flagella but no chemotaxis system. It will be an interesting aspect of future research to understand these particular features of the two *Legionella* species.

### The regulatory repertoire of *L. longbeachae* suggests different adaptation mechanisms as compared to *L. pneumophila*

Similar to the *L. pneumophila* genomes and consistent with its intracellular lifestyle, the regulatory repertoire of *L. longbeachae* is rather small. Genome analysis identified 121 transcriptional regulators (113–116 in the four sequenced *L. pneumophila* genomes), which represent only 3.0% of the predicted genes (Table S6). Similar to *L. pneumophila, L. longbeachae* encodes six putative sigma factors, RpoD, RpoH, RpoS, RpoN, FliA and the ECF-type sigma factor RpoE.

The most abundant class of regulators of *L. pneumophila* is the GGDEF/EAL family (24 or 23 in all *L. pneumophila* genomes sequenced). This is significantly different in *L. longbeachae*, as we identified only 14 GGDEF/EAL domain-containing regulators, despite the larger size of the *L. longbeachae* genome. Furthermore, this group of regulators may fulfill specific functions in *L. longbeachae*, since most of the regulators possess no orthologs in the *L. pneumophila* genomes (Table S6). The function of these regulators in *L. pneumophila* and *L. longbeachae* is unknown, but in other bacteria these regulators play a role in aggregation, biofilm formation, twitching motility or flagella regulation. In *L. pneumophila* it was suggested, as deduced from gene expression analysis, that some of the GGDEF/EAL regulators may play a role in modulating flagella expression [65],[66], thus the lower number of GGDEF/EAL domain-containing proteins of *L. longbeachae* may in part be related to the missing flagellum.

Another difference in the regulatory repertoire of the two *Legionella* species was observed for two component systems. There are 14 response regulators and 13 histidine kinases in *L. pneumophila*, and 17 response regulators and 16 histidine kinases in the *L. longbeachae* genome, but only half of the *L. longbeachae* response regulators possess an ortholog in *L. pneumophila.* For example the recently described two-component system LqsS/LqsR that is part of a quorum sensing system in *L. pneumophila* is missing in *L. longbeachae* [67]–[69]. Two-component systems are involved in signal transduction pathways that enable bacteria to sense, respond, and adapt to a wide range of environments, stressors, and growth conditions [70]. Different two-component systems may be linked to the different environments to which *L. longbeachae* has to adapt compared to *L. pneumophila.*

In *L. longbeachae,* cyclic AMP may also transduce cellular signals as the genome encodes eight class III adenylate cyclases (Llo0181, Llo1751, Llo2196, Llo1669, Llo0753, Llo1197, Llo1216, Llo3304) of which only one (Llo0181) is also conserved in *L. pneumophila*. LadC, an adenylate cyclase of *L. pneumophila* that was shown to have a significant role in the initiation of infection *in vitro* and *in vivo* [71], is absent from *L. longbeachae*. As shown for *Pseudomonas aeruginosa,* these class III adenylate cyclases may sense environmental signals ranging from nutritional content of the surrounding media to the presence of host cells and control virulence gene expression accordingly [72]. Furthermore, 13 proteins containing cAMP binding motifs were identified, only one of which is shared with *L. pneumophila*, again indicating specific regulatory circuits for *L. longbeachae*. This high number of proteins that may sense cAMP indicates the potential importance of this signaling molecule in *L. longbeachae*.

In contrast, the regulators shown to be important for growth phase and life cycle dependent gene expression, such as the two component system LetA/LetS (Lllo2653/llo1235), the RNA-binding protein CsrA (Llo2071), the two small RNAs RsmY and RsmZ regulating CsrA [66],[73], SpoT (Llo0908) and RelA (Llo1756) are conserved in *L. longbeachae.* Likewise, the two-component systems PmrAB (Llo1159/Llo1158) and CpxRA (Llo1781/Llo1782) that regulate the Dot/Icm T4SS system and some of its substrates are both conserved in *L. longbeachae* [74]–[76].

### Global gene expression analysis reveals differences in the *L. longbeachae* and *L. pneumophila* life cycles

It has been shown in several studies that *L. pneumophila* exhibits at least two developmental stages, a replicative/avirulent and a transmissive/virulent phase that are each characterized by the expression of specific traits [9]. These stages are also reflected in a major shift in the gene expression program of *L. pneumophila* between the two phases of its life cycle [65]. In order to investigate, whether *L. longbeachae* had a similar biphasic life cycle we studied its gene expression program in exponential and post exponential growth phase *in vitro*. A multiple-genome microarray was constructed containing 10 692 gene-specific oligonucleotides representing 3567 genes predicted in the genome and on the plasmid and 3010 oligonucleotides specific for intergenic regions. RNA of *in vitro* grown bacteria was sampled at OD 2.5 (exponential growth) and at OD 3.7 (post exponential growth) and the global gene expression program was compared.

Overall, 187 genes in *L. longbeachae* were upregulated in the exponential (E) phase (likewise, downregulated in the postexponential phase, Table S7), and 313 genes were upregulated in the postexponential (PE) phase (downregulated in the E phase, Table S8). Real-time PCR analysis of selected genes validated the microarray results (data not shown). If we compare these results to those obtained for *L. pneumophila* grown *in vitro* [65], we observed several differences. In *L. pneumophila* strain Paris 543 genes are upregulated in E phase. Of the genes present in both genomes 270 are only upregulated in *L. pneumophila* but not in *L. longbeachae*. The 117 genes that are upregulated in both species in exponential phase include many ribosomal proteins, the genes belonging to the ATP synthase machinery (*atp* genes), the NADH deshydrogenase (*nuo* genes), most of the genes involved in translocation systems (*sec* genes) and several enzymatic activities (Table S7). However, several metabolic pathways clearly induced in E phase in *L. pneumophila* are not induced in *L. longbeachae*. These include the formyl THF biosynthesis, the purine and pyrimidine and the tetrahydrofolate biosynthesis pathways. Furthermore, genes coding for several chaperones (DnaJ, DnaK or GroES), the regulatory protein RecX and several proteins related to starvation and stress are not upregulated in E phase *L. longbeachae*. There are only 11 genes specific for *L. longbeachae* and induced in E phase, all of which code proteins for which no function could be predicted.

In PE phase 313 genes are upregulated in *L. longbeachae*, of which only 53 are also among the 441 PE phase genes of *L. pneumophila*. Interestingly, 208 of the genes upregulated in PE in *L. longbeachae* have no orthologs in *L. pneumophila,* and for 70% of these no function could be predicted. Thus the response of *L. longbeachae* to PE phase growth is distinct from that of *L. pneumophila*. In particular we observed differences in the expression profiles of many factors known to be involved in *L. pneumophila* virulence. For example, of the genes coding putative substrates of the Dot/Icm secretion system only few, *sidC* (*llo3098*), *sdhB* (*llo2439*), *sidE* homologue (*llo2210*), *sdeC/laiC* (*llo3092*) and *sdeB/laiB* (*llo3095*) are upregulated in post-exponential phase. However, several of the newly identified putative substrates are induced in *L. longbeachae* in PE phase. These comprise seven proteins homologous to Sid proteins of *L. pneumophila* (Llo0424, Llo0426, Llo2210, Llo2439, Llo3092, Llo3095 and Llo3098), three genes coding homologues of EnhA (*llo0852, llo1475* and *llo2343*), three ankyrin proteins (Llo0115, Llo1646 and Llo1715) and a putative serine threonine kinase (Llo1139). However, clear differences in gene expression between *L. pneumophila* and *L. longbeachae* exist and the switch from replicative to transmissive phase seems to be less pronounced in *L. longbeachae* than in *L. pneumophila*. Interestingly, the genes coding the stationary phase sigma factor RpoS and the sigma factor 28 (FliA) and CsrA, all involved in the regulation of the biphasic life cycle of *L. pneumophila* are not differentially regulated in *L. longbeachae*. In contrast, seven GGDEF/EAL domain-containing regulators (*llo0090, llo1253, llo1377, llo2005, llo3125, llo3392* and *llo3414*) and four cAMP binding proteins (*llo3395, llo2387, llo2141* and *llo1336*) are induced in PE phase. Thus cyclic di-GMP and cAMP may be important signaling molecules for regulating PE phase traits of *L. longbeachae*. According to our transcriptome analysis, the switch in the lifecycle of *L. longbeachae* appears less pronounced as compared to *L. pneumophila*, and regulation may be achieved mainly by secondary messenger molecules.

### Concluding remarks

*L. longbeachae* is the second leading cause of Legionnaires' disease in the world and a major cause of pneumonia in Australia and New Zealand. Yet, still very little is known about its virulence strategies and the genetic basis of virulence and niche adaptation. Analysis of the genome sequences of four *L. longbeachae* strains and its comparison with the published *L. pneumophila* genomes has uncovered important differences in the genetic repertoire of the two species and suggests different strategies for intracellular replication and niche adaptation.

Similar to *L. pneumophila*, *L. longbeachae* encodes a type IVB secretion system homologous to the Dot/Icm system. Inactivation of the type IV secretion system, through deletion of the *dotA* gene, showed that it is essential for virulence, as the *dotA* mutant had a severe growth defect in *A. castellanii* infection and could not establish an infection in the lungs of A/J mice. Despite this resemblance to *L. pneumophila*, the secreted effectors are very different as only 44% of the known *L. pneumophila* substrates were conserved in *L. longbeachae*. However, like *L. pneumophila*, many of them have eukaryotic domains or resemble eukaryotic proteins. Thus a large cohort of eukaryotic-like proteins was also a specific feature of the *L. longbeachae* genomes. An emerging theme in bacterial virulence is the evolution of virulence factors that can mimic the activities of Ras small GTPases (for a review see [77]). Small GTPases regulate unique biological functions of the cell as diverse as cell division/differentiation, actin cytoskeleton rearrangements, intracellular membrane trafficking. *L. pneumophila* produces the effector proteins RalF [78] and SidM/DrrA [40],[41] that activate small G-protein signaling cascades and interfere with host membrane trafficking. Here we identified *L. longbeachae* specific proteins belonging to the Rab subfamily of Ras small GTPases. These are the first prokaryotic Rab GTPases described and they may account for some of the differences in phagosome maturation between *L. longbeachae* and *L. pneumophila*. Overall, more than 3% of the *L. pneumophila* genome is thought to encode T4SS substrates that fulfill various functions, such as interfering with small GTPases of the early secretory pathway, disrupting phosphoinositide signaling or targeting microtubule-dependent vesicular transport. They may represent new strategies to interfere with host cell processes and may partly explain variations in the replication cycle of the two species.

An intriguing and unresolved question has been the susceptibility of C57BL/6 mice to *L. longbeachae* infection but their resistance to *L. pneumophila* infection. Only A/J mice that carry a particular Naip-5 allele are susceptible to *L. pneumophila* infection. Genome analysis has provided some insight into this question through the observation that *L. longbeachae* does not encode flagella, and thus does not trigger Naip5-dependent caspase-1 activation and subsequent proinflammatory cell death by pyroptosis [22]–[24], [59]–[62]. In contrast, *L. longbeachae* encodes a capsule that might be implicated in the recognition by the host immune system and which may provide some protection against killing by phagocytes. In *L. pneumophila*, expression of flagella is a hallmark of transmissive, virulent bacteria and a marker of its biphasic life cycle. In line with the absence of flagella, *L. longbeachae* also seems to have a less pronounced life cycle switch, as transcriptome analysis revealed a less dramatic change in gene expression compared to *L. pneumophila*. This result might explain the fact that intracellular proliferation of *L. longbeachae* is independent of the growth phase [11].

Previously we and others hypothesized, that *L. pneumophila* had acquired DNA by horizontal transfer or by convergent evolution during its co-evolution with free-living amoebae [25],[79] and that *L. pneumophila* uses molecular mimicry to subvert host functions [8],[80]. Presumably, *L. longbeachae* is not only able to interact with protozoa but also with plants, as several proteins present in plants and several enzymes which might confer the ability to degrade plant material were identified in the *L. longbeachae* genome.

Interestingly, the comparison of the genome sequence of four strains of *L. longbeachae* identified high gene content conservation unlike *L. pneumophila*. Furthermore, between strains of the same serogroup very few SNPs are present, most of them located in few plasticity zones, indicating recent expansion of this species. Based on these genome sequences, future comparative and functional studies will allow definition of the common and distinct survival tactics of pathogenic *Legionella* spp. and may open new ways to combat *L. pneumophila* and *L. longbeachae* infections.

## Materials and Methods

### Ethics statement

All animal experiments were conducted with approval from the University of Melbourne Animal Ethics committee application ID 0704867.3.

### DNA preparation and sequencing techniques

*L. longbeachae* strain NSW150 was grown on BCYE agar at 37°C for 3 days and chromosomal DNA was isolated by standard protocols. Cloning, sequencing and assembly were done as described [81]. One library (inserts of 1−3 kb) was generated by random mechanical shearing of genomic DNA, followed by cloning of the fragments into pcDNA-2.1 (Invitrogen). A scaffold was obtained by end-sequencing clones from a BAC library constructed as described [82] using pIndigoBac (Epicentre) as a vector. Plasmid DNA purification was done with a TempliPhi DNA sequencing template amplification kit (Amersham Biosciences). Sequencing reactions were done with an ABI PRISM BigDye Terminator cycle sequencing ready reactions kit and a 3730 Xl Genetic Analyzer (Applied Biosystems). We obtained and assembled 40299 sequences and performed finishing by adding 1125 additional sequences, as described earlier [81]. For draft genome sequencing of strains ATCC39642, 98072 and C-4E7 Illumina, shotgun libraries were generated from 5 µg of genomic DNA each using the standard Illumina protocols. Sequencing was carried out on an Illumina Genome Analyzer II as paired-end 36bp reads, following the manufacturer's protocols and with the Illumina PhiX sample used as control. Image analysis and base calling was performed by the Genome Analyser pipeline version 1.3 with default parameters.

### Annotation and sequence analysis

Definition of coding sequences and annotation were done as described [81] by using CAAT-box software [83] and MAGE (Magnifying Genomes) [84]. All predicted coding sequences were examined visually. Function predictions were based on BLASTp similarity searches and on the analysis of motifs using the PFAM, Prosite and SMART databases. We identified orthologous genes by reciprocal best-match BLAST and FASTA comparisons. Pseudogenes had one or more mutations that would prevent complete translation. Analysis of the three drafts genome sequences obtained by the Illumina technique was done as follows. First, to precisely determine the average insert size of mate-paired reads, we mapped the reads of each strain to the NSW150 sequence. Then, this value was used to give good mate-pair information to the de novo assembler. Short-reads were assembled *de novo* into contigs (without reference to any other sequence) using Velvet (version 0.7.55) [85]. To increase specificity and length of the generated contigs, we used the hash length (k-mer) of 27. Subsequently Mauve (version 2.3.0) [86] was used to build super contigs by aligning the de novo obtained contigs on the finished NSW150 sequence. Finally, for SNP discovery the program Maq (version 0.7.1) [87] was used for mapping the Solexa reads to the NSW150 reference. To detect high confidence SNPs, we only kept those SNPs that had a coverage of 10x to 300x. SNPs with a frequency lower than 80% were removed.

### Construction of a *dotA* mutant in strain *L. longbeachae* NSW150

To construct the *dotA* mutant strain, the chromosomal region containing the *dotA* gene was PCR-amplified with the primers dotA-for CTCGCGCATTGGAACTTTAT and dotA-rev TTCGCTCATAAACCGCTCTT. The product was cloned into the pGEM-T Easy vector (Promega) yielding pGEM-*dotA*. We performed inverse PCR on pGEM-*dotA* with primers dotAinv-for CGCGGATCCCCGCATTTAATACGCCAAAC and dotAinv-rev CGCGGATCCAAGGTTTTGCGTTGGATAGG containing *Bam*HI overhangs allowing internal deletion of 2582 bp in *dotA*. PCR product was digested with *Bam*HI and ligated to the kanamycin resistance cassette, which was amplified via PCR from the plasmid pGEM-Kan^R^, using primers containing *Bam*HI restriction sites at the ends (Kan-*Bam*HI-for TGCAGGTCGACTCAGAGGAT Kan-*Bam*HI-rev CGCGGATCCGAGCTCGGTACC). Linearized vector was electroporated in *L. longbeachae* to obtain *dotA*::Km mutant.

### *Acanthamoeba castellanii* infection assay

For *in vivo* growth of *L. longbeachae* and its *dotA* deletion mutant in *A. castellanii* we followed a protocol previously described [65]. In brief, three days old cultures of *A. castellanii* were washed in infection buffer (PYG 712 medium without proteose peptone, glucose, and yeast extract) and adjusted to 10^5^–10^6^ cells per ml. Stationary phase *Legionella* grown on BCYE agar, diluted in water were mixed with *A. castellanii* at a MOI of 0.1. After allowing invasion for 1 hour at 37°C the *A. castellanii* layer was washed twice with infection buffer (start point of time-course experiment). Intracellular multiplication was monitored using a 300 µl sample, which was centrifuged (14 000 rpm) and vortexed to break up amoeba. The number of colony forming units (CFU) of *Legionellae* was determined by plating on BCYE agar. Each infection was carried out in duplicates.

### Pulmonary infection of A/J mice with *L. longbeachae*

The comparative virulence of *L. longbeachae* NSW150 and the *dotA*::Km derivative within A/J mice was examined via competition assays and in single infections, as described previously [21],[34]. Briefly, 6- to 8-week-old female A/J mice (Jackson Laboratory, ME) were anesthetized and inoculated intratracheally with approximately 10^5^ CFU of each *L. longbeachae* strain under investigation. At 24 and 72 h following inoculation, mice were sacrificed and their lung tissue isolated. Tissue was homogenized, and complete host cell lysis was achieved by incubation in 0.1% saponin for 15 min at 37°C. Serial dilutions of the homogenate were plated onto both plain and antibiotic-selective BCYE agar to determine the number of viable bacteria and the ratio of wild-type to mutant bacteria colonizing the lung in mixed infections.

### Electron microscopy

Bacteria were transferred to Formvar-carbon-coated copper grids after glow discharged for 3′, stained with 1% uranyl acetate for 35sec, air dried and observed under a Jeol 1200EXII transmission electron microscope (Jeol, Tokyo, Japan) operated at 80kV. Digital acquisition was performed with a Mega View camera and the Analysis pro software version 3,1 (ELOISE, Roissy, France).

### PCR analysis

PCR for the regions containing the flagella biosynthesis coding genes in strain *L. pneumophila* Paris and *L. longbeachae* NSW150 were amplified with genomic DNA of strain Paris and NSW150 respectively. Primers were designed using the Primer 3 software to amplify a specific fragment of about 1 -3kb respectively for each region (melting temperatures 58°C). Amplification reactions were performed in a 50-µl reaction volume containing 6 ng of chromosomal DNA. The size of each PCR product was verified on agarose gels. Primers used are listed in Table S9.

### Transcriptome analysis

*L. longbeachae* strain NSW150 was cultured in N-(2-acetamido)-2-aminoethanesulphonic acid (ACES)-buffered yeast extract broth or on ACES-buffered charcoal –yeast (BCYE) extract agar at 37C. Total RNA was extracted as previously described [88]. *L. longbeachae* was harvested for RNA isolation at the exponential (OD 2.5) and post-exponential phase (OD 3.7). RNA was prepared from three independent cultures and each RNA sample was hybridized twice to the microarrays (dye swap). RNA was reverse-transcribed with Superscript indirect cDNA kit (Invitrogen) and labeled with Cy5 or Cy3 (Amersham Biosciences) according to the supplier's instructions. The microarray containing 13 710 60mer oligonucleotides specific for 3567 predicted genes of the genome, the plasmid and all intergenic regions longer than 200nts has been designed using the program OligoArray (http://berry.engin.umich.edu/oligoarray/). Based on these sequences a custom oligonucleotide array was manufactured (Agilent Technologies) with a final density of 15K. For hybridization, Cy3 and Cy5 target quantities were normalized at 150 pmol. Arrays were scanned using an Axon 4000B scanner with fixed PMT (PMT  =  550 for Cy3 and 650 for Cy5). Data were acquired and analyzed by Genepix Pro 5.0 (Axon Instrument). Spots were excluded from analysis in case of high local background fluorescence slide abnormalities or weak intensity. Data normalization and differential analysis were conducted using the R software (http://www.r-project.org). For each gene 3 probes were present on the microarray. Data for which at least 2 of the 3 probes gave a significant and non-contradictory result were taken into account. A loess normalization [89] was performed on a slide-by-slide basis (BioConductor package marray; http://www.bioconductor.org/packages/bioc/html/marray.html). Differential analysis was carried out separately for each comparison between two time points, using the VM method (VarMixt package [90], together with the Benjamini and Yekutieli [91] p-value adjustment method. The cut off for the expression ratio was set to either superior/equal to 2 or inferior/equal to 0.5 and the general ratio of expression of each gene was calculated as the average expression ratio from the different significant probes.

### URLs

The sequence and the annotation of the *L. longbeachae* NSW150 genome is accessible at the LegioList Web Server (http://genolist.pasteur.fr/LegioList and http://genolist.pasteur.fr/) and under the EMBL/Genbank Accession number: FN650140 the L. longbeachae NSW150 plasmid under the EMBL/Genbank Accession number: FN650141. Due to new regulations for genome sequence submissions to EMBL/Genbank the gene names (locus_tag), which are *e.g.* llo0001 in the article and in the Institut Pasteur databases had to be changed to LLO_0001 in the sequence submission. According to the standards for genome sequences published by Chain and colleagues [92] the *L. pneumophila* NSW150 genome sequence can be defined as “Finished” and the three Solexa genome sequence drafts can be defined as “High-Quality Draft”. The complete dataset for the transcriptome analysis is available at http://genoscript.pasteur.fr in a MIAME compliance public database maintained at the Institut Pasteur and was submitted to the ArrayExpress database maintained at http://www.ebi.ac.uk/microarray-as/ae/ under the Accession number: A-MEXP-1779.

## Supporting Information

Figure S1 Classification of the *L. longbeachae* CDS in the different COG groups. 2,506 CDS are classified in at least one COG group. Since several genes are assigned to multiple categories, the total number of assignments is greater than the number of ORFs in the genome.
(11.39 MB TIF)

Figure S2 Synteny plot of the chromosomes of *L. pneumophila* strain Paris and *L. longbeachae* NSW150. The plot was created using the mummer software package (http://mummer.sourceforge.net/).
(6.88 MB TIF)

Figure S3 Comparison of the plasmids identified in *L. longbeachae* and *L. pneumophila*. (A) Synteny LinePlot between the *L. longbeachae* plasmid and the plasmids of *L. pneumophila* strain Lens and Paris, respectively. Orthologous genes are defined by bi-directional blastP best hits (BDBH) or a blastP alignment threshold of 35% sequence identity over 80% of the length of the smaller protein. The gap parameter, representing the maximum number of consecutive genes that are not involved in a synteny group was 3. (B) Percentage of aminoacid identity among Tra proteins of the *L. longbeachae* and the *L. pneumophila* strain Lens as compared to the Tra region of strain Paris. (C) Venn diagram showing the common and specific gene content of the plasmids of *L. pneumophila* strains Paris, Lens and *L. longbeachae* NSW150.
(17.22 MB TIF)

Figure S4 Distribution of SNPs along the chromosome of *L. longbeachae* ATCC39462 (Sg1) and C-4E7 (Sg2) with respect to the completely sequence genome of *L. longbeachae* NSW 150 (Sg1). Outer circle, Mapping of SNPs between *L. longbeachae* Sg1 (NSW150) and Sg2 (C-4E7), central circle in green, sequence couverage of mapped reads of strain ATCC39462 on the NSW150 genome, inner circle; SNP distributon among the two Sg1 strains sequenced. 1426 SNPs are located in 7 genomic regions; region 1: *llo0557-llo0587* containing 112 SNPs; region 2: *llo0643-llo0653*, carries an integrase gene and contains 152 SNPs; region 3: *llo0814-llo0841* containing 38 SNPs; region 4: *llo0943-llo0952*, carries an integrase gene and contains 152 SNPs; region 5: *llo1813-llo1886*, carries many tra- like genes and contains 651 SNPs; region 6: *llo2119-llo2142*, contains 89 SNPs, region 7: *llo3148-llo3180*, carries genes encoding the putative capsule and contains 166 SNPs.
(10.54 MB TIF)

Figure S5 Aminoacid alignment of the RAS-domains of different *L. longbeachae* proteins identified in the genome of strain NSW150. PFAM was used to align the different sequences (http://pfam.sanger.ac.uk/).
(14.63 MB TIF)

Figure S6 Alignment of the putative LPS-encoding region of *L. longbeachae* Sg1 and Sg2 using the ARTEMIS comparison tool. Note the nearly perfect alignment of the four segments with only two regions differing between Sg1 and Sg2. Furthermore, the putative LPS-coding region of the two strains of the same Sg line perfectly up with a over 90% nucleotide identity. Specific regions and the predicted proteins encoded are depicted below.
(8.74 MB TIF)

Table S1 *L. longbeachae* NSW150 protein coding genes and their distribution within functional categories.
(0.03 MB DOC)

Table S2 Specific genes of *L. longbeachae* without orthologues in any of the four sequenced *L. pneumophila* genomes.
(0.50 MB DOC)

Table S3 Distribution of known and predicted Dot/Icm substrates of *L. pneumophila* in *L. longbeachae*.
(0.31 MB DOC)

Table S4 Putative capsule and LPS encoding genes in *L. longbeachae* and its comparison to *L. pneumophila* Paris.
(0.14 MB DOC)

Table S5 Analysis of the FlgD, FleR/S, and FliA/FleN encoding regions in *L. longbeachae*.
(0.05 MB DOC)

Table S6 Transcriptional regulators identified in *L. longbeachae* and their orthologs in *L. pneumophila*.
(0.30 MB DOC)

Table S7 Genes upregulated in *L. longbeachae* in exponential growth phase.
(0.23 MB DOC)

Table S8 Genes upregulated in *L. longbeachae* in post-exponetial growth phase.
(0.35 MB DOC)

Table S9 Sequence of primers used to amplify putative flagella gene encoding regions.
(0.04 MB DOC)
