# Supplementary figures and images for: Analysis of the Legionella longbeachae Genome and Transcriptome Uncovers Unique Strategies to Cause Legionnaires' Disease
Source: PLoS Genet. 2010 Feb 19;6(2):e1000851. doi: 10.1371/journal.pgen.1000851 (PMC2824747; doi:10.1371/journal.pgen.1000851)

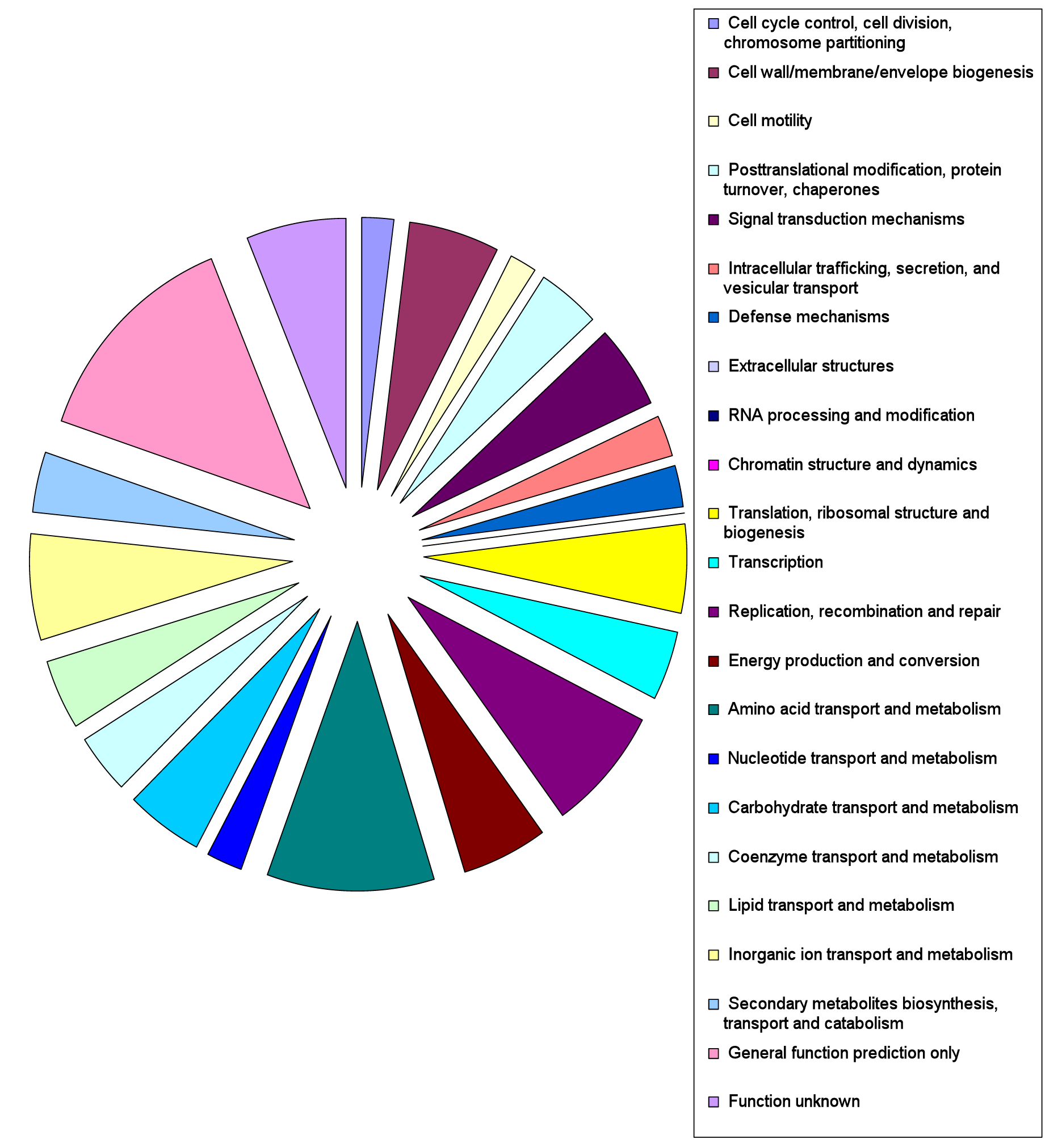

Supplement: Figure S1 — Classification of the L. longbeachae CDS in the different COG groups. 2,506 CDS are classified in at least one COG group. Since several genes are assigned to multiple categories, the total number of assignments is greater than the number of ORFs in the genome. (11.39 MB TIF) [file pgen.1000851.s001.tif]

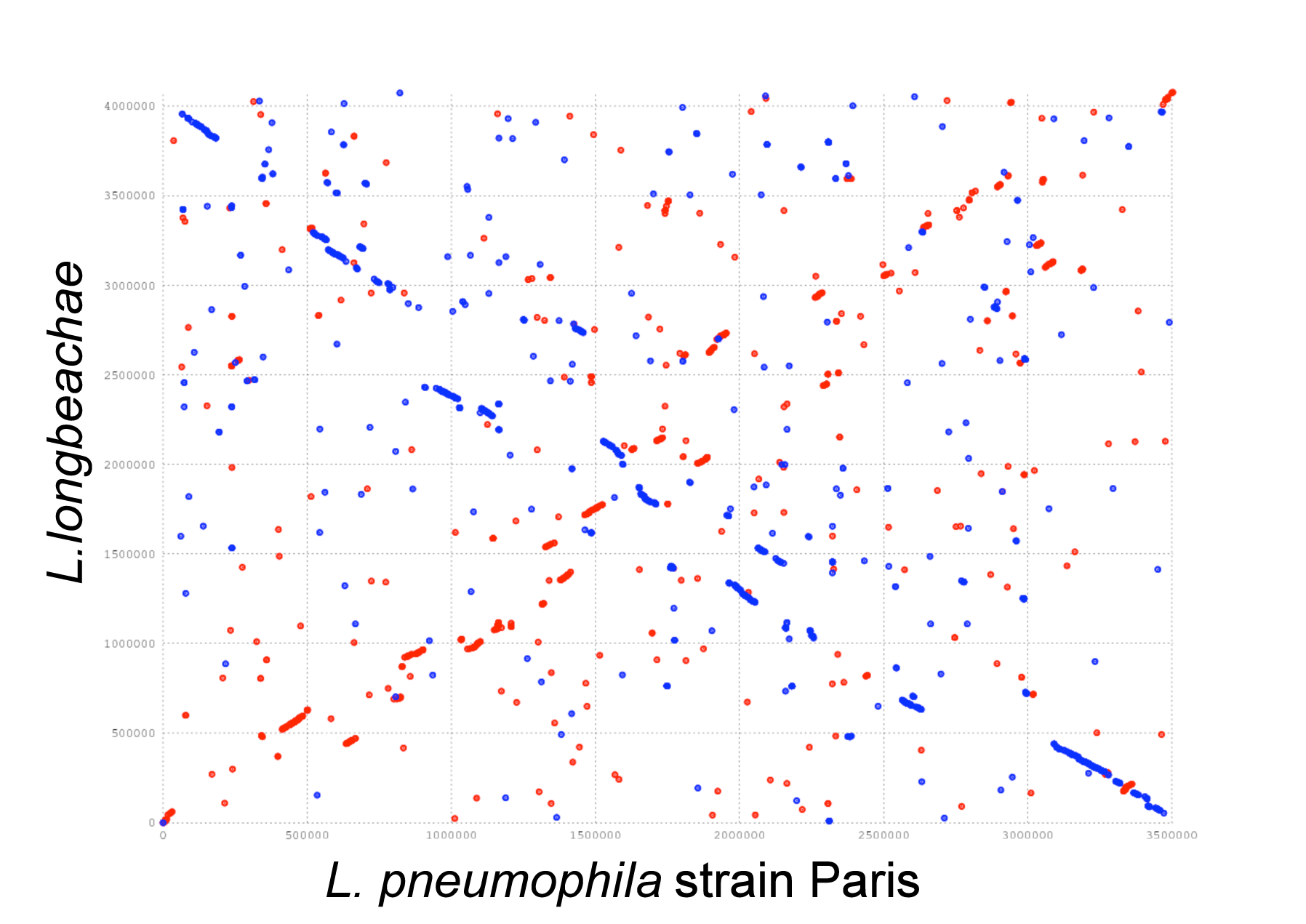

Supplement: Figure S2 — Synteny plot of the chromosomes of L. pneumophila strain Paris and L. longbeachae NSW150. The plot was created using the mummer software package (http://mummer.sourceforge.net/). (6.88 MB TIF) [file pgen.1000851.s002.tif]

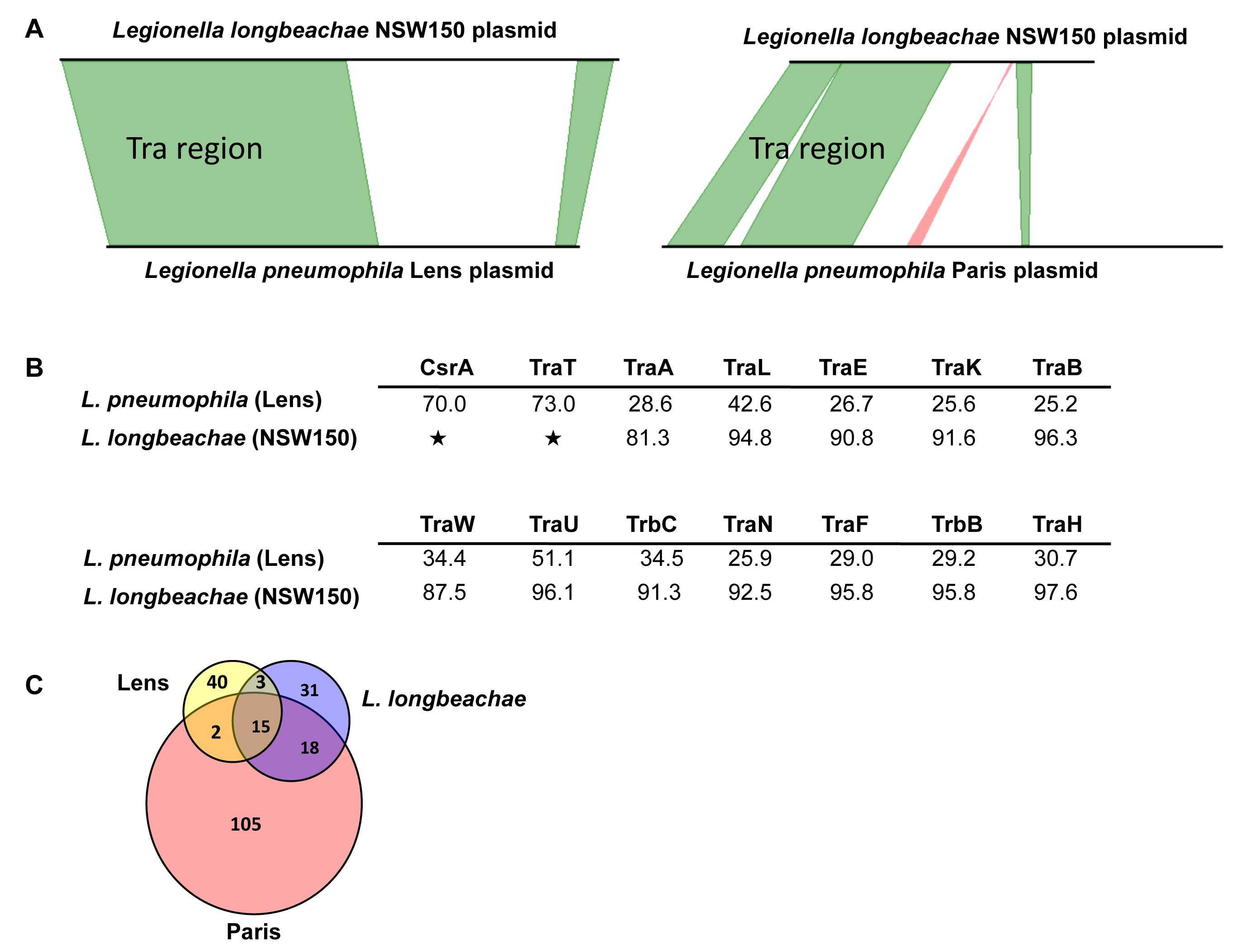

Supplement: Figure S3 — Comparison of the plasmids identified in L. longbeachae and L. pneumophila. (A) Synteny LinePlot between the L. longbeachae plasmid and the plasmids of L. pneumophila strain Lens and Paris, respectively. Orthologous genes are defined by bi-directional blastP best hits (BDBH) or a blastP alignment threshold of 35% sequence identity over 80% of the length of the smaller protein. The gap parameter, representing the maximum number of consecutive genes that are not involved in a synteny group was 3. (B) Percentage of aminoacid identity among Tra proteins of the L. longbeachae and the L. pneumophila strain Lens as compared to the Tra region of strain Paris. (C) Venn diagram showing the common and specific gene content of the plasmids of L. pneumophila strains Paris, Lens and L. longbeachae NSW150. (17.22 MB TIF) [file pgen.1000851.s003.tif]

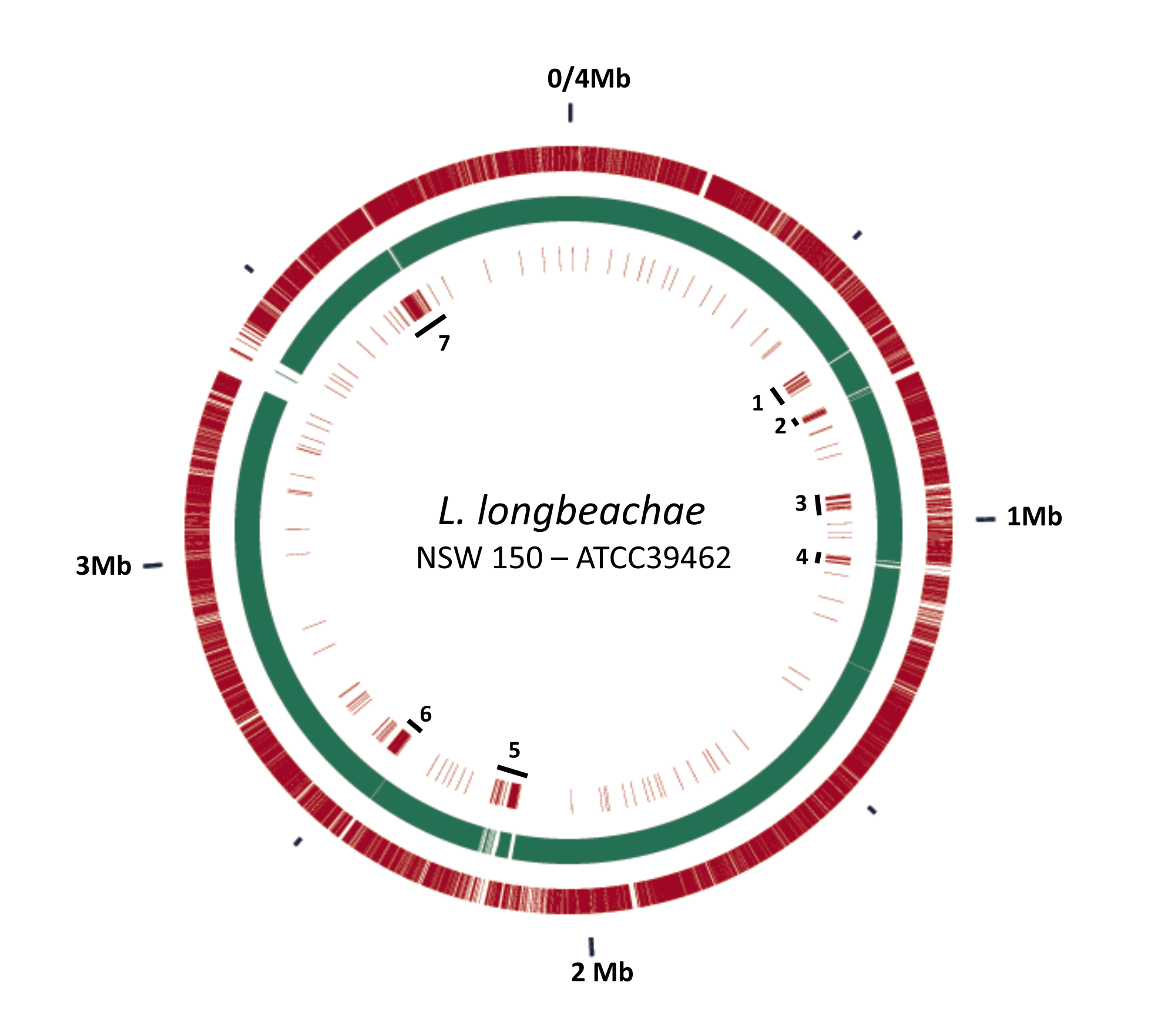

Supplement: Figure S4 — Distribution of SNPs along the chromosome of L. longbeachae ATCC39462 (Sg1) and C-4E7 (Sg2) with respect to the completely sequence genome of L. longbeachae NSW 150 (Sg1). Outer circle, Mapping of SNPs between L. longbeachae Sg1 (NSW150) and Sg2 (C-4E7), central circle in green, sequence couverage of mapped reads of strain ATCC39462 on the NSW150 genome, inner circle; SNP distributon among the two Sg1 strains sequenced. 1426 SNPs are located in 7 genomic regions; region 1: llo0557-llo0587 containing 112 SNPs; region 2: llo0643-llo0653, carries an integrase gene and contains 152 SNPs; region 3: llo0814-llo0841 containing 38 SNPs; region 4: llo0943-llo0952, carries an integrase gene and contains 152 SNPs; region 5: llo1813-llo1886, carries many tra- like genes and contains 651 SNPs; region 6: llo2119-llo2142, contains 89 SNPs, region 7: llo3148-llo3180, carries genes encoding the putative capsule and contains 166 SNPs. (10.54 MB TIF) [file pgen.1000851.s004.tif]

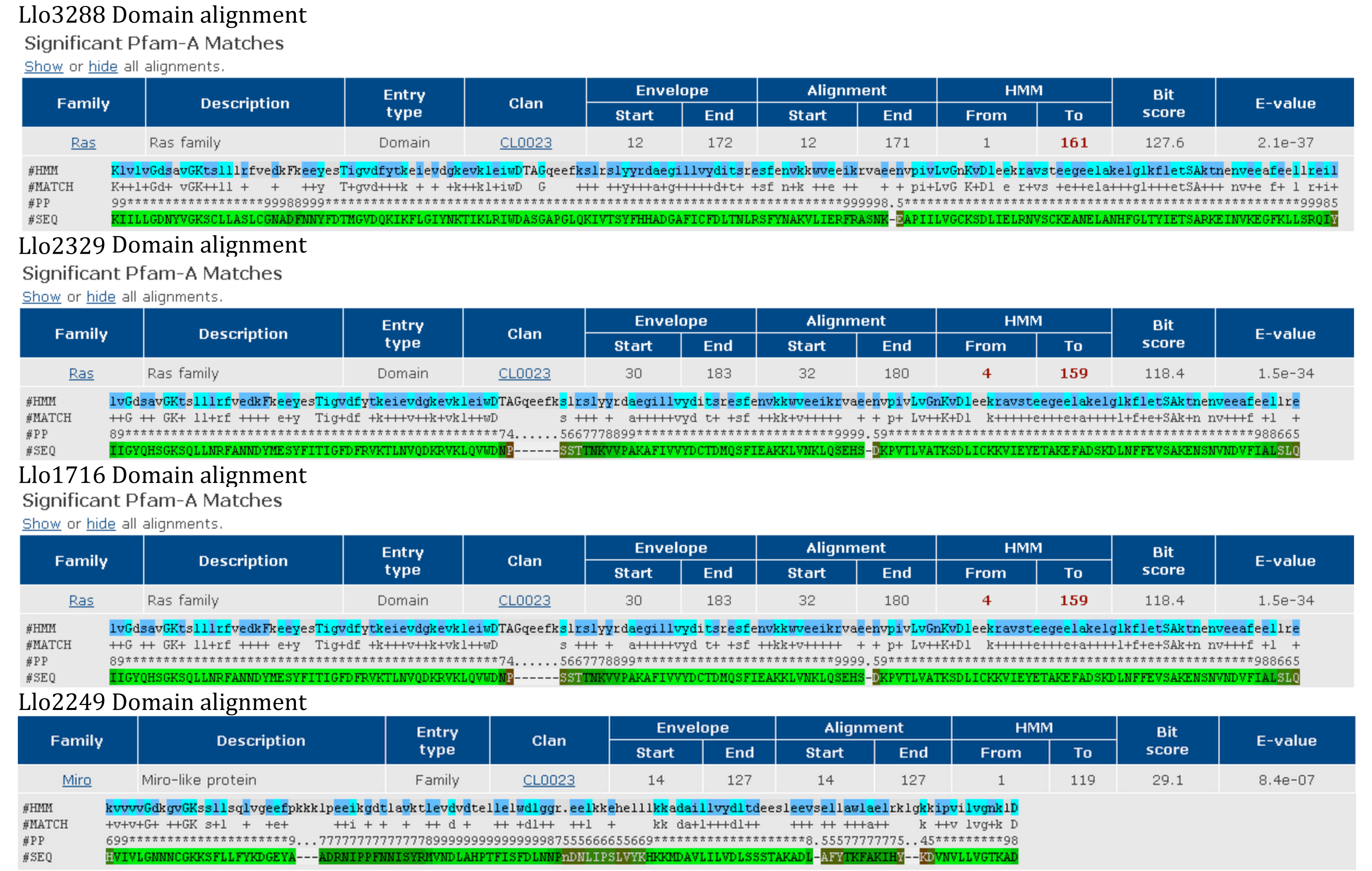

Supplement: Figure S5 — Aminoacid alignment of the RAS-domains of different L. longbeachae proteins identified in the genome of strain NSW150. PFAM was used to align the different sequences (http://pfam.sanger.ac.uk/). (14.63 MB TIF) [file pgen.1000851.s005.tif]

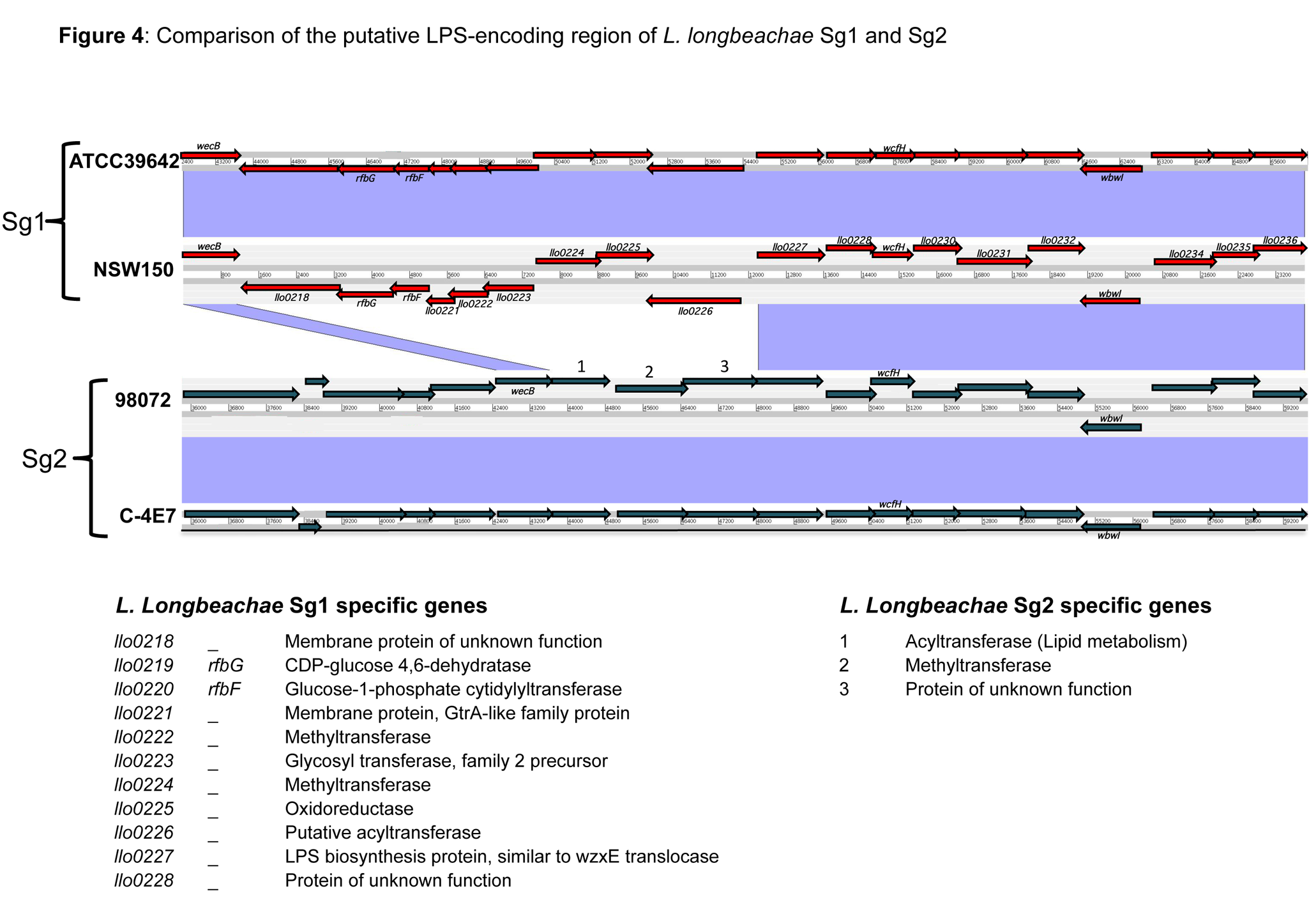

Supplement: Figure S6 — Alignment of the putative LPS-encoding region of L. longbeachae Sg1 and Sg2 using the ARTEMIS comparison tool. Note the nearly perfect alignment of the four segments with only two regions differing between Sg1 and Sg2. Furthermore, the putative LPS-coding region of the two strains of the same Sg line perfectly up with a over 90% nucleotide identity. Specific regions and the predicted proteins encoded are depicted below. (8.74 MB TIF) [file pgen.1000851.s006.tif]
