# Supplementary material for: Analysis of the Legionella longbeachae Genome and Transcriptome Uncovers Unique Strategies to Cause Legionnaires' Disease
Source: PLoS Genet. 2010 Feb 19;6(2):e1000851. doi: 10.1371/journal.pgen.1000851 (PMC2824747; doi:10.1371/journal.pgen.1000851)
Supplement: Table S1 — L. longbeachae NSW150 protein coding genes and their distribution within functional categories. (0.03 MB DOC) [file pgen.1000851.s007.doc]

**Table S1.** *L. longbeachae* NSW150 protein coding genes and their distribution within functional categories

1.1 Cell wall - outer membrane 117

1.2 Transport/binding proteins and lipoproteins 263

1.3 Sensors-signal transduction 32

1.4 Membrane bioenergetics (electron transport chain and ATP synthase) 98

1.5 Mobility and chemotaxis 10

1.6 Protein secretion 76

1.7 Cell division 24

1.8 Pili and other cell surface structures 21

2.1 Metabolism of carbohydrates and related molecules

2.1.1 Specific pathways 188

2.1.2 Main glycolytic pathways 21

2.1.3 TCA cycle 21

2.2 Metabolism of amino acids and related molecules 182

2.3 Metabolism of nucleotides and nucleic acids 88

2.4 Metabolism of lipids 112

2.5 Metabolism of coenzymes and prosthetic groups 82

2.6 Metabolism of phosphate 8

3.1 DNA replication 21

3.2 DNA restriction/modification and repair 53

3.3 DNA recombination 8

3.4 DNA packaging and segregation 18

3.5.1 Initiation 8

3.5.2 Regulation 85

3.5.3 Elongation 10

3.5.4 Termination 4

3.6 RNA modification 41

3.7.1 Ribosomal proteins 54

3.7.2 Aminoacyl-tRNA synthetases 27

3.7.3 Initiation 6

3.7.4 Elongation 7

3.7.5 Termination 4

3.8 Protein modification 28

3.9 Protein folding 12

4.1 Adaptation to atypical conditions 59

4.2 Detoxification 35

4.3 Toxins 3

4.4 Phage-related functions 18

4.5 Transposon, IS, Plasmid 152

4.6 Miscellaneous 117

4.7 Inorganic 5

5 Similar to unknown proteins

5.1 From Legionella 324

5.2 From other organisms 602

6 No similarity 467
