# Supplementary material for: Analysis of the Legionella longbeachae Genome and Transcriptome Uncovers Unique Strategies to Cause Legionnaires' Disease
Source: PLoS Genet. 2010 Feb 19;6(2):e1000851. doi: 10.1371/journal.pgen.1000851 (PMC2824747; doi:10.1371/journal.pgen.1000851)
Supplement: Table S2 — Specific genes of L. longbeachae without orthologues in any of the four sequenced L. pneumophila genomes. (0.50 MB DOC) [file pgen.1000851.s008.doc]

**Table S2:** Specific genes of *L. longbeachae* without orthologues* in any of the four sequenced *L. pneumophila* genomes

|  |  |  |
| --- | --- | --- |
| **Gene** | **Name** | **Predicted function** |
| *llo0005* | *_* | putative Uridine kinase |
| *llo0006* | *_* | protein of unknown function |
| *llo0009* | *_* | protein of unknown function |
| *llo0021* | *_* | protein of unknown function |
| *llo0023* | *_* | transposase (fragment) |
| *llo0024* | *_* | transposase (fragment) |
| *llo0026* | *_* | protein of unknown function |
| *llo0028* | *yihE* | putative kinase |
| *llo0029* | *hrpB* | Helicase |
| *llo0030* | *_* | protein of unknown function |
| *llo0031* | *_* | protein of unknown function |
| *llo0033* | *_* | protein of unknown function |
| *llo0035a* | *_* | transposase (fragment) |
| *llo0035b* | *_* | transposase (fragment) |
| *llo0036* | *_* | conserved protein of unknown function |
| *llo0037* | *_* | protein of unknown function |
| *llo0038* | *_* | protein of unknown function |
| *llo0040* | *_* | protein of unknown function |
| *llo0041* | *_* | protein of unknown function |
| *llo0042* | *_* | Similar to eukaryotic serine carboxypeptidase S28 family protein |
| *llo0044* | *_* | exported protein of unknown function |
| *llo4000* | *_* | protein of unknown function |
| *llo0049* | *_* | protein of unknown function |
| *llo0050* | *_* | exported protein of unknown function |
| *llo0054* | *_* | protein of unknown function |
| *llo0055* | *_* | protein of unknown function |
| *llo0056* | *_* | protein of unknown function |
| *llo0077* | *_* | protein of unknown function |
| *llo0078* | *_* | protein of unknown function |
| *llo0089* | *_* | protein of unknown function |
| *llo0092* | *_* | protein of unknown function |
| *llo0093* | *_* | protein of unknown function |
| *llo0094* | *_* | membrane protein of unknown function |
| *llo0095* | *_* | protein of unknown function |
| *llo0096* | *_* | Aspartate carbamoyltransferase (ATCase) (fragment) |
| *llo0097* | *_* | protein of unknown function |
| *llo0100* | *yqfA* | putative hemolysin, inner membrane subunit |
| *llo0101* | *ybcJ* | putative RNA-binding protein |
| *llo0102* | *_* | conserved protein of unknown function |
| *llo0103* | *_* | protein of unknown function |
| *llo0104* | *_* | fragment of cytidine/deoxycytidylate deaminase (part 1) |
| *llo0105* | *_* | protein of unknown function |
| *llo0106* | *_* | transposase |
| *llo0107* | *_* | conserved protein of unknown function |
| *llo0108* | *_* | protein of unknown function |
| *llo0109* | *_* | protein of unknown function |
| *llo0110* | *_* | conserved exported protein of unknown function |
| *llo0111* | *_* | protein of unknown function |
| *llo0114* | *_* | Leucine-rich repeat (LRR) protein (fragment) |
| *llo4002* | *_* | Leucine-rich repeat-containing protein 40 (fragment) |
| *llo0116* | *_* | membrane protein of unknown function |
| *llo0117* | *_* | membrane protein of unknown function |
| *llo0118* | *_* | protein of unknown function |
| *llo0120* | *_* | conserved protein of unknown function |
| *llo0122* | *_* | protein of unknown function |
| *llo0123* | *_* | protein of unknown function |
| *llo0134* | *_* | protein of unknown function |
| *llo0135* | *hsc* | Cytochrome c Hsc |
| *llo0139* | *_* | protein of unknown function |
| *llo4003* | *_* | protein of unknown function |
| *llo0155* | *_* | exported protein of unknown function |
| *llo0161* | *_* | membrane protein of unknown function |
| *llo0177* | *_* | protein of unknown function |
| *llo0178* | *_* | protein of unknown function |
| *llo0179* | *_* | protein of unknown function |
| *llo0183* | *_* | exported protein of unknown function |
| *llo0189* | *_* | protein of unknown function |
| *llo0193* | *_* | conserved protein of unknown function |
| *llo0204* | *_* | protein of unknown function |
| *llo0217* | *wecB* | UDP-N-acetylglucosamine 2-epimerase |
| *llo0218* | *_* | membrane protein of unknown function |
| *llo0219* | *rfbG* | CDP-glucose 4,6-dehydratase |
| *llo0220* | *rfbF* | Glucose-1-phosphate cytidylyltransferase (CDP-glucose pyrophosphorylase) |
| *llo0221* | *_* | membrane protein of unknown function |
| *llo0222* | *_* | conserved protein of unknown function |
| *llo0224* | *_* | conserved protein of unknown function |
| *llo0225* | *_* | protein of unknown function |
| *llo0227* | *_* | putative Lipopolysaccharide biosynthesis protein |
| *llo0228* | *_* | protein of unknown function |
| *llo0229* | *wcfH* | putative deacetylase |
| *llo0230* | *_* | protein of unknown function |
| *llo0231* | *_* | membrane protein of unknown function |
| *llo0232* | *_* | putative WbuH |
| *llo0233* | *wbwI* | O-acetyltransferase |
| *llo0234* | *_* | Glycosyl transferase, group 1 family protein |
| *llo0235* | *_* | conserved protein of unknown function |
| *llo0236* | *_* | Polysaccharide biosynthesis protein |
| *llo0237* | *_* | protein of unknown function |
| *llo0245* | *_* | protein of unknown function |
| *llo0247* | *_* | protein of unknown function |
| *llo0252* | *_* | protein of unknown function |
| *llo0253* | *_* | protein of unknown function |
| *llo0261* | *_* | protein of unknown function |
| *llo0303* | *_* | protein of unknown function |
| *llo0315* | *atpB* | V-type ATP synthase beta chain (V-type ATPase subunit B) |
| *llo0316* | *_* | V-type ATP synthase subunit D |
| *llo0317* | *_* | putative ATP synthase subunit I |
| *llo0318* | *_* | ATP synthase subunit K |
| *llo0319* | *_* | putative V-type ATPase, subunit E |
| *llo0320* | *_* | protein of unknown function |
| *llo0321* | *atpA* | V-type ATP synthase alpha chain 1 (V-type ATPase subunit A 1) |
| *llo0335* | *_* | Transcriptional regulator, MarR family |
| *llo0358* | *_* | protein of unknown function |
| *llo4004* | *_* | protein of unknown function |
| *llo0379a* | *_* | Fragment of putative non-ribosomal peptide synthase (N-terminal part)(part 1) |
| *llo4005* | *_* | Fragment of putative non-ribosomal peptide synthase (part 2) |
| *llo0379b* | *_* | Putative non-ribosomal peptide synthase (C-terminal part) part 3 |
| *llo0381* | *_* | exported protein of unknown function |
| *llo0382* | *_* | NAD-dependent epimerase/dehydratase |
| *llo0383* | *capL* | Protein capL |
| *llo0384* | *_* | membrane protein of unknown function |
| *llo0411* | *_* | protein of unknown function |
| *llo0412* | *_* | Glycosyl transferase |
| *llo0413* | *_* | protein of unknown function |
| *llo0427* | *_* | exported protein of unknown function |
| *llo0430* | *_* | conserved protein of unknown function |
| *llo0439* | *_* | protein of unknown function |
| *llo0440* | *btuE* | putative glutathione peroxidase |
| *llo0446* | *_* | exported protein of unknown function |
| *llo0447* | *_* | protein of unknown function |
| *llo0448* | *_* | protein of unknown function |
| *llo0450* | *_* | putative Ribokinase |
| *llo0452* | *_* | Aconitate hydratase |
| *llo0455* | *_* | protein of unknown function |
| *llo0459* | *_* | conserved protein of unknown function |
| *llo0471* | *_* | conserved protein of unknown function |
| *llo4006* | *rpmJ* | 50S ribosomal subunit protein L36 |
| *llo0512* | *_* | transposase (fragment) |
| *llo0513* | *_* | exported protein of unknown function |
| *llo0516* | *_* | protein of unknown function |
| *llo0517* | *_* | protein of unknown function |
| *llo0532* | *_* | exported protein of unknown function |
| *llo0538* | *emrB* | multidrug efflux system protein |
| *llo0539* | *emrA* | multidrug efflux system |
| *llo0540* | *_* | exported protein of unknown function |
| *llo4007* | *ecnA* | entericidin A membrane lipoprotein, antidote entericidin B |
| *llo0545* | *_* | RND efflux system, outer membrane lipoprotein, NodT family precursor |
| *llo0548* | *_* | protein of unknown function |
| *llo0571* | *_* | protein of unknown function |
| *llo0574* | *_* | protein of unknown function |
| *llo0587* | *_* | protein of unknown function |
| *llo4009* | *_* | conserved exported protein of unknown function |
| *llo0591* | *_* | protein of unknown function |
| *llo0595* | *_* | exported protein of unknown function |
| *llo0597* | *_* | protein of unknown function |
| *llo0608* | *_* | exported protein of unknown function |
| *llo0614* | *_* | protein of unknown function |
| *llo4010* | *_* | protein of unknown function |
| *llo0624* | *_* | protein of unknown function |
| *llo0625* | *_* | protein of unknown function |
| *llo0639* | *_* | exported protein of unknown function |
| *llo0640* | *_* | protein of unknown function |
| *llo0641* | *_* | protein of unknown function |
| *llo0642* | *_* | protein of unknown function |
| *llo0643* | *_* | conserved protein of unknown function |
| *llo0644* | *_* | protein of unknown function |
| *llo0647* | *_* | putative dCMP deaminase |
| *llo0649* | *_* | conserved protein of unknown function |
| *llo0652* | *_* | putative transcriptional regulator |
| *llo0654* | *_* | Virulence-associated protein I (fragment) |
| *llo0655* | *_* | Spermine synthase |
| *llo0656* | *_* | protein of unknown function |
| *llo0669* | *_* | conserved protein of unknown function |
| *llo0670* | *_* | conserved exported protein of unknown function |
| *llo0674* | *_* | protein of unknown function |
| *llo0675c* | *_* | membrane protein of unknown function |
| *llo0679* | *_* | protein of unknown function |
| *llo0680* | *_* | protein of unknown function |
| *llo0681* | *_* | conserved membrane protein of unknown function |
| *llo0682* | *_* | exported protein of unknown function |
| *llo4012* | *_* | protein of unknown function |
| *llo0683* | *_* | protein of unknown function |
| *llo0684* | *_* | protein of unknown function |
| *llo0685* | *asnC* | DNA-binding transcriptional dual regulator |
| *llo0686* | *asnA* | asparagine synthetase A |
| *llo0687* | *_* | protein of unknown function |
| *llo0688* | *_* | protein of unknown function |
| *llo0689* | *_* | protein of unknown function |
| *llo0693* | *_* | protein of unknown function |
| *llo0694* | *_* | conserved protein of unknown function |
| *llo0699* | *_* | protein of unknown function |
| *llo0700* | *_* | protein of unknown function |
| *llo0701* | *_* | protein of unknown function |
| *llo0702* | *_* | protein of unknown function |
| *llo0704* | *_* | protein of unknown function |
| *llo0705* | *_* | protein of unknown function |
| *llo0706* | *_* | protein of unknown function |
| *llo0707a* | *_* | protein of unknown function |
| *llo0707b* | *_* | protein of unknown function |
| *llo0708* | *_* | protein of unknown function |
| *llo0709* | *_* | protein of unknown function |
| *llo0712* | *_* | exported protein of unknown function |
| *llo0713* | *_* | conserved protein of unknown function |
| *llo0714* | *_* | protein of unknown function |
| *llo0720* | *_* | protein of unknown function |
| *llo0724* | *_* | conserved membrane protein of unknown function |
| *llo0725* | *_* | protein of unknown function |
| *llo0726* | *_* | D-isomer specific 2-hydroxyacid dehydrogenase family protein |
| *llo0727* | *_* | L-3-phosphoserine phosphatase |
| *llo0728* | *_* | membrane protein of unknown function |
| *llo0730* | *_* | conserved protein of unknown function |
| *llo0736* | *_* | protein of unknown function |
| *llo0737* | *_* | protein of unknown function |
| *llo4014* | *_* | fragment of protein of unknown function |
| *llo0738* | *_* | protein of unknown function |
| *llo0739* | *_* | conserved exported protein of unknown function |
| *llo0740* | *_* | Isochorismatase family protein |
| *llo0741* | *_* | putative membrane protein |
| *llo0744* | *_* | Nitroreductase |
| *llo0745* | *_* | protein of unknown function |
| *llo0746* | *_* | membrane protein of unknown function |
| *llo0747* | *_* | Muramoyltetrapeptide carboxypeptidase |
| *llo0748* | *_* | protein of unknown function |
| *llo0753* | *_* | putative Adenylate cyclase |
| *llo0758* | *_* | conserved protein of unknown function |
| *llo0759* | *_* | protein of unknown function |
| *llo0760* | *_* | putative Ribonuclease Z |
| *llo0761* | *_* | protein of unknown function |
| *llo0762* | *_* | protein of unknown function |
| *llo0766* | *_* | protein of unknown function |
| *llo0769* | *_* | membrane protein of unknown function |
| *llo0773* | *_* | membrane protein of unknown function |
| *llo0775* | *_* | protein of unknown function |
| *llo0778* | *_* | DEAD/DEAH box helicase-like |
| *llo0784* | *_* | exported protein of unknown function |
| *llo0785* | *_* | exported protein of unknown function |
| *llo0786* | *_* | membrane protein of unknown function |
| *llo0787* | *_* | protein of unknown function |
| *llo0788* | *_* | putative Protein cysZ homolog |
| *llo0793* | *_* | protein of unknown function |
| *llo0797* | *_* | protein of unknown function |
| *llo0799* | *_* | conserved exported protein of unknown function |
| *llo0800* | *_* | protein of unknown function |
| *llo4015* | *_* | protein of unknown function |
| *llo0822* | *_* | protein of unknown function |
| *llo0842* | *_* | protein of unknown function |
| *llo0862* | *_* | protein of unknown function |
| *llo0863* | *_* | protein of unknown function |
| *llo0864* | *_* | putative aminoacid/polyamine transporter, permease protein |
| *llo0870* | *_* | membrane protein of unknown function |
| *llo0871* | *_* | conserved protein of unknown function |
| *llo4016* | *_* | protein of unknown function |
| *llo0882* | *_* | protein of unknown function |
| *llo0885* | *galU* | glucose-1-phosphate uridylyltransferase |
| *llo0894* | *_* | exported protein of unknown function |
| *llo0900* | *_* | protein of unknown function |
| *llo4017* | *_* | protein of unknown function |
| *llo0901* | *_* | conserved protein of unknown function |
| *llo0902* | *_* | protein of unknown function |
| *llo0915* | *_* | exported protein of unknown function |
| *llo0916* | *_* | protein of unknown function |
| *llo0917* | *_* | protein of unknown function |
| *llo0918* | *_* | protein of unknown function |
| *llo0919* | *_* | protein of unknown function |
| *llo0925* | *_* | protein of unknown function |
| *llo0926* | *_* | putative Rieske 2Fe-2S domain protein |
| *llo0927* | *_* | conserved protein of unknown function |
| *llo0928* | *_* | putative biotin carboxylase |
| *llo4018* | *_* | protein of unknown function |
| *llo0938* | *_* | conserved membrane protein of unknown function |
| *llo0940* | *_* | protein of unknown function |
| *llo0941* | *_* | putative DNA helicase |
| *llo0942* | *_* | protein of unknown function |
| *llo0943* | *_* | putative Phage/plasmid maintenance toxin/antidote system protein (Toxin) |
| *llo0944* | *_* | protein of unknown function |
| *llo0950* | *_* | conserved protein of unknown function |
| *llo0961a* | *_* | putative Acetoacetyl-CoA reductase |
| *llo0962* | *_* | conserved protein of unknown function |
| *llo0963* | *_* | membrane protein of unknown function |
| *llo0964* | *_* | protein of unknown function |
| *llo0965* | *_* | exported protein of unknown function |
| *llo0966* | *_* | exported protein of unknown function |
| *llo0967* | *_* | protein of unknown function |
| *llo0969* | *_* | conserved protein of unknown function |
| *llo0970* | *ydcN* | putative DNA-binding transcriptional regulator |
| *llo0971* | *_* | protein of unknown function |
| *llo0972* | *_* | protein of unknown function |
| *llo0974* | *_* | putative alcohol dehydrogenase BadC |
| *llo0978* | *_* | conserved protein of unknown function |
| *llo4019* | *_* | putative Multidrug resistance ABC transporter MsbA (fragment) |
| *llo0987b* | *_* | protein of unknown function |
| *llo0987a* | *_* | protein of unknown function |
| *llo0988* | *_* | protein of unknown function |
| *llo0989* | *_* | transposase (fragment) |
| *llo0990* | *_* | protein of unknown function |
| *llo0992* | *_* | protein of unknown function |
| *llo0993* | *_* | protein of unknown function |
| *llo0995* | *_* | protein of unknown function |
| *llo0996* | *_* | protein of unknown function |
| *llo0999* | *_* | protein of unknown function |
| *llo1002* | *_* | protein of unknown function |
| *llo1005* | *_* | protein of unknown function |
| *llo1006* | *_* | putative N-acetylglucosamine-6-phosphate deacetylase |
| *llo1007* | *_* | protein of unknown function |
| *llo1009* | *_* | protein of unknown function |
| *llo1010* | *_* | exported protein of unknown function |
| *llo1012* | *_* | conserved protein of unknown function |
| *llo1013* | *_* | protein of unknown function |
| *llo1018* | *_* | putative ABC transporter-like |
| *llo1022* | *_* | exported protein of unknown function |
| *llo1024* | *_* | putative Hydroxyethylthiazole kinase |
| *llo1026* | *_* | conserved protein of unknown function |
| *llo1027* | *ydiI* | putative esterase |
| *llo1029* | *_* | protein of unknown function |
| *llo1031* | *_* | conserved protein of unknown function |
| *llo1033* | *_* | exported protein of unknown function |
| *llo1043* | *_* | protein of unknown function |
| *llo1063* | *_* | protein of unknown function |
| *llo1064* | *_* | protein of unknown function |
| *llo1065* | *_* | protein of unknown function |
| *llo1066* | *_* | protein of unknown function |
| *llo1068* | *_* | protein of unknown function |
| *llo1083* | *_* | exported protein of unknown function |
| *llo1090* | *_* | protein of unknown function |
| *llo1091* | *_* | protein of unknown function |
| *llo4021* | *_* | transposase (fragment) |
| *llo1092* | *_* | protein of unknown function |
| *llo1093* | *_* | protein of unknown function |
| *llo4022* | *_* | protein of unknown function |
| *llo1094* | *_* | protein of unknown function |
| *llo1097* | *_* | protein of unknown function |
| *llo1103* | *_* | putative RNA-directed DNA polymerase (Reverse transcriptase) |
| *llo1122* | *_* | protein of unknown function |
| *llo1123* | *_* | protein of unknown function |
| *llo1129* | *_* | protein of unknown function |
| *llo1132b* | *_* | protein of unknown function |
| *llo1133* | *_* | membrane protein of unknown function |
| *llo1134* | *_* | protein of unknown function |
| *llo1137* | *_* | protein of unknown function |
| *llo1138* | *_* | protein of unknown function |
| *llo1139* | *_* | protein of unknown function |
| *llo1141* | *_* | protein of unknown function |
| *llo1142* | *_* | protein of unknown function |
| *llo1143* | *_* | protein of unknown function |
| *llo1162* | *_* | putative Galacturan 1,4-alpha-galacturonidase |
| *llo1168* | *_* | protein of unknown function |
| *llo1169* | *_* | protein of unknown function |
| *llo1177* | *_* | membrane protein of unknown function |
| *llo1178* | *emrA* | multidrug efflux system |
| *llo1179* | *_* | exported protein of unknown function |
| *llo1182* | *_* | protein of unknown function |
| *llo1183* | *_* | protein of unknown function |
| *llo1184* | *_* | protein of unknown function |
| *llo1190* | *_* | conserved protein of unknown function |
| *llo1191* | *_* | protein of unknown function |
| *llo1192* | *_* | protein of unknown function |
| *llo1193* | *_* | protein of unknown function |
| *llo1194* | *_* | Nitroreductase |
| *llo1196* | *_* | protein of unknown function |
| *llo1197* | *_* | Adenylate/Guanylate Cyclase |
| *llo1198* | *_* | protein of unknown function |
| *llo1217* | *_* | protein of unknown function |
| *llo1218* | *_* | protein of unknown function |
| *llo4025* | *_* | protein of unknown function |
| *llo1242* | *_* | conserved exported protein of unknown function |
| *llo1243* | *_* | exported protein of unknown function |
| *llo1244* | *_* | membrane protein of unknown function |
| *llo1252* | *_* | conserved protein of unknown function |
| *llo1256* | *mdtA* | multidrug efflux system, subunit A |
| *llo1257* | *mdtB* | multidrug efflux system, subunit B |
| *llo1258* | *mdtC* | multidrug efflux system, subunit C |
| *llo1260* | *_* | protein of unknown function |
| *llo1261* | *_* | protein of unknown function |
| *llo1262* | *_* | putative Riboflavin synthase |
| *llo1263* | *_* | protein of unknown function |
| *llo1264* | *_* | conserved protein of unknown function |
| *llo1265* | *_* | protein of unknown function |
| *llo1267* | *_* | protein of unknown function |
| *llo1268* | *_* | protein of unknown function |
| *llo1269* | *_* | protein of unknown function |
| *llo1270* | *_* | protein of unknown function |
| *llo1289* | *_* | exported protein of unknown function |
| *llo1290* | *_* | protein of unknown function |
| *llo1292* | *_* | protein of unknown function |
| *llo4026* | *_* | protein of unknown function |
| *llo1306* | *_* | protein of unknown function |
| *llo1307* | *_* | protein of unknown function |
| *llo1308* | *_* | protein of unknown function |
| *llo1312* | *_* | exported protein of unknown function |
| *llo1314* | *_* | protein of unknown function |
| *llo1320* | *DWF* | 7-dehydrocholesterol reductase (7-DHC reductase) (Sterol Delta(7)-reductase) |
| *llo4027* | *_* | protein of unknown function |
| *llo1330* | *_* | protein of unknown function |
| *llo1331* | *_* | protein of unknown function |
| *llo1332* | *_* | protein of unknown function |
| *llo1336* | *_* | protein of unknown function |
| *llo1337* | *_* | protein of unknown function |
| *llo1339* | *_* | protein of unknown function |
| *llo1340* | *_* | protein of unknown function |
| *llo1352* | *_* | membrane protein of unknown function |
| *llo1353* | *_* | membrane protein of unknown function |
| *llo1354* | *_* | Putative endo-beta-galactosidase (fragment) |
| *llo1355* | *katA* | Catalase |
| *llo1357* | *poxB* | pyruvate dehydrogenase (pyruvate oxidase), thiamin-dependent, FAD-binding |
| *llo1358* | *_* | protein of unknown function |
| *llo1359* | *_* | exported protein of unknown function |
| *llo1361* | *_* | protein of unknown function |
| *llo1362* | *_* | protein of unknown function |
| *llo1364* | *_* | protein of unknown function |
| *llo1369* | *_* | protein of unknown function |
| *llo1370* | *_* | membrane protein of unknown function |
| *llo1371* | *_* | protein of unknown function |
| *llo1373* | *_* | protein of unknown function |
| *llo1374* | *_* | protein of unknown function |
| *llo1375* | *_* | putative NAD(+) synthase |
| *llo1376a* | *_* | Sensor protein (fragment) |
| *llo1376b* | *_* | Sensor protein (fragment) |
| *llo1376c* | *_* | Sensor protein (fragment) |
| *llo1381* | *_* | exported protein of unknown function |
| *llo1383* | *_* | protein of unknown function |
| *llo1385* | *_* | Alternative oxidase |
| *llo1390* | *_* | protein of unknown function |
| *llo1392* | *_* | protein of unknown function |
| *llo1393* | *_* | protein of unknown function |
| *llo1395* | *_* | membrane protein of unknown function |
| *llo1396* | *_* | protein of unknown function |
| *llo4028* | *_* | protein of unknown function |
| *llo1398* | *_* | Aminotransferase, class V superfamily (fragment) |
| *llo1401* | *_* | conserved protein of unknown function |
| *llo1402* | *_* | protein of unknown function |
| *llo1403* | *_* | protein of unknown function |
| *llo1404* | *_* | protein of unknown function |
| *llo1405* | *_* | exported protein of unknown function |
| *llo1406* | *_* | exported protein of unknown function |
| *llo1407* | *tam* | trans-aconitate methyltransferase |
| *llo1409* | *_* | membrane protein of unknown function |
| *llo1410* | *_* | putative Polygalacturonase |
| *llo1411* | *_* | protein of unknown function |
| *llo1412* | *_* | protein of unknown function |
| *llo1413* | *_* | protein of unknown function |
| *llo1415* | *_* | protein of unknown function |
| *llo1417* | *_* | conserved protein of unknown function |
| *llo1421* | *_* | putative Delta(12)-fatty acid dehydrogenase |
| *llo1422* | *_* | putative Fumarate and nitrate reduction regulatory protein |
| *llo1423* | *_* | protein of unknown function |
| *llo1424* | *_* | protein of unknown function |
| *llo1426* | *_* | protein of unknown function |
| *llo1427* | *_* | protein of unknown function, F-box domain |
| *llo1428* | *_* | protein of unknown function |
| *llo1429* | *_* | Insertion element iso-IS1n protein insB (fragment) |
| *llo4029* | *_* | protein of unknown function |
| *llo1432* | *_* | protein of unknown function |
| *llo1433* | *_* | protein of unknown function |
| *llo1435* | *_* | exported protein of unknown function |
| *llo1436* | *_* | protein of unknown function |
| *llo1438* | *_* | exported protein of unknown function |
| *llo1454* | *_* | protein of unknown function |
| *llo1456* | *_* | protein of unknown function |
| *llo1457* | *_* | protein of unknown function |
| *llo4030* | *_* | protein of unknown function |
| *llo1458* | *_* | protein of unknown function |
| *llo1473* | *_* | protein of unknown function |
| *llo1506* | *_* | protein of unknown function |
| *llo1515* | *_* | protein of unknown function |
| *llo1516* | *_* | exported protein of unknown function |
| *llo1525* | *_* | protein of unknown function |
| *llo1536* | *_* | protein of unknown function |
| *llo1537* | *_* | protein of unknown function |
| *llo1538* | *sunT* | ABC-type bacteriocin/lantibiotic exporters |
| *llo1540* | *cpo* | Similar to chloroperoxidase |
| *llo1542* | *_* | protein of unknown function |
| *llo1543* | *_* | protein of unknown function |
| *llo1544* | *_* | protein of unknown function |
| *llo1545* | *_* | protein of unknown function |
| *llo1558* | *_* | exported protein of unknown function |
| *llo1562* | *_* | Sugar-phosphate isomerases, RpiB/LacA/LacB family |
| *llo1565* | *_* | exported protein of unknown function |
| *llo1569* | *_* | protein of unknown function |
| *llo1575* | *_* | protein of unknown function |
| *llo4031* | *_* | protein of unknown function |
| *llo1577* | *_* | protein of unknown function |
| *llo1578* | *_* | protein of unknown function |
| *llo1586* | *_* | putative hydolase |
| *llo1591* | *_* | protein of unknown function |
| *llo1594* | *_* | protein of unknown function |
| *llo1596* | *_* | exported protein of unknown function |
| *llo1599* | *_* | Glyoxalase/bleomycin resistance protein/dioxygenase |
| *llo1600* | *_* | membrane protein of unknown function |
| *llo1604* | *_* | protein of unknown function |
| *llo4034* | *_* | protein of unknown function |
| *llo1607* | *_* | Naringenin-chalcone synthase |
| *llo1608* | *_* | Methyltransferase type 11 |
| *llo1609* | *_* | FAD dependent oxidoreductase |
| *llo1610* | *_* | conserved protein of unknown function |
| *llo1612* | *_* | putative Stress responsive alpha-beta barrel domain protein |
| *llo4035* | *_* | protein of unknown function |
| *llo1613* | *_* | membrane protein of unknown function |
| *llo1614* | *_* | Glycine hydroxymethyltransferase (modular protein) |
| *llo1615* | *arpA* | ArpA protein |
| *llo1618* | *_* | protein of unknown function |
| *llo1619* | *_* | protein of unknown function |
| *llo1621* | *_* | conserved protein of unknown function |
| *llo1622* | *_* | protein of unknown function |
| *llo1623* | *_* | protein of unknown function |
| *llo1624* | *_* | protein of unknown function |
| *llo1626* | *_* | protein of unknown function |
| *llo1627* | *_* | conserved protein of unknown function |
| *llo1628* | *_* | protein of unknown function |
| *llo1629* | *_* | protein of unknown function |
| *llo1630* | *_* | transposase |
| *llo1631* | *_* | protein of unknown function |
| *llo1632* | *_* | protein of unknown function |
| *llo1635* | *_* | exported protein of unknown function |
| *llo1636* | *_* | conserved protein of unknown function |
| *llo1637* | *_* | protein of unknown function |
| *llo1638* | *_* | protein of unknown function |
| *llo1639* | *_* | protein of unknown function |
| *llo1640* | *_* | protein of unknown function |
| *llo1641* | *_* | exported protein of unknown function |
| *llo1642* | *_* | protein of unknown function |
| *llo1643* | *_* | protein of unknown function |
| *llo1644* | *_* | protein of unknown function |
| *llo1646* | *_* | protein of unknown function |
| *llo1647* | *_* | protein of unknown function |
| *llo1648* | *_* | conserved protein of unknown function |
| *llo1651* | *_* | protein of unknown function |
| *llo1652* | *_* | protein of unknown function |
| *llo4036* | *_* | protein of unknown function |
| *llo1656* | *_* | protein of unknown function |
| *llo1659* | *_* | protein of unknown function |
| *llo1660* | *_* | protein of unknown function |
| *llo1661* | *_* | protein of unknown function |
| *llo1663* | *_* | conserved exported protein of unknown function |
| *llo1666* | *_* | membrane protein of unknown function |
| *llo1667* | *_* | Asp-tRNAAsn/Glu-tRNAGln amidotransferase A subunit |
| *llo1670* | *_* | exported protein of unknown function |
| *llo1674* | *_* | transposase |
| *llo1676a* | *_* | protein of unknown function |
| *llo1676b* | *_* | conserved protein of unknown function |
| *llo1678* | *_* | protein of unknown function |
| *llo1679* | *_* | protein of unknown function |
| *llo1680* | *_* | protein of unknown function |
| *llo1681* | *_* | protein of unknown function |
| *llo1684* | *_* | putative Drug resistance transporter |
| *llo1685* | *_* | protein of unknown function |
| *llo1693* | *_* | protein of unknown function |
| *llo4037* | *_* | exported protein of unknown function |
| *llo1706* | *_* | protein of unknown function |
| *llo1713* | *_* | protein of unknown function |
| *llo1715* | *_* | protein of unknown function |
| *llo1716* | *_* | protein of unknown function |
| *llo1721* | *_* | protein of unknown function |
| *llo1726* | *_* | Putative transposase, truncated |
| *llo1728* | *_* | protein of unknown function |
| *llo1737* | *_* | protein of unknown function |
| *llo1742* | *_* | protein of unknown function |
| *llo1743* | *_* | protein of unknown function |
| *llo1792* | *_* | protein of unknown function |
| *llo1793* | *_* | protein of unknown function |
| *llo1818* | *_* | protein of unknown function |
| *llo1822* | *_* | protein of unknown function |
| *llo1824* | *_* | protein of unknown function |
| *llo1825* | *_* | protein of unknown function |
| *llo1826* | *_* | exported protein of unknown function |
| *llo1827* | *_* | membrane protein of unknown function |
| *llo4038* | *_* | protein of unknown function |
| *llo1828* | *_* | protein of unknown function |
| *llo1829* | *_* | membrane protein of unknown function |
| *llo1833* | *_* | conserved membrane protein of unknown function |
| *llo1834* | *traG* | mating contact stabilization protein TraG |
| *llo1836* | *trbB* | Putative type-F conjugative transfer system pilin assembly thiol-disulfide isomerase |
| *llo1837* | *traF* | Putative Type-F conjugative transfer system pilin assembly protein |
| *llo1838* | *traN* | putative Type-F conjugative transfer system mating-pair stabilization protein |
| *llo1842* | *trbI* | F pilus extension/retraction protein TrbI Inner membrane protein |
| *llo1843* | *traC* | F pilus assembly protein TraC |
| *llo1844* | *traV* | Putative F pilus assembly protein |
| *llo1845* | *traB* | putative F pilus assembly protein (traB) |
| *llo1846* | *traK* | putative Type-F conjugative transfer system secretin |
| *llo1847* | *traE* | Type IV conjugative transfer system protein TraE |
| *llo1848* | *traL* | Type IV conjugative transfer system protein TraL, proteobacteria |
| *llo1849* | *traA* | Putative type IV conjugative transfer system pilin TraA |
| *llo1854* | *_* | protein of unknown function |
| *llo1855* | *_* | protein of unknown function |
| *llo1856* | *_* | protein of unknown function |
| *llo1857* | *_* | protein of unknown function |
| *llo1858* | *_* | TobX protein (fragment) |
| *llo1859* | *_* | DEAD_2 domain protein |
| *llo1860* | *_* | protein of unknown function |
| *llo1865* | *_* | conserved protein of unknown function |
| *llo1866* | *_* | protein of unknown function |
| *llo1867* | *_* | conserved membrane protein of unknown function |
| *llo1868* | *_* | membrane protein of unknown function |
| *llo1869* | *_* | conserved protein of unknown function |
| *llo1870* | *_* | membrane protein of unknown function |
| *llo1871* | *_* | protein of unknown function |
| *llo1872* | *_* | protein of unknown function |
| *llo1873* | *_* | putative RNA-directed DNA polymerase (Reverse transcriptase) |
| *llo1874* | *_* | protein of unknown function |
| *llo1877* | *_* | protein of unknown function |
| *llo1878* | *_* | Asparagine synthase, glutamine-hydrolyzing |
| *llo1882* | *_* | protein of unknown function |
| *llo1884* | *_* | conserved protein of unknown function |
| *llo1885* | *_* | conserved membrane protein of unknown function |
| *llo1887* | *_* | protein of unknown function |
| *llo1888* | *_* | exported protein of unknown function |
| *llo4039* | *_* | Putative integrase protein (fragment) |
| *llo1889* | *_* | protein of unknown function |
| *llo1890* | *_* | protein of unknown function |
| *llo4040* | *_* | protein of unknown function |
| *llo1892* | *_* | exported protein of unknown function |
| *llo1893* | *_* | Prolyl endopeptidase (Post-proline cleaving enzyme) (PE) |
| *llo1896* | *_* | protein of unknown function |
| *llo1897* | *_* | membrane protein of unknown function |
| *llo1898* | *_* | protein of unknown function |
| *llo1899* | *_* | protein of unknown function |
| *llo1900* | *_* | protein of unknown function |
| *llo1902* | *_* | protein of unknown function |
| *llo1903* | *_* | membrane protein of unknown function |
| *llo1904* | *_* | protein of unknown function |
| *llo1906b* | *_* | protein of unknown function |
| *llo1907* | *_* | exported protein of unknown function |
| *llo1909* | *_* | conserved protein of unknown function |
| *llo1910* | *_* | transposase |
| *llo1911* | *_* | transposase |
| *llo1913* | *_* | RES domain protein |
| *llo1914* | *_* | conserved protein of unknown function |
| *llo1915* | *_* | protein of unknown function |
| *llo1916* | *_* | protein of unknown function |
| *llo1917* | *_* | protein of unknown function |
| *llo1918* | *_* | protein of unknown function |
| *llo1919* | *_* | protein of unknown function |
| *llo1920* | *_* | protein of unknown function |
| *llo1921* | *_* | protein of unknown function |
| *llo1922* | *_* | protein of unknown function |
| *llo1923* | *_* | protein of unknown function |
| *llo1925* | *yciI* | putative enzyme |
| *llo1926* | *_* | conserved protein of unknown function |
| *llo1927* | *_* | protein of unknown function |
| *llo1928* | *_* | membrane protein of unknown function |
| *llo1929* | *_* | protein of unknown function |
| *llo4041* | *_* | protein of unknown function |
| *llo1937* | *_* | Pentapeptide repeat protein |
| *llo1938* | *_* | protein of unknown function |
| *llo1939* | *_* | protein of unknown function |
| *llo1940* | *_* | protein of unknown function |
| *llo1941* | *_* | protein of unknown function |
| *llo1954* | *_* | protein of unknown function |
| *llo1957* | *_* | protein of unknown function |
| *llo1975* | *zupT* | putative dioxygenase |
| *llo1976* | *_* | protein of unknown function |
| *llo1977* | *_* | protein of unknown function |
| *llo1978* | *_* | exported protein of unknown function |
| *llo4042* | *_* | protein of unknown function |
| *llo1980* | *_* | protein of unknown function |
| *llo1982* | *_* | putative Peptidyl-dipeptidase A |
| *llo1983* | *_* | protein of unknown function |
| *llo1984* | *_* | protein of unknown function |
| *llo1989* | *_* | protein of unknown function |
| *llo1990* | *_* | exported protein of unknown function |
| *llo1993* | *_* | putative Cysteine desulfurase |
| *llo1996* | *_* | protein of unknown function |
| *llo1997* | *_* | protein of unknown function |
| *llo1999* | *_* | putative Sphingomyelin phosphodiesterase |
| *llo2001* | *_* | NAD-dependent epimerase/dehydratase precursor |
| *llo2004* | *_* | protein of unknown function |
| *llo2005* | *_* | protein of unknown function |
| *llo2006* | *_* | protein of unknown function |
| *llo2007* | *_* | protein of unknown function |
| *llo2008* | *_* | exported protein of unknown function |
| *llo2009* | *_* | protein of unknown function |
| *llo2010* | *_* | protein of unknown function |
| *llo4044* | *_* | protein of unknown function |
| *llo2031* | *_* | protein of unknown function |
| *llo2060* | *_* | protein of unknown function |
| *llo2061* | *_* | protein of unknown function |
| *llo2062* | *_* | exported protein of unknown function |
| *llo2066* | *_* | protein of unknown function |
| *llo2092* | *_* | protein of unknown function |
| *llo4046* | *_* | transposase (fragment) |
| *llo2095* | *_* | conserved protein of unknown function |
| *llo2096* | *_* | protein of unknown function |
| *llo2097* | *_* | putative transcriptional regulator |
| *llo2098* | *_* | conserved protein of unknown function |
| *llo2099b* | *_* | Putative kinase (fragment) |
| *llo2099a* | *_* | protein of unknown function |
| *llo2100* | *_* | protein of unknown function |
| *llo2102* | *_* | conserved membrane protein of unknown function |
| *llo2103* | *_* | protein of unknown function |
| *llo2105* | *_* | protein of unknown function |
| *llo2106b* | *_* | protein of unknown function |
| *llo2107* | *_* | protein of unknown function |
| *llo2110* | *_* | protein of unknown function |
| *llo2111* | *_* | conserved exported protein of unknown function |
| *llo2112* | *_* | conserved protein of unknown function |
| *llo2114* | *_* | membrane protein of unknown function |
| *llo2117* | *_* | Fatty-acid desaturase |
| *llo2131* | *_* | protein of unknown function |
| *llo2132* | *_* | protein of unknown function |
| *llo2133a* | *_* | protein of unknown function |
| *llo2133b* | *_* | protein of unknown function |
| *llo2134* | *_* | protein of unknown function |
| *llo2135* | *_* | protein of unknown function |
| *llo4047* | *_* | protein of unknown function |
| *llo2136* | *_* | protein of unknown function |
| *llo2137* | *_* | protein of unknown function |
| *llo2138* | *_* | protein of unknown function |
| *llo2139* | *_* | Transcriptional regulator |
| *llo2140* | *_* | conserved protein of unknown function |
| *llo2145* | *_* | protein of unknown function |
| *llo2146* | *_* | protein of unknown function |
| *llo4048* | *_* | protein of unknown function |
| *llo4049* | *_* | Protein of unknown function |
| *llo2152* | *_* | membrane protein of unknown function |
| *llo2153* | *_* | protein of unknown function |
| *llo2154* | *_* | protein of unknown function |
| *llo4050* | *_* | Cation-transporting ATPase (fragment) |
| *llo4051* | *_* | Cation-transporting P-tyep ATPase (fragment) |
| *llo2156* | *_* | protein of unknown function |
| *llo2159* | *_* | protein of unknown function |
| *llo2161* | *_* | exported protein of unknown function |
| *llo2162* | *_* | putative Alanine racemase |
| *llo2164* | *_* | exported protein of unknown function |
| *llo4052* | *_* | protein of unknown function |
| *llo2165* | *_* | protein of unknown function |
| *llo2167* | *_* | membrane protein of unknown function |
| *llo2168* | *_* | protein of unknown function |
| *llo2169* | *_* | putative Zeaxanthin epoxidase |
| *llo4055* | *_* | protein of unknown function |
| *llo2173* | *_* | protein of unknown function |
| *llo2174* | *_* | Alanine--glyoxylate aminotransferase 2 homolog 3, mitochondrial precursor |
| *llo4056* | *_* | protein of unknown function |
| *llo2175* | *_* | protein of unknown function |
| *llo2176* | *_* | protein of unknown function |
| *llo2177* | *_* | protein of unknown function |
| *llo2178* | *_* | protein of unknown function |
| *llo2179* | *_* | protein of unknown function |
| *llo2180* | *_* | protein of unknown function |
| *llo4057* | *_* | protein of unknown function |
| *llo2185* | *_* | protein of unknown function |
| *llo2186* | *_* | membrane protein of unknown function |
| *llo4058* | *_* | protein of unknown function |
| *llo2187* | *_* | protein of unknown function |
| *llo2190* | *_* | exported protein of unknown function |
| *llo2191a* | *_* | protein of unknown function |
| *llo2191b* | *_* | conserved protein of unknown function |
| *llo2192* | *_* | transposase (fragment) |
| *llo2193* | *_* | protein of unknown function |
| *llo2200* | *_* | protein of unknown function |
| *llo2202* | *_* | protein of unknown function |
| *llo2203* | *_* | protein of unknown function |
| *llo2204* | *_* | protein of unknown function |
| *llo2205* | *_* | protein of unknown function |
| *llo2206* | *_* | protein of unknown function |
| *llo2208* | *_* | Chromate transporter, chromate ion transporter (CHR) family |
| *llo2209* | *_* | protein of unknown function |
| *llo2219* | *_* | conserved protein of unknown function |
| *llo2221* | *_* | exported protein of unknown function |
| *llo2222* | *_* | protein of unknown function |
| *llo2226* | *_* | conserved protein of unknown function |
| *llo2228b* | *_* | L-lactate dehydrogenase fragment |
| *llo2228a* | *_* | L-lactate dehydrogenase FMN linked (fragment) |
| *llo2229* | *_* | putative Amidohydrolase 2 |
| *llo2230* | *_* | Putative calcium binding transcriptional regulatory protein (fragment) |
| *llo2237* | *_* | protein of unknown function |
| *llo2240* | *_* | protein of unknown function |
| *llo2242* | *_* | Sulfatase |
| *llo2244* | *_* | exported protein of unknown function |
| *llo2247* | *_* | conserved membrane protein of unknown function |
| *llo2248* | *_* | protein of unknown function |
| *llo2249* | *_* | protein of unknown function |
| *llo2255* | *_* | protein of unknown function |
| *llo2257* | *_* | putative prolin-rich exported protein |
| *llo4060* | *_* | protein of unknown function |
| *llo2269* | *_* | protein of unknown function |
| *llo2270* | *_* | conserved protein of unknown function |
| *llo2271* | *dehII* | Haloacid dehalogenase, type II |
| *llo2272* | *_* | membrane protein of unknown function |
| *llo2273* | *_* | protein of unknown function |
| *llo2274* | *_* | protein of unknown function |
| *llo2275* | *lys* | Saccharopine dehydrogenase |
| *llo2282a* | *_* | conserved protein of unknown function |
| *llo2282b* | *_* | protein of unknown function |
| *llo2282c* | *_* | conserved protein of unknown function |
| *llo2285* | *_* | protein of unknown function |
| *llo2288* | *_* | RND efflux system, outer membrane lipoprotein, NodT family precursor |
| *llo2289b* | *_* | Estradiol 17-beta-dehydrogenase 2 (fragment) |
| *llo2289c* | *_* | protein of unknown function |
| *llo4061* | *_* | protein of unknown function |
| *llo2291* | *_* | Fatty-acid desaturase |
| *llo2292* | *_* | protein of unknown function |
| *llo2293* | *_* | protein of unknown function |
| *llo2294* | *_* | protein of unknown function |
| *llo2298* | *_* | protein of unknown function |
| *llo2299* | *_* | exported protein of unknown function |
| *llo2300* | *_* | protein of unknown function |
| *llo2303* | *ndh* | respiratory NADH dehydrogenase 2/cupric reductase |
| *llo2304* | *_* | conserved protein of unknown function |
| *llo2313* | *_* | protein of unknown function |
| *llo2327* | *_* | protein of unknown function |
| *llo2329* | *_* | GTP-binding protein ypt1 (modular protein) |
| *llo2350a* | *_* | protein of unknown function |
| *llo2352* | *_* | protein of unknown function |
| *llo2353a* | *_* | protein of unknown function |
| *llo2353b* | *_* | protein of unknown function |
| *llo2354* | *mutT* | Mutator MutT protein |
| *llo2355* | *_* | exported protein of unknown function |
| *llo2358* | *_* | putative Pyridoxal-dependent decarboxylase family protein |
| *llo2359* | *_* | Putative chitin/cellulose binding protein (fragment) |
| *llo4063* | *_* | protein of unknown function |
| *llo2366* | *_* | conserved protein of unknown function |
| *llo2367* | *_* | putative Phosphoglycolate phosphatase |
| *llo2369* | *_* | exported protein of unknown function |
| *llo2371* | *_* | protein of unknown function |
| *llo2372* | *_* | putative Opine dehydrogenase |
| *llo2373* | *_* | exported protein of unknown function |
| *llo2374* | *_* | protein of unknown function |
| *llo2376* | *_* | membrane protein of unknown function |
| *llo2377* | *_* | membrane protein of unknown function |
| *llo2378* | *fhuC* | Ferrichrome transport ATP-binding protein fhuC |
| *llo2380* | *_* | protein of unknown function |
| *llo2381* | *_* | membrane protein of unknown function |
| *llo2384* | *_* | exported protein of unknown function |
| *llo2385* | *_* | membrane protein of unknown function |
| *llo4064* | *_* | protein of unknown function |
| *llo2391* | *_* | membrane protein of unknown function |
| *llo2394* | *_* | protein of unknown function |
| *llo4065* | *_* | protein of unknown function |
| *llo2399* | *_* | protein of unknown function |
| *llo2401* | *_* | exported protein of unknown function |
| *llo2403* | *_* | protein of unknown function |
| *llo2405* | *_* | protein of unknown function |
| *llo2406* | *_* | protein of unknown function |
| *llo2407* | *_* | protein of unknown function |
| *llo2416* | *_* | Polyhydroxyalkanoate depolymerase, intracellular |
| *llo2417* | *_* | protein of unknown function |
| *llo2419* | *_* | protein of unknown function |
| *llo2420* | *_* | protein of unknown function |
| *llo2421* | *_* | protein of unknown function |
| *llo2422* | *_* | protein of unknown function |
| *llo2423* | *_* | protein of unknown function |
| *llo2424* | *_* | protein of unknown function |
| *llo2425* | *_* | protein of unknown function |
| *llo2426* | *_* | protein of unknown function |
| *llo2427* | *_* | Universal stress protein, UspA family |
| *llo2429* | *_* | protein of unknown function |
| *llo2430* | *_* | protein of unknown function |
| *llo2432* | *_* | protein of unknown function |
| *llo2435* | *_* | membrane protein of unknown function |
| *llo2437* | *_* | putative acyl-CoA dehydrogenase |
| *llo4066* | *_* | exported protein of unknown function |
| *llo2440* | *_* | protein of unknown function |
| *llo2455* | *_* | putative LicD family protein |
| *llo2456* | *_* | protein of unknown function |
| *llo2457* | *_* | protein of unknown function |
| *llo2458* | *_* | protein of unknown function |
| *llo2459* | *_* | protein of unknown function |
| *llo2460* | *_* | protein of unknown function |
| *llo2467* | *_* | membrane protein of unknown function |
| *llo2468* | *kdpA* | potassium translocating ATPase, subunit A |
| *llo2469* | *kdpB* | potassium translocating ATPase, subunit B |
| *llo2470* | *kdpC* | potassium translocating ATPase, subunit C |
| *llo2471* | *_* | Sensor protein (fragment) |
| *llo2476* | *_* | protein of unknown function |
| *llo2479* | *frmC* | S-formylglutathione hydrolase |
| *llo2480* | *frmA* | alcohol dehydrogenase class III/glutathione-dependent formaldehyde dehydrogenase |
| *llo2482* | *_* | protein of unknown function |
| *llo2483* | *_* | protein of unknown function |
| *llo2484* | *_* | protein of unknown function |
| *llo2485* | *_* | putative Serine-type D-Ala-D-Ala carboxypeptidase |
| *llo2493* | *_* | conserved protein of unknown function |
| *llo2494* | *_* | protein of unknown function |
| *llo2498* | *_* | exported protein of unknown function |
| *llo2509a* | *_* | Insertion element iso-IS1n protein insB (fragment) |
| *llo2509b* | *_* | Insertion element iso-IS1n protein insB (fragment) |
| *llo2511* | *_* | protein of unknown function |
| *llo2513* | *_* | protein of unknown function |
| *llo2521* | *FCA* | Cytosine deaminase (Cytosine aminohydrolase) |
| *llo2522* | *_* | protein of unknown function |
| *llo2535* | *iaaA* | isoaspartyl dipeptidase with L-asparaginase activity |
| *llo2536* | *_* | Cyanophycinase |
| *llo2537* | *cphA* | Cyanophycin synthetase |
| *llo2538* | *_* | PRC-barrel domain protein (fragment) |
| *llo2539* | *yjbJ* | putative stress response protein |
| *llo2540* | *_* | conserved protein of unknown function |
| *llo2541* | *_* | membrane protein of unknown function |
| *llo2542* | *_* | putative Cyclic nucleotide-binding protein |
| *llo2548* | *_* | protein of unknown function |
| *llo2552* | *_* | putative Purine nucleosidase |
| *llo2555* | *_* | protein of unknown function |
| *llo2556* | *_* | protein of unknown function |
| *llo2558* | *_* | protein of unknown function |
| *llo4068* | *_* | protein of unknown function |
| *llo4069* | *_* | protein of unknown function |
| *llo2590* | *_* | putative MscS Mechanosensitive ion channel |
| *llo2592* | *_* | Globin (fragment) |
| *llo2595* | *_* | protein of unknown function |
| *llo4071* | *_* | protein of unknown function |
| *llo2597* | *_* | protein of unknown function |
| *llo2598* | *_* | protein of unknown function |
| *llo2619* | *_* | protein of unknown function |
| *llo4072* | *_* | exported protein of unknown function |
| *llo2627* | *_* | protein of unknown function |
| *llo2628* | *_* | protein of unknown function |
| *llo2631* | *_* | membrane protein of unknown function |
| *llo4073* | *_* | protein of unknown function |
| *llo2637* | *_* | protein of unknown function |
| *llo2643* | *_* | protein of unknown function |
| *llo2644* | *_* | protein of unknown function |
| *llo2646* | *_* | protein of unknown function |
| *llo2648* | *_* | protein of unknown function |
| *llo2655* | *_* | protein of unknown function |
| *llo2656* | *_* | transposase |
| *llo2657* | *_* | transposase |
| *llo2668* | *_* | protein of unknown function |
| *llo2669* | *_* | protein of unknown function |
| *llo2671* | *_* | protein of unknown function |
| *llo2672a* | *_* | conserved protein of unknown function |
| *llo2672b* | *_* | protein of unknown function |
| *llo2673* | *_* | conserved protein of unknown function |
| *llo2676* | *_* | protein of unknown function |
| *llo2677* | *_* | RNA binding S1 |
| *llo2678* | *chaB* | putative conserved cation transport regulator |
| *llo2682* | *_* | protein of unknown function |
| *llo2698* | *_* | exported protein of unknown function |
| *llo2711* | *_* | protein of unknown function |
| *llo2737* | *_* | membrane protein of unknown function |
| *llo2746* | *_* | protein of unknown function |
| *llo4075* | *_* | protein of unknown function |
| *llo2765* | *_* | protein of unknown function |
| *llo2767* | *_* | Phage shock protein C, PspC |
| *llo2768* | *_* | protein of unknown function |
| *llo2769* | *_* | protein of unknown function |
| *llo4076* | *_* | protein of unknown function |
| *llo2773* | *_* | protein of unknown function |
| *llo2774* | *_* | protein of unknown function |
| *llo2775* | *_* | protein of unknown function |
| *llo2776* | *_* | protein of unknown function |
| *llo2777* | *_* | exported protein of unknown function |
| *llo2779* | *_* | putative Histidine kinase |
| *llo2793* | *ligB* | component of the Dot/Icm secretion system |
| *llo2809* | *_* | protein of unknown function |
| *llo4077* | *_* | protein of unknown function |
| *llo4078* | *_* | protein of unknown function |
| *llo2823* | *_* | protein of unknown function |
| *llo2825* | *_* | putative peroxisomal (S)-2-hydroxy-acid oxidase 2 |
| *llo2826* | *_* | protein of unknown function |
| *llo2828* | *_* | protein of unknown function |
| *llo4079* | *_* | protein of unknown function |
| *llo2833* | *_* | protein of unknown function |
| *llo2834* | *_* | protein of unknown function |
| *llo2835* | *_* | protein of unknown function |
| *llo2836* | *_* | protein of unknown function |
| *llo2837* | *_* | protein of unknown function |
| *llo2838* | *_* | protein of unknown function |
| *llo2847* | *_* | protein of unknown function |
| *llo2849* | *_* | conserved membrane protein of unknown function |
| *llo4080* | *_* | protein of unknown function |
| *llo4082* | *_* | protein of unknown function |
| *llo2864* | *_* | Prophage CP4-57 regulatory protein (AlpA) (fragment) |
| *llo2866* | *_* | putative Type I restriction enzyme, S subunit |
| *llo2868* | *_* | protein of unknown function |
| *llo2869* | *_* | Metallophosphoesterase |
| *llo2870* | *_* | protein of unknown function |
| *llo2876* | *_* | transposase |
| *llo2891* | *_* | exported protein of unknown function |
| *llo2897* | *_* | conserved protein of unknown function |
| *llo2898* | *_* | conserved protein of unknown function |
| *llo2900* | *_* | putative toxin secretion ABC transporter, ATP-binding subunit |
| *llo2901* | *_* | exported protein of unknown function |
| *llo2902* | *_* | protein of unknown function |
| *llo2903* | *_* | protein of unknown function |
| *llo2904* | *_* | protein of unknown function |
| *llo2905* | *_* | transposase |
| *llo2906* | *_* | membrane protein of unknown function |
| *llo2907* | *_* | protein of unknown function |
| *llo2908* | *_* | protein of unknown function |
| *llo2909* | *_* | protein of unknown function |
| *llo2910* | *_* | exported protein of unknown function |
| *llo2911* | *_* | transposase |
| *llo2912* | *_* | protein of unknown function |
| *llo2913* | *_* | protein of unknown function |
| *llo2914* | *_* | protein of unknown function |
| *llo2915* | *_* | membrane protein of unknown function |
| *llo2916* | *_* | protein of unknown function |
| *llo4083* | *_* | protein of unknown function |
| *llo2922* | *_* | Oxidoreductase FAD/NAD(P)-binding |
| *llo2923* | *_* | conserved protein of unknown function |
| *llo2924* | *_* | conserved protein of unknown function |
| *llo2925* | *_* | protein of unknown function |
| *llo2931* | *zupT* | putative dioxygenase |
| *llo2932b* | *_* | conserved protein of unknown function |
| *llo2932a* | *_* | protein of unknown function |
| *llo2935* | *_* | protein of unknown function |
| *llo2940* | *_* | protein of unknown function |
| *llo4084* | *_* | protein of unknown function |
| *llo2945* | *_* | protein of unknown function |
| *llo2946* | *_* | protein of unknown function |
| *llo2947* | *_* | conserved protein of unknown function |
| *llo2951* | *_* | protein of unknown function |
| *llo2953b* | *_* | protein of unknown function |
| *llo2957a* | *_* | protein of unknown function |
| *llo2960a* | *_* | protein of unknown function |
| *llo2961* | *_* | conserved protein of unknown function |
| *llo2962* | *_* | protein of unknown function |
| *llo2964* | *_* | protein of unknown function |
| *llo2966* | *_* | protein of unknown function |
| *llo2967* | *_* | conserved protein of unknown function |
| *llo2968* | *_* | conserved protein of unknown function |
| *llo2969* | *_* | protein of unknown function |
| *llo2970* | *_* | ABC-3 protein |
| *llo2971* | *_* | ABC transporter related |
| *llo2972* | *_* | protein of unknown function |
| *llo2973* | *_* | membrane protein of unknown function |
| *llo2977* | *_* | membrane protein of unknown function |
| *llo2979* | *_* | protein of unknown function |
| *llo2980* | *_* | protein of unknown function |
| *llo2981* | *_* | protein of unknown function |
| *llo2982* | *_* | protein of unknown function |
| *llo2984* | *_* | protein of unknown function |
| *llo2985* | *_* | protein of unknown function |
| *llo2987* | *_* | membrane protein of unknown function |
| *llo2988* | *_* | protein of unknown function |
| *llo2994* | *gadB* | glutamate decarboxylase B, PLP-dependent |
| *llo2995* | *_* | putative Multiple antibiotic resistance (MarC)-related protein |
| *llo2996* | *_* | protein of unknown function |
| *llo2998* | *_* | exported protein of unknown function |
| *llo2999* | *_* | exported protein of unknown function |
| *llo3000* | *purU* | formyltetrahydrofolate hydrolase |
| *llo3001* | *_* | protein of unknown function |
| *llo3004* | *_* | protein of unknown function |
| *llo3007* | *_* | protein of unknown function |
| *llo3008* | *_* | protein of unknown function |
| *llo3009* | *_* | protein of unknown function |
| *llo3011c* | *_* | protein of unknown function |
| *llo3011b* | *_* | Dimodular non-ribosomal peptide synthetase, thioesterase domain (fragment) |
| *llo3011a* | *_* | Putative polyketide synthase pksL (PKS) (fragment) |
| *llo3012* | *_* | transposase |
| *llo3013* | *_* | protein of unknown function |
| *llo3014* | *_* | protein of unknown function |
| *llo3015* | *_* | protein of unknown function |
| *llo3021* | *_* | exported protein of unknown function |
| *llo3022* | *_* | protein of unknown function |
| *llo3023* | *_* | protein of unknown function |
| *llo3024* | *cat* | 4-hydroxybutyrate coenzyme A transferase |
| *llo3025* | *_* | putative HpcH/HpaI aldolase/citrate lyase family protein |
| *llo3026* | *_* | MoaC domain protein |
| *llo3027* | *_* | protein of unknown function |
| *llo3031* | *_* | protein of unknown function |
| *llo3037* | *nudC* | NADH pyrophosphatase |
| *llo3038* | *_* | exported protein of unknown function |
| *llo3040* | *_* | membrane protein of unknown function |
| *llo3041* | *_* | putative o-methyltransferase |
| *llo3042* | *_* | protein of unknown function |
| *llo3044* | *dgkA* | diacylglycerol kinase |
| *llo3046* | *_* | protein of unknown function |
| *llo3047* | *_* | protein of unknown function |
| *llo3048a* | *_* | exported protein of unknown function |
| *llo3048b* | *_* | conserved protein of unknown function |
| *llo3049* | *_* | putative Calcium/calmodulin-dependent protein kinase |
| *llo3052* | *_* | conserved protein of unknown function |
| *llo3058* | *_* | membrane protein of unknown function |
| *llo3059* | *_* | protein of unknown function |
| *llo3065* | *_* | protein of unknown function |
| *llo4085* | *_* | protein of unknown function |
| *llo3077* | *_* | protein of unknown function |
| *llo3078* | *_* | protein of unknown function |
| *llo3079* | *_* | protein of unknown function |
| *llo3080* | *_* | protein of unknown function |
| *llo3081* | *_* | protein of unknown function |
| *llo3082* | *_* | protein of unknown function |
| *llo3084* | *_* | Response regulator receiver |
| *llo3085* | *_* | putative Histidine kinase |
| *llo3086* | *_* | protein of unknown function |
| *llo3090* | *_* | membrane protein of unknown function |
| *llo3093* | *_* | protein of unknown function |
| *llo3100* | *_* | protein of unknown function |
| *llo3101* | *_* | putative Beta-lactamase |
| *llo3102* | *_* | putative Pentachlorophenol monooxygenase |
| *llo3105* | *_* | protein of unknown function |
| *llo3108* | *_* | D-amino-acid dehydrogenase |
| *llo3109* | *_* | Fragment of Putative heat shock protein (N-terminal part)(part 1) |
| *llo4086* | *_* | Putative heat shock protein (part 2) |
| *llo3113* | *yjcE* | putative cation/proton antiporter |
| *llo3117* | *_* | protein of unknown function |
| *llo3119* | *_* | protein of unknown function |
| *llo3124* | *_* | Alcohol dehydrogenase, zinc-binding |
| *llo3127* | *_* | protein of unknown function |
| *llo3134* | *_* | exported protein of unknown function |
| *llo3135* | *_* | protein of unknown function |
| *llo3136* | *_* | protein of unknown function |
| *llo3137* | *_* | protein of unknown function |
| *llo3138* | *_* | exported protein of unknown function |
| *llo3139* | *budB* | putative acetolactate synthase |
| *llo3140* | *_* | Aldehyde dehydrogenase |
| *llo4087* | *_* | protein of unknown function |
| *llo3143* | *_* | exported protein of unknown function |
| *llo3146* | *_* | protein of unknown function |
| *llo3149* | *bexD* | Capsule polysaccharide export protein bexD precursor |
| *llo3151* | *ctrC* | Capsule polysaccharide export inner-membrane protein ctrC |
| *llo3152* | *ctrB* | Capsule polysaccharide export inner-membrane protein ctrB |
| *llo3153* | *_* | protein of unknown function |
| *llo3154* | *_* | protein of unknown function |
| *llo3155* | *ugd* | UDP-glucose 6-dehydrogenase |
| *llo3156* | *_* | Glycosyltransferase |
| *llo3157* | *_* | protein of unknown function |
| *llo3158* | *_* | protein of unknown function |
| *llo3159* | *_* | conserved protein of unknown function |
| *llo3160* | *_* | conserved protein of unknown function |
| *llo3161* | *_* | Glycosyl transferase, family 2 |
| *llo3162* | *_* | conserved protein of unknown function |
| *llo3163* | *_* | D-isomer specific 2-hydroxyacid dehydrogenase, NAD-binding |
| *llo3165* | *_* | conserved protein of unknown function |
| *llo3166* | *galE* | UDP-galactose-4-epimerase |
| *llo3167* | *gmd* | GDP-D-mannose dehydratase, NAD(P)-binding |
| *llo3168* | *_* | protein of unknown function |
| *llo3169* | *_* | protein of unknown function |
| *llo3170* | *_* | protein of unknown function |
| *llo3171* | *galU* | glucose-1-phosphate uridylyltransferase |
| *llo3172* | *galE* | UDP-galactose-4-epimerase |
| *llo3173* | *fcl* | bifunctional GDP-fucose synthetase |
| *llo3174* | *_* | Glycosyltransferase (fragment) |
| *llo3176* | *_* | putative Glycosyltransferase |
| *llo4088* | *_* | protein of unknown function |
| *llo4089* | *_* | protein of unknown function |
| *llo3177* | *_* | protein of unknown function |
| *llo3180* | *capI* | Protein capI |
| *llo3181* | *_* | Alpha/beta hydrolase fold precursor |
| *llo3182* | *_* | protein of unknown function |
| *llo3183* | *_* | protein of unknown function |
| *llo3184* | *_* | protein of unknown function |
| *llo3186* | *_* | exported protein of unknown function |
| *llo3188* | *_* | membrane protein of unknown function |
| *llo3191* | *_* | protein of unknown function |
| *llo3195* | *_* | Sodium/hydrogen exchanger |
| *llo3196* | *_* | protein of unknown function |
| *llo3197* | *_* | putative NAD(P)(+) transhydrogenase (AB-specific) |
| *llo3198* | *_* | protein of unknown function |
| *llo3201* | *_* | protein of unknown function |
| *llo3202* | *_* | conserved protein of unknown function |
| *llo3203* | *yhdE* | putative septum formation protein |
| *llo3204* | *_* | protein of unknown function |
| *llo3205* | *_* | protein of unknown function |
| *llo3207* | *_* | protein of unknown function |
| *llo4090* | *_* | protein of unknown function |
| *llo3210* | *_* | protein of unknown function |
| *llo3211* | *_* | Asparagine synthase, glutamine-hydrolyzing |
| *llo3212* | *_* | conserved protein of unknown function |
| *llo3213* | *_* | exported protein of unknown function |
| *llo3214* | *_* | exported protein of unknown function |
| *llo3216* | *_* | conserved protein of unknown function |
| *llo3220a* | *_* | protein of unknown function |
| *llo3222* | *_* | protein of unknown function |
| *llo3225* | *_* | conserved protein of unknown function |
| *llo3227* | *_* | protein of unknown function |
| *llo3229* | *_* | protein of unknown function |
| *llo3230* | *_* | protein of unknown function |
| *llo3231* | *_* | protein of unknown function |
| *llo3232* | *_* | protein of unknown function |
| *llo3233* | *_* | protein of unknown function |
| *llo3240* | *_* | putative Ca2+/Na+ antiporter precursor |
| *llo3242* | *_* | protein of unknown function |
| *llo3243* | *_* | protein of unknown function |
| *llo3245* | *_* | protein of unknown function |
| *llo3246* | *_* | transposase (fragment) |
| *llo3247* | *_* | transposase (fragment) |
| *llo3250* | *_* | Carboxymuconolactone decarboxylase |
| *llo3251* | *_* | transposase (fragment) |
| *llo3252* | *_* | conserved membrane protein of unknown function |
| *llo3253* | *_* | Protein containing FG-GAP repeats |
| *llo4092* | *_* | protein of unknown function |
| *llo3255* | *_* | protein of unknown function |
| *llo3256* | *_* | exported protein of unknown function |
| *llo3257* | *_* | protein of unknown function |
| *llo3258* | *_* | protein of unknown function |
| *llo4093* | *_* | conserved protein of unknown function |
| *llo3266* | *_* | protein of unknown function |
| *llo3268* | *_* | protein of unknown function |
| *llo3282* | *_* | protein of unknown function |
| *llo3283* | *_* | membrane protein of unknown function |
| *llo3287* | *_* | protein of unknown function |
| *llo3288* | *_* | protein of unknown function |
| *llo3298* | *_* | protein of unknown function |
| *llo3299* | *_* | putative Protein-glutamate O-methyltransferase |
| *llo3300* | *_* | protein of unknown function |
| *llo3301* | *_* | protein of unknown function |
| *llo3302* | *_* | putative Histidine kinase |
| *llo3303* | *cheB* | protein-glutamate methylesterase in two-component regulatory system with CheA |
| *llo3304* | *_* | Adenylate/guanylate cyclase |
| *llo3307* | *_* | conserved protein of unknown function |
| *llo3309* | *_* | exported protein of unknown function |
| *llo3334* | *_* | protein of unknown function |
| *llo4094* | *_* | protein of unknown function |
| *llo3343* | *_* | protein of unknown function |
| *llo3350* | *_* | transposase (fragment) |
| *llo3351* | *_* | protein of unknown function |
| *llo3352a* | *_* | transposase (fragment) |
| *llo3352b* | *_* | transposase (fragment) |
| *llo3353* | *_* | protein of unknown function |
| *llo3354* | *_* | protein of unknown function |
| *llo3355* | *_* | protein of unknown function |
| *llo4095* | *_* | protein of unknown function |
| *llo3357* | *_* | membrane protein of unknown function |
| *llo3359* | *_* | conserved protein of unknown function |
| *llo3361* | *_* | exported protein of unknown function |
| *llo3362* | *_* | protein of unknown function |
| *llo3363* | *_* | conserved protein of unknown function |
| *llo3364* | *_* | membrane protein of unknown function |
| *llo3373* | *_* | protein of unknown function |
| *llo3374* | *_* | protein of unknown function |
| *llo3375* | *_* | protein of unknown function |
| *llo3376* | *_* | exported protein of unknown function |
| *llo3379* | *_* | RND efflux system, outer membrane lipoprotein, NodT precursor |
| *llo3381* | *_* | putative Cation efflux system protein CzcB |
| *llo3384* | *_* | membrane protein of unknown function |
| *llo3386* | *_* | protein of unknown function |
| *llo3389* | *_* | protein of unknown function |
| *llo3390* | *_* | protein of unknown function |
| *llo3391* | *_* | putative 2-alkenal reductase |
| *llo4096* | *_* | protein of unknown function |
| *llo3394* | *_* | protein of unknown function |
| *llo3396* | *_* | membrane protein of unknown function |
| *llo3397* | *_* | exported protein of unknown function |
| *llo3408* | *_* | protein of unknown function |
| *llo3409* | *_* | protein of unknown function |
| *llo3428* | *_* | exported protein of unknown function |
| *llo3430* | *_* | protein of unknown function |
| *llo3431* | *_* | protein of unknown function |
| *llo3439* | *_* | putative Unspecific monooxygenase |
| *llo3441* | *_* | membrane protein of unknown function |
| *llo3444* | *_* | membrane protein of unknown function |
| *llo3445* | *_* | protein of unknown function |
| *llo3446* | *_* | protein of unknown function |

*All genes with less than 30% sequence identity over 80% of the length of the smallest protein
