# Supplementary material for: Analysis of the Legionella longbeachae Genome and Transcriptome Uncovers Unique Strategies to Cause Legionnaires' Disease
Source: PLoS Genet. 2010 Feb 19;6(2):e1000851. doi: 10.1371/journal.pgen.1000851 (PMC2824747; doi:10.1371/journal.pgen.1000851)
Supplement: Table S3 — Distribution of known and predicted Dot/Icm substrates of L. pneumophila in L. longbeachae. (0.31 MB DOC) [file pgen.1000851.s009.doc]

**Table S3: Distribution of known and predicted Dot/Icm substrates of *L. pneumophila* in *L. longbeachae***

| ***L. pneumophila*** | | | | ***L. longbeachae*** | | | | **Name** | **Description** |
| --- | --- | --- | --- | --- | --- | --- | --- | --- | --- |
| Philadelphia | Paris | Lens | Corby | NSW150 | ATCC39462 | 98072 | C-4E7 |  |  |
| *lpg0012* | *lpp0012* | *lpl0012* | *lpc0013* | *llo0432* | **+** | **+** | **+** | *cegC1* | Ankyrin repeat repeat |
| *lpg0038* | *lpp0037* | *lpl0038* | *lpc0039* | *-* | *-* | *-* | *-* | *ankQ/legA10* | Unknown |
| *lpg0045* | *lpp0046* | *lpl0044* | *lpc0047* | *-* | *-* | *-* | *-* |  | Unknown |
| *lpg0080* | *lpp0094* | *lpl2046* | *lpc1561* | *-* | *-* | *-* | *-* |  | Unknown |
| *lpg0081* | *lpp0095* | *-* | *-* | *-* | *-* | *-* | *-* |  | Unknown |
| *lpg0090* | *lpp0104* | *lpl0089* | *lpc0109* | *-* | *-* | *-* | *-* |  | Unknown |
| *lpg0096* | *lpp0110* | *lpl0096* | *lpc0115* | *llo1322* | **+** | **+** | **+** |  | Unknown |
| *lpg0103* | *lpp0117* | *lpl0103* | *lpc0122* | *llo3312* | **+** | **+** | **+** | *vipF* | N-terminal acetyltransferase, GNAT family |
| *lpg0126* | *lpp0140* | *lpl0125* | *lpc0146* | *-* | *-* | *-* | *-* | *cegC2* | ninein |
| *lpg0171* | *lpp0233* | *lpl0234* | *-* | *-* | *-* | *-* | *-* | *legU1* | F-box motif |
| *lpg0191* | *lpp0251* | *-* | *-* | *-* | *-* | *-* | *-* |  | Unknown |
| *lpg0227* | *lpp0286* | *lpl0281* | *lpc0303* | *llo2491* | **+** | **+** | **+** | *ceg7* | Unknown |
| *lpg0234* | *lpp0304* | *lpl0288* | *lpc0309* | *llo0425* | **+** | **+** | **+** | *sidE/laiD* | Unknown |
| *lpg0240* | *lpp0310* | *lpl0294* | *lpc0316* | *llo1601* | **+** | **+** | **+** |  | Unknown |
| *lpg0246* | *lpp0316* | *lpl0300* | *lpc0323* | *-* | *-* | *-* | *-* | *ceg9* | Unknown |
| *lpg0257* | *lpp0327* | *lpl0310* | *lpc0334* | *llo2362* | **+** | **+** | **+** | *sdeA* | Multidrug resistance protein |
| *lpg0276* | *lpp0350* | *lpl0328* | *lpc0353* | *llo0327* | **+** | **+** | **+** | *legG2* | Ras guanine nucleotide exchange factor |
| *lpg0284* | *lpp0360* | *lpl0336* | *lpc0361* | *-* | *-* | *-* | *-* | *ceg10* | Unknown |
| *lpg0285* | *lpp0361* | *lpl0337* | *lpc0362* | *-* | *-* | *-* | *-* |  | Unknown |
| *lpg0294* | *lpp0372* | *lpl0347* | *lpc0373* | *llo0464* | **+** | **+** | **+** |  | Unknown |
| *lpg0365* | *lpp0430* | *lpl0406* | *lpc2979* | *llo0525* | **+** | **+** | **+** |  | Unknown |
| *lpg0376* | *lpp0443* | *lpl0419* | *lpc2967* | *-* | *-* | *-* | *-* | *sdhA* | GRIP, coiled-coil |
| *lpg0390* | *lpp0457* | *lpl0433* | *lpc2954* | *llo2824* | **+** | **+** | **+** | *vipA* | Unknown |
| *lpg0401* | *lpp0468* | *lpl0444* | *lpc2942* | *llo2582* | **+** | **+** | **+** | *ceg11* | Unknown |
| *lpg0402* | *-* | *-* | *-* | *-* | *-* | *-* | *-* | *ankY/legA9* | Ankyrin repeat, STPK |
| *lpg0403* | *lpp0469* | *lpl0445* | *lpc2941* | *-* | *-* | *-* | *-* | *ankG/ankZ/ legA7* | Ankyrin repeat |
| *lpg0436* | *lpp0503* | *lpl0479* | *lpc2906* | *-* | *-* | *-* | *-* | *ankJ/legA11* | Ankyrin repeat |
| *lpg0437* | *lpp0504* | *lpl0480* | *lpc2905* | *-* | *-* | *-* | *-* |  | Unknown |
| *lpg0483* | *lpp0547* | *lpl0523* | *lpc2861* | *llo2705* | **+** | **+** | **+** | *ankC/legA12* | Ankyrin repeat |
| *lpg0518* | *lpp0581* | *lpl0557* | *lpc2826* | *-* | *-* | *-* | *-* |  | Unknown |
| *lpg0519* | *-* | *-* | *-* | *llo1222* | **+** | **+** | **+** |  | Unknown |
| *lpg0621* | *lpp0675* | *lpl0658* | *lpc2673* | *-* | *-* | *-* | *-* | *sidA* | Unknown |
| *lpg0634* | *lpp0688* | *lpl0671* | *lpc2660* | *llo2574* | **+** | **+** | **+** |  | Unknown |
| *lpg0642* | *lpp0696* | *lpl0679* | *lpc2651* | *-* | *-* | *-* | *-* | *wipB* | Unknown |
| *lpg0695* | *lpp0750* | *lpl0732* | *lpc2599* | *-* | *-* | *-* | *-* | *ankN/ankX legA8* | Ankyrin repeat |
| *lpg0696* | *lpp0751* | *lpl0733* | *lpc2598* | *-* | *-* | *-* | *-* |  | Unknown |
| *lpg0898* | *lpp0959* | *lpl0929* | *lpc2395* | *-* | *-* | *-* | *-* | *ceg18* | Unknown |
| *lpg0940* | *lpp1002* | *lpl0971* | *lpc2349* | *-* | *-* | *-* | *-* | *lidA* | Unknown |
| *lpg0945* | *lpp1007* | *lpl1579* | *lpc2344* | *-* | *-* | *-* | *-* | *legL1* | Leucine-rich repeats |
| *lpg0963* | *lpp1025* | *lpl0992* | *lpc2324* | *llo0934* | **+** | **+** | **+** |  | Unknown |
| *lpg1101* | *lpp1101* | *lpl1100* | *lpc2154* | *-* | *-* | *-* | *-* |  | Unknown |
| *lpg1120* | *-* | *-* | *-* | *llo2959* | **+** | **+** | **+** |  | Unknown |
| *lpg1121* | *lpp1121* | *lpl1126* | *lpc0578* | *llo1321* | **+** | **+** | **+** | *ceg19* | Unknown |
| *lpg1144* | *lpp1146* | *lpl1150* | *lpc0607* | *llo1019* | **+** | **+** | **+** | *cegC3* | Unknown |
| *lpg1145* | *lpp1147* | *lpl1151* | *lpc0608* | *-* | *-* | *-* | *-* |  | Unknown |
| *lpg1148* | *lpp1150* | *lpl1154* | *lpc0611* | *-* | *-* | *-* | *-* |  | Unknown |
| *lpg1158* | *lpp1160* | *lpl1165* | *lpc0621* | *-* | *-* | *-* | *-* |  | Unknown |
| *lpg1227* | *lpp1235* | *lpl1235* | *lpc0696* | *-* | *-* | *-* | *-* | *vpdB* | Putative Acyl transferase/acyl hydrolase |
| *lpg1273* | *lpp1236* | *lpl1236* | *lpc0698* | *-* | *-* | *-* | *-* |  | Unknown |
| *lpg1290* | *lpp1253* | *-* | *-* | *-* | *-* | *-* | *-* |  | Unknown |
| *lpg1328* | *lpp1283* | *lpl1282* | *lpc0743* | *-* | *-* | *-* | *-* | *legT* | Thaumatin domain |
| *lpg1355* | *lpp1309* | *-* | *-* | *-* | *-* | *-* | *-* | *sidG* | Coiled-coil |
| *lpg1426* | *lpp1381* | *lpl1377* | *lpc0842* | *llo1791* | **+** | **+** | **+** |  | Unknown |
| *lpg1488* | *lpp1444* | *lpl1540* | *lpc0903* | *-* | *-* | *-* | *-* | *lgt3/legc5* | Coiled-coil |
| *lpg1491* | *lpp1447* | *-* | *-* | *-* | *-* | *-* | *-* |  | Unknown |
| *lpg1496* | *lpp1453* | *lpl1530* | *lpc0915* | *-* | *-* | *-* | *-* |  | Unknown |
| *lpg1588* | *lpp1546* | *lpl1437* | *lpc1013* | *-* | *-* | *-* | *-* | *legC6* | Coiled-coil |
| *lpg1598* | *lpp1556* | *lpl1427* | *lpc1025* | *-* | *-* | *-* | *-* |  | Unknown |
| *lpg1602* | *lpp1567* | *lpl1423* | *lpc1028* | *-* | *-* | *-* | *-* | *legL2* | Leucine-rich repeats |
| *lpg1621* | *lpp1591* | *lpl1402* | *lpc1048* | *llo1014* | **+** | **+** | **+** | *ceg23* | Unknown |
| *lpg1625* | *lpp1595* | *lpl1398* | *lpc1052* | *llo0719* | **+** | **+** | **+** |  | Unknown |
| *lpg1642* | *lpp1612a/b* | *lpl1384* | *lpc1071* | *llo1144* | **+** | **+** | **+** | *sidB* | Rtx toxin, lipase |
| *lpg1660* | *lpp1631* | *lpl1625* | *lpc1090* | *-* | *-* | *-* | *-* | *legL3* | Leucine-rich repeats |
| *lpg1689* | *lpp1658* | *lpl1652* | *lpc1120* | *llo1697* | **+** | **+** | **+** |  | Unknown |
| *lpg1701* | *lpp1666* | *lpl1660* | *lpc1130* | *-* | *-* | *-* | *-* | *ppeA/legC3* | Coiled-coil |
| *lpg1702* | *lpp1667* | *lpl1661* | *lpc1131* | *-* | *-* | *-* | *-* | *ppeB* | Unknown |
| *lpg1717* | *lpp1682* | *-* | *-* | *-* | *-* | *-* | *-* |  | Unknown |
| *lpg1718* | *lpp1683* | *lpl1682* | *lpc1152* | *-* | *-* | *-* | *-* | *ankI/legAS4* | Ankyrin repeat |
| *lpg1751* | *lpp1715* | *lpl1715* | *lpc1191* | *llo2314* | **+** | **+** | **+** |  | Unknown |
| *lpg1851* | *lpp1818* | *lpl1817* | *lpc1296* | *llo1047* | **+** | **+** | **+** |  | Unknown |
| *lpg1884* | *lpp1848* | *lpl1845* | *lpc1331* | - | - | - | - | *ylfB/legC2* | Coiled-coil |
| *lpg1890* | *-* | *lpl1852* | *lpc1338* | - | - | - | - | *legLC8* | Leucine-rich repeats, coiled-coil |
| *lpg1933* | *lpp1914* | *lpl1903* | *lpc1406* | - | - | - | - |  | Unknown |
| *lpg1947* | *lpp1930* | *-* | *-* | - | - | - | - |  | Unknown |
| *lpg1948* | *-* | *-* | *-* | - | - | - | - | legLC4 | Leucine-rich repeats, coiled-coil |
| *lpg1949* | *lpp1931* | *lpl1918* | *lpc1422* | - | - | - | - |  | Unknown |
| *lpg1950* | *lpp1932* | *lpl1919* | *lpc1423* | *llo1397* | **+** | **+** | **+** | *ralF* | Sec-7 |
| *lpg1953* | *lpp1935* | *lpl1922* | *lpc1426* | *-* | *-* | *-* | *-* | *legC4* | Coiled-coil |
| *lpg1958* | *lpp1940* | *-* | *-* | *-* | *-* | *-* | *-* | *legL5* | Leucine-rich repeats |
| *lpg1960* | *lpp1942* | *-* | *lpc1437* | *llo0565* | **+** | **+** | **+** | *lirA* | Unknown |
| *lpg1962* | *lpp1946* | *lpl1936* | *lpc1440* | *-* | *-* | *-* | *-* | *lirB* | Peptidyl-prolyl cis-trans isomerase A (rotamase A) |
| *lpg1963* | - | - | *lpc1442* | - | - | - | - | *pieA/lirC* | Unknown |
| *lpg1964* | - | - | *-* | - | - | - | - | *pieB/lirD* | Unknown |
| *lpg1965* | - | - | *lpc1443* | - | - | - | - | *pieC/lirE* | Unknown |
| *lpg1966* | *lpp1947* | - | *lpc1446* | - | - | - | - | *pieD/lirF* | Unknown |
| *lpg1969* | *lpp1952* | *lpl1941* | *lpc1452* | *llo3131* | **+** | **+** | **+** | *pieE* | Unknown |
| *lpg1972* | *lpp1955* | *lpl1950* | *lpc1459* | - | - | - | - | *pieF* | Unknown |
| *lpg1975* | *lpp1959* | *lpl1953* | *lpc1462* | - | - | - | - |  | Unknown |
| *lpg1976* | *lpp1959* | *lpl1953* | *lpc1462* | - | - | - | - | *pieG/legG1* | Regulator of chromosome condensation RCC |
| *lpg1978* | *lpp1961* | *lpl1955* | *lpc1464* | *-* | *-* | *-* | *-* | *setA* | Putative Glycosyltransferase |
| *lpg2137* | *lpp2076* | *lpl2066* | *lpc1586* | *-* | *-* | *-* | *-* | *legK2* | STPK |
| *lpg2144* | *lpp2082* | *lpl2072* | *lpc1593* | *-* | *-* | *-* | *-* | *ankB/legAU13ceg27* | Ankyrin repeat, F-box |
| *lpg2155* | *lpp2094* | *lpl2083* | *lpc1604* | *llo3096* | **+** | **+** | **+** | *sidJ* | Unknown |
| *lpg2157* | *lpp2096* | *lpl2085* | *lpc1618* | *-* | *-* | *-* | *-* | *sdeC* | Unknown |
| *lpg2166* | *lpp2104* | *lpl2093* | *lpc1626* | *llo2398* | **+** | **+** | **+** |  | Unknown |
| *lpg2176* | *lpp2128* | *lpl2102* | *lpc1635* | *-* | *-* | *-* | *-* | *legS2* | Putative Sphingosine-1-phosphate lyase 1 (SP-lyase) |
| *lpg2200* | *lpp2150* | *lpl2124* | *lpc1664* | *llo0140* | **+** | **+** | **+** | *cegC4* | Unknown |
| *lpg2215* | *lpp2166* | *lpl2140* | *lpc1680* | - | - | - | - | *legA2* | Unknown |
| *lpg2216* | *lpp2167* | *lpl2141* | *lpc1681* | - | - | - | - |  | Unknown |
| *lpg2222* | *lpp2174* | *lpl2147* | *lpc1689* | - | - | - | - | *lpnE* | Putative Beta-lactamase |
| *lpg2224* | - | - | - | - | - | - | - | *ppgA* | Regulator of chromosome condensation |
| *lpg2248* | *lpp2202* | *lpl2174* | *lpc1717* | - | - | - | - |  | Unknown |
| *lpg2298* | *lpp2246* | *lpl2217* | *lpc1763* | *llo1707* | **+** | **+** | **+** | *ylfA/legC7* | Coiled-coil |
| *lpg2300* | *lpp2248* | *lpl2219* | *lpc1765* | *llo0584* | **+** | **+** | **+** | *ankH/legA3/ankW* | Ankyrin repeat, NFkappaB inhibitor |
| *lpg2322* | *lpp2270* | *lpl2242* | *lpc1789* | *llo0570* | **+** | **+** | **+** | *ankK/legA5* | Ankyrin repeat |
| *lpg2327* | *lpp2275* | *lpl2247* | *lpc1794* | - | - | - | - |  | Unknown |
| *lpg2328* | *lpp2276* | *lpl2248* | *lpc1795* | - | - | - | - |  | Unknown |
| *lpg2392* | *lpp2459* | *lpl2316* | *lpc2085* | - | - | - | - | *legL6* | Leucine-rich repeats |
| *lpg2400* | *-* | *lpl2323* | *-* | - | - | - | - | *legL6* | Leucine-rich repeats |
| *lpg2406* | *lpp2472* | *lpl2329* | *lpc2070* | *llo2172* | **+** | **+** | **+** |  | Unknown |
| *lpg2407* | *lpp2474* | *-* | *lpc2069* | *-* | *-* | *-* | *-* |  | Unknown |
| *lpg2409* | *lpp2476* | *lpl2332* | *lpc2067* | *-* | *-* | *-* | *-* | *ceg29* | Unknown |
| *lpg2410* | *lpp2479* | *lpl2334* | *lpc2065* | *-* | *-* | *-* | *-* | *vpdA* | Acyl transferase/acyl hydrolase/lysophospholipase |
| *lpg2411* | *lpp2480* | *lpl2335* | *lpc2064* | *llo2227* | **+** | **+** | **+** |  | Unknown |
| *lpg2422* | *lpp2487* | *lpl2345* | *lpc2055* | *llo1650* | **+** | **+** | **+** |  | Unknown |
| *lpg2433* | *lpp2500* | *lpl2353* | *lpc2043* | *-* | *-* | *-* | *-* |  | Unknown |
| *lpg2452* | *lpp2517* | *lpl2370* | *lpc2026* | *-* | *-* | *-* | *-* | *ankF/legA14/ceg31* | Ankyrin repeat |
| *lpg2456* | *lpp2522* | *lpl2375* | *lpc2020* | *llo0365* | **+** | **+** | **+** | *ankD/legA15* | Ankyrin repeat |
| *lpg2464* | *-* | *lpl2384* | *-* | *-* | *-* | *-* | *-* | *sidM/drrA* | Unknown |
| *lpg2465* | *-* | *lpl2385* | *-* | *-* | *-* | *-* | *-* | *sidD* | Unknown |
| *lpg2490* | *lpp2555* | *lpl2411* | *lpc1987* | *llo0796* | **+** | **+** | **+** | *lepB* | Coiled-coil, Rab1 GAP |
| *lpg2504* | *lpp2572* | *lpl2426* | *lpc1967* | *llo2525* | **+** | **+** | **+** |  | Unknown |
| *lpg2508* | *lpp2576* | *lpl2430* | *lpc1963* | *-* | *-* | *-* | *-* | *sdjA* | Unknown |
| *lpg2511* | *lpp2579* | *lpl2433* | *lpc1959* | *llo3098* | **+** | **+** | **+** | *sidC* | PI(4)P binding domain |
| *lpg2523* | *-* | *-* | *-* | *-* | *-* | *-* | *-* |  | Unknown |
| *lpg2527* | *lpp2592* | *lpl2447* | *lpc1944* | *llo3335* | **+** | **+** | **+** |  | Unknown |
| *lpg2529* | *lpp2594* | *lpl2449* | *lpc1942* | *llo2238* | **+** | **+** | **+** |  | Unknown |
| *lpg2556* | *lpp2626* | *lpl2481* | *lpc1906* | *llo2218* | **+** | **+** | **+** | *legK3* | STPK |
| *lpg2584* | *lpp2637* | *lpl2507* | *lpc0561* | *-* | *-* | *-* | *-* | *sidF* | Unknown |
| *lpg2591* | *lpp2644* | *lpl2514* | *lpc0551* | *llo0626* | **+** | **+** | **+** | *ceg33* | Unknown |
| *lpg2603* | *lpp2656* | *lpl2526* | *lpc0539* | *-* | *-* | *-* | *-* |  | Unknown |
| *lpg2718* | *lpp2775* | *lpl2646* | *lpc0415* | *-* | *-* | *-* | *-* | *wipA* | Unknown |
| *lpg2744* | *lpp2800* | *lpl2669* | *lpc0386* | *-* | *-* | *-* | *-* |  | Unknown |
| *lpg2793* | *lpp2839* | *lpl2708* | *lpc3079* | *-* | *-* | *-* | *-* | *lepA* | Coiled-coil |
| *lpg2804* | *lpp2850* | *lpl2719* | *lpc3090* | *llo0267* | **+** | **+** | **+** |  | Unknown |
| *lpg2826* | *-* | *lpl2741* | *lpc3113* | *-* | *-* | *-* | *-* |  | Unknown |
| *lpg2829* | *lpp2883* | *-* | *-* | *-* | *-* | *-* | *-* | *sidH* | Unknown |
| *lpg2830* | *lpp2887* | *-* | *-* | *-* | *-* | *-* | *-* | *lubX/legU2* | U box motif |
| *lpg2831* | *lpp2888* | *lpl4276* | *-* | *-* | *-* | *-* | *-* | *VipD* | Patatin-like phospholipase |
| *lpg2862* | *-* | *-* | *-* | *-* | *-* | *-* | *-* | *Lgt2/legC8* | Coiled-coil |
| *lpg2999* | *lpp3071* | *lpl2927* | *lpc3315* | *-* | *-* | *-* | *-* | *legP* | Astacin protease |

List of substrates based on Isberg *et al* 2008; de Felipe *et al* 2008; Ninio *et al* 2009
