# Supplementary material for: Analysis of the Legionella longbeachae Genome and Transcriptome Uncovers Unique Strategies to Cause Legionnaires' Disease
Source: PLoS Genet. 2010 Feb 19;6(2):e1000851. doi: 10.1371/journal.pgen.1000851 (PMC2824747; doi:10.1371/journal.pgen.1000851)
Supplement: Table S4 — Putative capsule and LPS encoding genes in L. longbeachae and its comparison to L. pneumophila Paris. (0.14 MB DOC) [file pgen.1000851.s010.doc]

**Table S4: Putative capsule and LPS encoding genes in *L. longbeachae* and its comparison to *L. pneumophila* Paris**

| **ORF** | **Gene NSW150 (Sg1)** | **Name** | **Annotation** | **% id with *L. longbeachae* ATCC39642 (Sg1)** | **% id with *L. longbeachae* 98072**  **(Sg2)** | **% id with *L. longbeachae* C-4E7**  **(Sg2)** | **% id with *L. pneumophila*** |
| --- | --- | --- | --- | --- | --- | --- | --- |
| 1 | *llo3148* | *cpsB* | Mannose-1-phosphate guanyltransferase | 98% | 98% | 98% | 45% *cpsB* |
| 2 | *llo3149* | *ctrA* | Capsule polysaccharide export protein ctrA precursor | 97% | 97% | 97% | - |
| 3 | *llo3150* | *ctrD* | Capsule polysaccharide export ATP-binding protein ctrD | 98% | 98% | 98% | 35% *wzt* |
| 4 | *llo3151* | *ctrC* | Capsule polysaccharide export inner-membrane protein ctrC | 91% | 91% | 91% | 21% *wzm* |
| 5 | *llo3152* | *ctrB* | Capsule polysaccharide export inner-membrane protein ctrB | 97% | 96% | 96% | - |
| 6 | *llo3153* | *_* | Mannosyltransferase | 99% | 98% | 98% | - |
| 7 | *llo3154* | *_* | Glycosyltransferase | 99% | 99% | 99% | 26% *capM* |
| 8 | *llo3155* | *ugd* | UDP-glucose 6-dehydrogenase | 100% | 99% | 99% | - |
| 9 | *llo3156* | *_* | Glycosyltransferase | 99% | 98% | 98% | 22% *lpl2477* |
| 10 | *llo3157* | *_* | Protein of unknown function | 99% | 99% | 99% | - |
| 11 | *llo3158* | *_* | Protein of unknown function | 96% | 95% | 95% | - |
| 12 | *llo3159* | *_* | Protein of unknown function | 100% | 99% | 99% | - |
| 13 | *llo3160* | *_* | Protein of unknown function | 100% | 99% | 99% | - |
| 14 | *llo3161* | *_* | Glycosyltransferase, family 2 | 99% | 98% | 98% | - |
| 15 | *llo3162* | *_* | N-acylneuraminate cytidylyltransferase | 100% | 97% | 97% | 26% *neuA* |
| 16 | *llo3163* | *_* | D-isomer specific 2-hydroxyacid dehydrogenase, NAD-binding | 100% | 99% | 99% | 33% *lpp2733* |
| 17 | *llo3164* | *_* | Putative short-chain dehydrogenase/reductase | 95% truncated | 95% truncated | 95% truncated | - |
| 18 | *llo3165* | *_* | Glycosyltransferase | - | - | - | - |
| 19 | *llo3166* | *galE2* | UDP-galactose-4-epimerase | - | - | - | - |
| 20 | *llo3167* | *gmd* | GDP-D-mannose dehydratase, NAD(P)-binding | 85% | 85% | 85% | - |
| 21 | *llo3168* | *_* | Protein of unknown function | - | - | - | - |
| 22 | *llo3169* | *_* | Protein of unknown function | 79% pseudo | 79% pseudo | 79% pseudo | - |
| 23 | *llo3170* | *_* | Protein of unknown function | 99% | 99% | 99% | - |
| 24 | *llo3171* | *galU* | Glucose-1-phosphate uridylyltransferase | 99% | 99% | 99% | 24% *lpp0826* |
| 25 | *llo3172* | *galE* | UDP-galactose-4-epimerase | 99% | 99% | 99% | - |
| 26 | *llo3173* | *fcl* | Bifunctional GDP-fucose synthetase | 99% | 99% | 98% frameshift | - |
| 27 | *llo3174* | *_* | Glycosyltransferase (fragment) | 100% | 99% | 99% | - |
| 28 | *llo3175* | *_* | Oxidoreductase, short-chain dehydrogenase/reductase | 100% | 99% | 99% | 31% *lpp1382* |
| 29 | *llo3176* | *_* | Glycosyltransferase | 100% | 98% | 98% | 25% *lpp3020* |
| 30 | *llo3177* | *_* | UDP-Glycosyltransferase | 100% | 98% | 98% | - |
| 31 | *llo3178* | *_* | Putative aminotranferase | 100% | 98% | 98% | - |
| 32 | *llo3179* | *wcbR* | Putative type I polyketide synthase WcbR | 100% | 99% | 99% | - |
| 33 | *llo3180* | *capI* | NAD-dependent epimerase/dehydratase protein | 100% | 99% | 99% | 29% *rmlB* |
|  |  |  |  |  |  |  |  |
| 34 | *llo0217* | wecB | UDP-N-acetylglucosamine 2-epimerase | 99% | 99% | 99% | 27% *neuC* |
| 35 | *llo0218* | _ | Membrane protein of unknown function | 100% | - | - | - |
| 36 | *llo0219* | *rfbG* | CDP-glucose 4,6-dehydratase | 100% | - | - | - |
| 37 | *llo0220* | *rfbF* | Glucose-1-phosphate cytidylyltransferase | 100% | - | - | - |
| 38 | *llo0221* | _ | Membrane protein, GtrA-like family protein | 100% | - | - | - |
| 39 | *llo0222* | _ | Methyltransferase | 100% | - | - | - |
| 40 | *llo0223* | _ | Glycosyl transferase, family 2 precursor | 100% | - | - | 36% *yfdH* |
| 41 | *llo0224* | _ | Methyltransferase | 100% | - | - | - |
| 42 | *llo0225* | _ | Oxidoreductase | 100% | - | - | - |
| 43 | *llo0226* | _ | Putative acyltransferase | 100% | - | - | - |
| 44 | *llo0227* | _ | LPS biosynthesis protein, similar to wzxE translocase | 100% | 98% | 98% | - |
| 45 | *llo0228* | _ | Protein of unknown function | 100% | 99% | 99% | - |
| 46 | *llo0229* | *wcfH* | Putative deacetylase | 100% | 99% | 99% | - |
| 47 | *llo0230* | _ | Glycosyl transferase, family 2 | 100% | 99% | 99% | - |
| 48 | *llo0231* | _ | Membrane protein of unknown function | 100% | 97% | 97% | - |
| 49 | *llo0232* | _ | Glycosyl transferase, group 1 family protein | 99% | 98% | 98% | - |
| 50 | *llo0233* | *wbwI* | O-acetyltransferase | 100% | 99% | 99% | 25% *lpl2879* |
| 51 | *llo0234* | _ | Glycosyl transferase, group 1 family protein | 100% | 99% | 99% | - |
| 52 | *llo0235* | _ | Putative dTDP-4-dehydrorhamnose reductase | 100% | 99% | 99% | 28% *rmlD* |
| 53 | *llo0236* | _ | Putative NAD dependent epimerase/dehydratase | 100% | 99% | 99% | - |
