# Supplementary material for: Analysis of the Legionella longbeachae Genome and Transcriptome Uncovers Unique Strategies to Cause Legionnaires' Disease
Source: PLoS Genet. 2010 Feb 19;6(2):e1000851. doi: 10.1371/journal.pgen.1000851 (PMC2824747; doi:10.1371/journal.pgen.1000851)
Supplement: Table S5 — Analysis of the FlgD, FleR/S, and FliA/FleN encoding regions in L. longbeachae. (0.05 MB DOC) [file pgen.1000851.s011.doc]

**Table S5: Analysis of the FlgD, FleR/S and FliA/FleN encoding regions in *L. longbeachae***

| **Strain** | **Serogroup** | **Origin** | **Genomic region** | |  |
| --- | --- | --- | --- | --- | --- |
|  |  |  | *flgD* | *fleR-fleS* | *fliA-fleN* |
| NSW | 1 | Australia | - | - | - |
| ATCC33484° | 2 | ATCC | - | - | - |
| ATCC33462° | 1 | ATCC | - | - | - |
| L6C9^+^ | 1 | Australia | - | - | - |
| LA24^+^ | 1 | Australia | - | - | - |
| D493^+^ | 1 | Australia | - | - | - |
| D1753^+^ | 1 | Australia | - | - | - |
| A5H5^+^ | 1 | Australia | - | - | - |
| D880^+^ | 1 | Australia | - | - | - |
| K889^+^ | 1 | Australia | - | - | - |
| Atl5^+^ | 1 | Australia | - | - | - |
| A4C5^+^ | 1 | Australia | - | - | - |
| 98-072^+^ | 2 | USA | - | - | - |
| ATCC3346* | 1 | USA | - | - | - |
| D4968* | 2 | USA | - | - | - |
| C4E7^+^ | 2 | Australia | - | - | - |

° Obtained from the Institut Pasteur Culture Collection

^+^Kindly provided by Dr. H. Newton and Dr. L. Hartland

*Kindly provided by Dr. Y. Abu Kwaik
