# Supplementary material for: Analysis of the Legionella longbeachae Genome and Transcriptome Uncovers Unique Strategies to Cause Legionnaires' Disease
Source: PLoS Genet. 2010 Feb 19;6(2):e1000851. doi: 10.1371/journal.pgen.1000851 (PMC2824747; doi:10.1371/journal.pgen.1000851)
Supplement: Table S6 — Transcriptional regulators identified in L. longbeachae and their orthologs in L. pneumophila. (0.30 MB DOC) [file pgen.1000851.s012.doc]

**Table S6: Transcriptional regulators identified in *L. longbeachae* and their orthologs in *L. pneumophila***

|  | ***L. longbeachae*** | ***L. pneumophila*** | | | |
| --- | --- | --- | --- | --- | --- |
|  | **NSW150** | **Paris** | **Lens** | **Philadelphia** | **Corby** |
| **Lambda repressor-like,** | *llo0147* | *lpp0490* | *lpl0466* | *lpg0423* | *lpc2920* |
| **(Phage repressors PrpA, SinR, Xre)** | *llo0334* | *lpp2773* | *lpl2645* | *lpg2717* | *lpc0416* |
|  | *llo0654* |  |  |  |  |
|  | *llo1621* |  |  |  |  |
|  | *llo1853* | *lpp0165* | *lpl0147* | *lpg2096* | *lpc0166* |
|  | *llo2871* |  |  | *lpg1260* |  |
|  | *llo2913* |  |  |  |  |
|  | *llo2956* |  |  |  | *lpc1408* |
|  | *pllo0039* |  |  |  |  |
|  |  |  | *lpl0176* |  |  |
|  |  |  | *plpl0056* |  |  |
|  |  | *lpp0074* |  |  | *lpc0087* |
|  |  | *lpp1077* | *lpl1039* | *lpg4079* | *lpc2273* |
|  |  | *lpp1085* | *lpl1063* | *lpg1066* | *lpc2208* |
|  |  | *lpp1916* | *lpl1905* | *lpg1935* | *lpc1408* |
|  |  | *lpp2116* |  |  | *lpc0214* |
|  |  | *lpp2326* | *lpl1052* | *lpg4092* |  |
|  |  | *lpp2375* |  |  | *lpc1857* |
|  |  | *lpp2426* | *lpl2290* | *lpg2377* | *lpc4274* |
|  |  | *lpp0065* |  |  |  |
|  |  | *lpp0191* | *lpl0176* |  | *lpc0196* |
|  |  |  |  |  | *lpc2816* |
|  |  |  |  | *lpg2562* |  |
|  |  |  |  | *lpg2567* |  |
|  |  |  |  | *lpg2059* |  |
|  |  |  |  | *lpg2368* |  |
|  |  |  | *lpl2833* | *lpg2914* |  |
| **Two component regulators** | *llo0302* | *lpp2808* | *lpl2677* | *lpg2760* | *lpc3044* |
|  | *llo0753* |  |  |  |  |
| ***pmrA*** | *llo1159* | *lpp1255* | *lpl1254* | *lpg1292* | *lpc0716* |
|  | *llo1692* | *lpp2133* | *lpl2108* | *lpg2181* | *lpc1641* |
| ***cpxR*** | *llo1781* | *lpp1393* | *lpl1603* | *lpg1438* | *lpc0854* |
|  | *llo2216* |  |  |  |  |
| ***fleR*** | *llo2319* | *lpp1726* | *lpl1726* | *lpg1762* | *lpc1203* |
|  | *llo2400* |  |  |  |  |
|  | *llo2490* |  |  |  |  |
| ***letA*** | ***llo2653*** | ***lpp2699*** | ***lpl2571*** | ***lpg2646*** | ***lpc0493*** |
|  | *llo2778* |  |  |  |  |
|  | *llo3084* |  |  |  |  |
|  | *llo3187* |  |  |  |  |
| ***cheY*** | *llo3303* |  |  |  |  |
|  | *llo3317* | *lpp2523* | *lpl2376* | *lpg2457* | *lpc2019* |
|  | *llo3318* |  |  |  |  |
|  | *llo3320* | *lpp0112* | *lpl0098* | *lpg0098* | *lpc0117* |
|  |  | *lpp1176* | *lpl1182* | *lpg1174* | *lpc0640* |
|  |  | *lpp0781* | *lpl0752* | *lpg0715* | *lpc2578* |
|  |  | *lpp2083* | *lpl2073* | *lpg2145* | *lpc1594* |
|  |  | *lpp2788* | *lpl2657* | *lpg2732* | *lpc0401* |
| **LysR** | *llo0134* |  |  |  |  |
|  | *llo1089* |  |  |  |  |
|  | *llo1135* | *lpp2441* |  | *lpg2376* | *lpc2116* |
|  | *llo1616* |  | *lpl0326* |  |  |
|  | *llo1956* | *lpp1760* | *lpl1760* | *lpg1796* | *lpc1237* |
|  | *llo1981* | *lpp2445* | *lpl2301* | *lpg2383* | *lpc2107* |
|  | *llo2126* | *lpp0282* | *lpl0277* | *lpg0223* | *lpc0299* |
|  | *llo2211* | *lpp0235* | *lpl0235* | *lpg0173* | *lpc0255* |
|  | *llo2256* |  |  |  |  |
|  | *llo2772* | *lpp1778* | *lpl1779* | *lpg1815* | *lpc1259* |
|  | *llo3006* |  |  |  | *lpc2122* |
|  | *llo3017* |  |  |  |  |
|  | *llo3106* | *lpp0355* | *lpl0332* | *lpg0280* | *lpc0357* |
|  | *llo3199* |  |  | *lpg2288* |  |
|  | *llo3234* | *lpp0274* | *lpl0269* | *lpg0215* | *lpc0290* |
|  | *llo3401* | *lpp2077* | *lpl2067* | *lpg2138* | *lpc1587* |
|  |  | *lpp0057* | *lpl0055* | *lpg0055* | *lpc0061* |
|  |  | *lpp0348* | *lpl0326* | *lpg0274* | *lpc0350* |
|  |  | *lpp2060* | *lpl2050* |  | *lpc1568* |
|  |  | *lpp2467* | *lpl2325* | *lpg2402* | *lpc2076* |
|  |  | *lpp2862* |  |  |  |
| **Cold shock protein** | *llo0099* |  |  |  |  |
|  | *llo0240* | *lpp2878* | *lpl2740* | *lpg2825* | *lpc3112* |
|  | *llo0956* | *lpp2321* | *lpl1057* | *lpg1060* | *lpc2214* |
|  | *llo1310* | *lpp1207* | *lpl1213* | *lpg1205* | *lpc0673* |
|  | *llo2157* | *lpp2041* | *lpl2039* | *lpg2121* | *lpc1546* |
|  | *llo2797* | *lpp0493* | *lpl0469* | *lpg0426* | *lpc2917* |
|  |  | *lpp2908* | *lpl2762* | *lpg2850* | *lpc4325* |
| **Carbon storage regulator (Csr)** | *llo1813* | *lpp1551* | *lpl1432* | *lpg1593* | *lpc1020* |
|  | *llo1850* | *lpp0168* | *lpl0150* | *lpg1257* | *lpc1860* |
| ***csrA*** | ***llo2071*** | ***lpp0845*** | ***lpl0820*** | ***lpg0781*** | ***lpc2513*** |
|  | *llo2874* |  |  |  | *lpc0169* |
|  |  | *lpp1074* | *lpl1036* | *lpg1003* | *lpc2276* |
|  |  | *lpp2378* |  |  | *lpc1860* |
|  |  |  |  | *lpg2094* |  |
|  |  |  |  |  | *lpc2813* |
| **MerR** | *llo0515* |  |  |  |  |
|  | *llo1202* | *lpp1581* | *lpl1413* | *lpg1611* | *lpc1038* |
|  | *llo1820* |  |  | *lpg2058* |  |
|  | *llo2182* | *lpp0060* | *lpl0059* | *lpg0057* | *lpc0066* |
|  |  | *lpp1582* | *lpl1411* | *lpg1612* | *lpc1039* |
|  |  |  |  | *lpg1236* |  |
|  |  |  |  |  | *lpc2192* |
| **LuxR** | *llo1620* |  | *lpl2445* | *lpg2524* |  |
|  | *llo1636* |  |  |  |  |
|  | *llo1822* |  |  |  |  |
|  | *llo2217* | *lpp2627* | *lpl2482* | *lpg2557* | *lpc1905* |
|  | *llo2224* |  |  |  |  |
|  | *llo2671* |  |  |  |  |
|  | *llo3059* |  |  |  |  |
|  |  |  | *lpl2098* |  |  |
|  |  |  | *lpl2445* |  |  |
|  |  |  | *lpl1926* |  | *lpc1430* |
|  |  | *lpp1403* |  | *lpg1448* |  |
|  |  | *lpp1927* | *lpl1917a* | *lpg1946* | *lpc1419* |
|  |  | *lpp2317* |  |  | *lpc1838* |
| **MarR** | *llo0335* |  |  |  |  |
|  | *llo2139* |  |  |  |  |
|  |  | *lpp1210* | *lpl1216* | *lpg1208* | *lpc0677* |
|  |  | *lpp1214* | *lpl1220* | *lpg1212* | *lpc0681* |
|  | *pllo0063* |  |  |  |  |
| **Helix-turn-helix, Fis-type** | *llo2305* | *lpp1707* | *lpl1707* | *lpg1743* | *lpc1183* |
|  | *llo0406* | *lpp0606* | *lpl0587* | *lpg0542* | *lpc2758* |
|  | *llo1460* | *lpp1324* | *lpl1321* | *lpg1370* | *lpc0786* |
| **Helix-turn-helix motif, Crp-type** | *llo1422* |  |  |  |  |
| **Helix-turn-helix type 3** | *llo0970* |  |  |  |  |
| **Helix-turn-helix, AraC type** | *llo0109* |  |  |  |  |
|  | *llo0718* | *lpp2618* | *lpl2470* | *lpg2549* | *lpc1917* |
|  | *llo2588* | *lpp1152* | *lpl1156* | *lpg1150* |  |
|  |  |  |  | *lpg2163* |  |
|  |  | *lpp2002* | *lpl1997* | *lpg2020* | *lpc1504* |
| **Helix-turn-helix, HxlR type** | *llo1368* | *lpp0500* | *lpl0476* | *lpg0433* | *lpc2910* |
|  | *llo1673* | *lpp2079* | *lpl2069* | *lpg2140* | *lpc1589* |
| **Helix-turn-helix, type 11** | *llo1211* | *lpp2105* | *lpl2094* | *lpg2167* | *lpc1627* |
|  | *llo2070* | *lpp0847* | *lpl0822* | *lpg0783* | *lpc2509* |
| **GGDEF/EAL domains** | *llo1734* | *lpp1425* | *lpl1559* | *lpg1469* | *lpc0884* |
|  | *llo0090* |  |  |  |  |
|  | *llo0599* |  | *lpl2826* |  |  |
|  | *llo0887* | *lpp0942* | *lpl0912* | *lpg0879* | *lpc2413* |
|  | *llo1253* | *lpp0352* | *lpl0329* | *lpg0277* | *lpc0354* |
|  | *llo1377* |  |  |  |  |
|  | *llo1947* | *lpp1475* | *lpl1508* | *lpg1518* | *lpc0937* |
|  | *llo2005* |  |  |  |  |
|  | *llo3035* | *lpp0952* | *lpl0922* | *lpg0891* | *lpc2402* |
|  | *llo3083* |  |  |  |  |
|  | *llo3125* |  |  |  |  |
|  | *llo3244* |  |  |  |  |
|  | *llo3392* | *lpp0087* | *lpl0075* | *lpg0073* | *lpc0095* |
|  |  |  | *lpl2826* |  |  |
|  | *llo3414* | *lpp0299* | *lpl0283* | *lpg0230* | *lpc0305* |
|  |  | *lpp2477* | *lpl2333* |  | *lpc2066* |
|  |  | *lpp0029* | *lpl0030* | *lpg0029* | *lpc0030* |
|  |  | *lpp0219* | *lpl0219* | *lpg0155* | *lpc0236* |
|  |  | *lpp0220* | *lpl0220* | *lpg0156* |  |
|  |  | *lpp0440* | *lpl0416* | *lpg0373* | *lpc2970* |
|  |  | *lpp0809* | *lpl0780* | *lpg0744* | *lpc2549* |
|  |  | *lpp0891* | *lpl0860* | *lpg0829* | *lpc2467* |
|  |  | *lpp1114* | *lpl1118* | *lpg1114* | *lpc2139* |
|  |  | *lpp1170* | *lpl1176* | *lpg1168* | *lpc0633* |
|  |  | *lpp1311* | *lpl1308* | *lpg1357* | *lpc0771* |
|  |  | *lpp2071* | *lpl2061* | *lpg2132* | *lpc1579* |
|  |  | *lpp2324* | *lpl1054* | *lpg1057* |  |
|  |  | *lpp2355* |  | *lpg1025* | *lpc2253* |
|  |  | *lpp2695* | *lpl2567b* | *lpg2642* | *lpc0497* |
|  |  | *lpp2708* | *lpl2581* | *lpg2655* | *lpc0484* |
|  |  |  | *lpl0694* |  | *lpc2635* |
|  |  | *lpp0127* |  | *lpg0113* |  |
|  |  |  |  |  | *lpc0072* |
|  |  |  |  |  | *lpc0864* |
|  |  | *lpp0592* | *lpl0573* | *lpg0527* | *lpc2775* |
| **Transcription factor CarD** | *llo1943* | *lpp1016* | *lpl0983* | *lpg0954* | *lpc2333* |
| **AraC, arabinose-binding/dimerisation** | *llo1907* |  |  |  |  |
| **ArsR** | *llo1416* |  | *lpl1048* |  |  |
|  | *llo2143* | *lpp2780* |  | *lpg2723* | *lpc0410* |
| **DeoR** | *llo3323* | *lpp0108* | *lpl0093* | *lpg0094* | *lpc0113* |
| **TetR** | *llo2696* | *lpp1949* | *lpl1938* | *lpg1967* | *lpc1449* |
|  | *pllo0054* |  |  |  |  |
| **AsnC/Lrp** | *llo0685* |  |  |  |  |
|  | *llo2410* | *lpp2585* | *lpl2439* | *lpg2517* | *lpc1953* |
|  | *llo2523* | *lpp1442* | *lpl1542* | *lpg1486* | *lpc0901* |
| **Antitermination NusG** | *llo0474* | *lpp0382* | *lpl0357* | *lpg0317* | *lpc3026* |
| **Proline dehydrogenase (PutA)** | *llo1700* | *lpp1661* | *lpl1655* | *lpg1696* | *lpc1125* |
| **Bordetella pertussis Bvg accessory factor** | *llo0853* | *lpp0973* | *lpl0943* | *lpg0911* | *lpc2380* |
| **CopG-like DNA-binding** | *llo2646* |  |  |  |  |
| **Ferric-uptake regulator** | *llo0531* | *lpp0438* | *lpl0414* | *lpg4034* | *lpc2972* |
|  | *llo0711* | *lpp0302* | *lpl0285* | *lpg0232* | *lpc0307* |
| **Homeobox** | *llo0174* | *lpp2923* | *lpl2777* | *lpg2865* | *lpc3150* |
|  | *llo1969* | *lpp0919* | *lpl0888* | *lpg0857* | *lpc2438* |
| **NusB/RsmB/TIM44** | *llo0628* | *lpp2646* | *lpl2516* | *lpg2593* | *lpc0549* |
|  | *llo0826* | *lpp0793* | *lpl0764* | *lpg0727* | *lpc2565* |
| **Penicillinase repressor** | *llo1199* | *lpp1590* | *lpl1403* | *lpg1620* | *lpc1047* |
| **Peptidase S24, LexA/MucA/RumA/RuvA** | *llo1863* |  |  | *lpg1704* | *lpc1133* |
|  | *pllo0066* |  | *plpl0056* |  |  |
|  | *llo1987* | *lpp1039* | *lpl1004* | *lpg1231* |  |
|  | *llo2659* |  |  |  |  |
| **Transcription elongation factor,** | *llo0088* |  |  |  |  |
| **GreA/GreB region, prokaryotic** | *llo3087* | *lpp2677* | *lpl2549* | *lpg2624* | *lpc0517* |
| **Trp repressor-like fold** | *llo1305* | *lpp1203* | *lpl1209* | *lpg1201* | *lpc0668* |
