# Supplementary material for: Analysis of the Legionella longbeachae Genome and Transcriptome Uncovers Unique Strategies to Cause Legionnaires' Disease
Source: PLoS Genet. 2010 Feb 19;6(2):e1000851. doi: 10.1371/journal.pgen.1000851 (PMC2824747; doi:10.1371/journal.pgen.1000851)
Supplement: Table S7 — Genes upregulated in L. longbeachae in exponential growth phase. (0.23 MB DOC) [file pgen.1000851.s013.doc]

**Table S7: Genes upregulated in *L. longbeachae* in exponential growth phase**

| **Gene** | **NSW150 specific** | **Predicted fucntion** | **Gene name** |
| --- | --- | --- | --- |
| *llo0015* |  | Hypothetical protein | *_* |
| *llo0044* | specific | Thaumatin domain-containing protein | *_* |
| *llo0062* |  | Alkyl hydroperoxide reductase | *ahpC* |
| *llo0065* |  | Major outer membrane protein homolog | *_* |
| *llo0083* |  | Transcription termination factor | *rho* |
| *llo0099* |  | Cold shock DNA binding domain protein | *cspE* |
| *llo0159* |  | Similar to glucose inhibited division protein B GidB | *gidB* |
| *llo0223* |  | Putative glycosyl transferase, group 2 family protein | *_* |
| *llo0224* | specific | Putative methyltransferase | *_* |
| *llo0236* | specific | Putative NAD dependent epimerase/dehydratase | *_* |
| *llo0255* |  | Similar to aminopeptidase | *_* |
| *llo0270* |  | Similar to predicted RNA-binding protein | *yhbY* |
| *llo0275* |  | Triosephosphate isomerase | *tpi* |
| *llo0276* |  | Preprotein translocase membrane subunit | *secG* |
| *llo0277* |  | NADH dehydrogenase I chain A | *nuoA* |
| *llo0278* |  | NADH-quinone oxidoreductase subunit B | *nuoB* |
| *llo0280* |  | NADH dehydrogenase subunit D | *nuoD* |
| *llo0281* |  | NADH:ubiquinone oxidoreductase, chain E | *nuoE* |
| *llo0282* |  | NADH:ubiquinone oxidoreductase, chain F | *nuoF* |
| *llo0283* |  | NADH dehydrogenase I chain G | *nuoG* |
| *llo0284* |  | NADH:ubiquinone oxidoreductase, membrane subunit H | *nuoH* |
| *llo0289* |  | NADH:ubiquinone oxidoreductase, membrane subunit M | *nuoM* |
| *llo0296* |  | 30S ribosomal subunit protein S15 | *rpsO* |
| *llo0299* |  | Similar to HIT( Histidine triad nucleotide-binding protein) family protein | *hinT* |
| *llo0300* |  | Inorganic pyrophosphatase | *ppa* |
| *llo0338* |  | Translation initiation factor IF-3 | *infC* |
| *llo0339* |  | 50S ribosomal protein L35 | *rpmI* |
| *llo0340* |  | 50S ribosomal subunit protein L20 | *rplT* |
| *llo0370* |  | Similar to methyltransferase proteins | *yhhF* |
| *llo0406* |  | Similar to DNA-binding proteins Fis | *fis* |
| *llo0472* |  | Protein chain elongation factor EF-Tu (duplicate of tufA) | *tufA-2* |
| *llo0473* |  | Preprotein translocase secE subunit | *secE* |
| *llo0474* |  | Transcription termination factor | *nusG* |
| *llo0475* |  | 50S ribosomal subunit protein L11 | *rplK* |
| *llo0476* |  | 50S ribosomal subunit protein L1 | *rplA* |
| *llo0477* |  | 50S ribosomal subunit protein L10 | *rplJ* |
| *llo0478* |  | 50S ribosomal subunit protein L7/L12 | *rplL* |
| *llo0479* |  | RNA polymerase, beta subunit | *rpoB* |
| *llo0480* |  | RNA polymerase, beta prime subunit | *rpoC* |
| *llo0481* |  | 30S ribosomal subunit protein S12 | *rpsL* |
| *llo0482* |  | 30S ribosomal subunit protein S7 | *rpsL* |
| *llo0483* |  | Protein chain elongation factor EF-G, GTP-binding | *fusA* |
| *llo0484* |  | Protein chain elongation factor EF-Tu (duplicate of tufA) | *tufA* |
| *llo0485* |  | 30S ribosomal subunit protein S10 | *rpsJ* |
| *llo0486* |  | 50S ribosomal subunit protein L3 | *rplC* |
| *llo0487* |  | 50S ribosomal subunit protein L4 | *rplD* |
| *llo0488* |  | 50S ribosomal subunit protein L23 | *rplW* |
| *llo0489* |  | 50S ribosomal subunit protein L2 | *rplB* |
| *llo0490* |  | 30S ribosomal subunit protein S19 | *rpsS* |
| *llo0491* |  | 50S ribosomal subunit protein L22 | *rplV* |
| *llo0492* |  | 30S ribosomal subunit protein S3 | *rpsC* |
| *llo0493* |  | 50S ribosomal subunit protein L16 | *rplP* |
| *llo0494* |  | 50S ribosomal subunit protein L29 | *rpmC* |
| *llo0495* |  | 30S ribosomal subunit protein S17 | *rpsQ* |
| *llo0496* |  | 50S ribosomal subunit protein L14 | *rplN* |
| *llo0497* |  | 50S ribosomal subunit protein L24 | *rplX* |
| *llo0498* |  | 50S ribosomal subunit protein L5 | *rplE* |
| *llo0499* |  | 30S ribosomal subunit protein S14 | *rpsN* |
| *llo0500* |  | 30S ribosomal subunit protein S8 | *rpsH* |
| *llo0501* |  | 50S ribosomal subunit protein L6 | *rplF* |
| *llo0502* |  | 50S ribosomal subunit protein L18 | *rplR* |
| *llo0503* |  | 30S ribosomal subunit protein S5 | *rpsE* |
| *llo0504* |  | 50S ribosomal subunit protein L30 | *rpmD* |
| *llo0505* |  | 50S ribosomal subunit protein L15 | *rplO* |
| *llo0506* |  | preprotein translocase membrane subunit | *secY* |
| *llo0507* |  | 30S ribosomal subunit protein S13 | *rpsM* |
| *llo0508* |  | 30S ribosomal subunit protein S11 | *rpsK* |
| *llo0509* |  | 30S ribosomal subunit protein S4 | *rpsD* |
| *llo0510* |  | RNA polymerase, alpha subunit | *rpoA* |
| *llo0511* |  | 50S ribosomal subunit protein L17 | *rplQ* |
| *llo0514* |  | Single-stranded DNA-binding protein | *ssb* |
| *llo0522* |  | Similar to 3-oxoacyl-[acyl-carrier-protein]synthase II | *fabF* |
| *llo0523* |  | Similar to 3-oxoacyl-[acyl-carrier-protein] synthase beta chain | *_* |
| *llo0524* |  | Similar to lipid A biosynthesis acyltransferase | *waaM* |
| *llo0535* |  | Similar to membrane-fusion protein involved in transport | *_* |
| *llo0567* |  | Similar to exodeoxyribonuclease VII, small subunit | *xseB* |
| *llo0578* |  | Similar to conserved hypothetical protein | *yibN* |
| *llo0597* | specific | Hypothetical protein | *_* |
| *llo0611* |  | Conserved protein of unknown function |  |
| *llo0630* |  | Similar to polypeptide deformylase | *def* |
| *llo0636* |  | Similar to pyruvate/2-oxoglutarate dehydrogenase complex | *_* |
| *llo0711* |  | Similar to Fe2+/Zn2+ uptake regulation proteins | *_* |
| *llo0765* |  | Hypothetical protein | *_* |
| *llo0818* |  | Valyl-tRNA synthetase | *valS* |
| *llo0819* |  | Highly similar to multidrug efflux transporter | *_* |
| *llo0821* |  | Hypothetical protein | *_* |
| *llo0859* |  | Hemin binding protein (Hbp) homolog | *_* |
| *llo0932* |  | Similar to protein-export membrane protein SecD | *secD* |
| *llo1010* | specific | Hypothetical protein | *_* |
| *llo1056* |  | Similar to outer membrane protein | *_* |
| *llo1095* |  | Hypothetical protein | *_* |
| *llo1148* |  | Hypothetical protein | *_* |
| *llo1174* |  | Tryptophan synthase, alpha subunit | *trpA* |
| *llo1276* |  | Similar to GTP-binding elongation factor | *lepA* |
| *llo1277* |  | leader peptidase (signal peptidase I) | *lepB* |
| *llo1282* |  | Peptidyl-prolyl cis/trans isomerase (trigger factor) | *tig* |
| *llo1283* |  | Proteolytic subunit of ClpA-ClpP and ClpX-ClpP ATP-dependent serine proteases | *clpP* |
| *llo1286* |  | Putative DNA-binding protein HU-beta | *hubB* |
| *llo1437* |  | Hypothetical protein, weakly similar to eukaryotic protein | *_* |
| *llo1438* | specific | Hypothetical protein | *_* |
| *llo1439* |  | Similar to ribonucleoside-diphosphate reductase, beta subunit | *rir2* |
| *llo1440* |  | Similar to ribonucleoside-diphosphate reductase, alpha subunit | *rir1* |
| *llo1460* |  | Similar to DNA-binding protein Fis | *fis* |
| *llo1462* |  | Similar to oxidoreductase | *_* |
| *llo1463* |  | Similar to ribonuclease HII | *rnhB* |
| *llo1479* |  | 50S ribosomal subunit protein L32 | *rpmF* |
| *llo1480* |  | Fatty acid/phospholipid synthesis protein | *plsX* |
| *llo1517* |  | Pyruvate dehydrogenase E2 (dihydrolipoamide acetyltransferase) | *odp* |
| *llo1518* |  | Similar to pyruvate dehydrogenase E1 (beta subunit) | *_* |
| *llo1519* |  | Similar to pyruvate dehydrogenase, (E1 alpha subunit) | *odpA* |
| *llo1555* |  | Similar to probable methylisocitrate lyase | *prpB* |
| *llo1660* | specific | Hypothetical protein | *_* |
| *llo1698* |  | Aconitate hydratase 1 | *acnA* |
| *llo1738* |  | Nucleoside triphosphate pyrophosphohydrolase, marked preference for dGTP | *mutT* |
| *llo1739* |  | Preprotein translocase subunit, ATPase | *secA* |
| *llo1759* |  | Putative lipoamide dehydrogenase | *lpd* |
| *llo1761* |  | Pyruvate dehydrogenase, decarboxylase component E1, thiamin-binding | *aceE* |
| *llo1766* |  | Putative penicillin-binding protein precursor (D-alanyl-D-alaninecarboxypeptidase) | *dacA* |
| *llo1802* |  | DNA gyrase (type II topoisomerase), subunit A | *gyrA* |
| *llo1809* |  | 50S ribosomal subunit protein L9 | *rplI* |
| *llo1811* |  | 30S ribosomal subunit protein S18 | *rpsR* |
| *llo1812* |  | 30S ribosomal subunit protein S6 | *rpsF* |
| *llo1963* |  | Heme lyase, CcmH subunit (fragment) | *ccmH* |
| *llo2018* |  | Weakly similar to anti-anti-sigma factor | *_* |
| *llo2043* |  | Isocitrate dehydrogenase, specific for NADP+; e14 prophage | *icd* |
| *llo2054* |  | Similar to phosphoenolpyruvate synthase | *ppsA* |
| *llo2087* |  | Peptidoglycan-associated outer membrane lipoprotein | *pal* |
| *llo2245* |  | Similar to methionine aminopeptidase, type I | *map* |
| *llo2253* |  | Membrane ATPase of the MinC-MinD-MinE system | *minD* |
| *llo2305* |  | Similar to DNA-binding protein fis | *_* |
| *llo2328* |  | Translation initiation factor IF-1 | *infA* |
| *llo2330* |  | Similar to 3-ketoacyl-CoA thiolase (thiolase I, acetyl-CoA transferase) | *fadA* |
| *llo2332* |  | Similar to aldehyde dehydrogenase | *_* |
| *llo2333* |  | Similar to dehydrogenase | *_* |
| *llo2335* |  | Leucyl-tRNA synthetase | *leuS* |
| *llo2336* |  | Putative rare lipoprotein B | *rlpB* |
| *llo2340* |  | Conserved hypothetical protein | *_* |
| *llo2434* |  | Hypothetical protein | *_* |
| *llo2475* |  | Hypothetical protein | *_* |
| *llo2500* |  | Similar to serine protease | *_* |
| *llo2514* |  | Hypothetical protein | *_* |
| *llo2574* |  | Hypothetical protein | *_* |
| *llo2575* |  | Similar to peptidoglycan GlcNAc deacetylase proteins | *_* |
| *llo2592* | specific | Hypothetical protein | *_* |
| *llo2620* |  | Similar to partition protein ParB | *parB* |
| *llo2634* |  | Hypothetical protein | *_* |
| *llo2642* |  | 30S ribosomal subunit protein S20 | *rpsT* |
| *llo2661* |  | 50S ribosomal subunit protein L27 | *rpmA* |
| *llo2662* |  | 50S ribosomal subunit protein L21 | *rplU* |
| *llo2663* |  | Similar to 50S ribosomal subunit protein L25, RplY | *rplY* |
| *llo2684* |  | UDP-N-acetylglucosamine acetyltransferase | *lpxA* |
| *llo2685* |  | (3R)-hydroxymyristol acyl carrier protein dehydratase | *fabZ* |
| *llo2686* |  | UDP-3-O-(3-hydroxymyristoyl)-glucosamine N-acyltransferase | *lpxD* |
| *llo2687* |  | Similar to putative outer membrane proteins | *_* |
| *llo2705* |  | Putative ankyrin repeat protein | *_* |
| *llo2707* |  | 50S ribosomal subunit protein L28 | *rpmB* |
| *llo2721* |  | Zinc metalloproteinase precursor | *proA* |
| *llo2749* |  | Similar to peptidase | *pepO* |
| *llo2786* |  | Component of the Dot/Icm secretion system | *icmK/dotH* |
| *llo2789* |  | Component of the Dot/Icm secretion system. Lipoprotein of the OmpA protein family | *IcmN/DotK* |
| *llo2839* |  | 50S ribosomal subunit protein L19 | *rplS* |
| *llo2841* |  | Similar to 16S rRNA processing protein RimM | *rimM* |
| *llo2842* |  | Highly similar to 30S ribosomal protein S16 | *rpsP* |
| *llo2855* |  | 30S ribosomal subunit protein S21 | *rpsU* |
| *llo2863* |  | Hypothetical protein | *_* |
| *llo3025* | specific | Putative citrate lyase beta subunit | *_* |
| *llo3036* |  | Hypothetical protein, weakly similar to eukaryotic proteins | *_* |
| *llo3153* | specific | Putative glycosyl transferase group 1 | *_* |
| *llo3239* |  | Similar to outer membrane lipoprotein | *_* |
| *llo3261* |  | Similar to transketolase | *tkt* |
| *llo3264* |  | Similar to pyruvate kinase II PykA, glucose stimulated | *pykA* |
| *llo3284* |  | Similar to GTP-binding protein | *yihA* |
| *llo3331* |  | Putative secreted protein | *_* |
| *llo3332* |  | Similar to unknown proteins | *_* |
| *llo3368* |  | Hypothetical protein | *_* |
| *llo3370* |  | Hypothetical protein | *_* |
| *llo3384* | specific | Hypothetical protein | *_* |
| *llo3418* |  | Highly Similar to H+-transporting ATP synthase epsilon chain | *atpC* |
| *llo3419* |  | Highly Similar to H+-transporting ATP synthase beta chain | *atpD* |
| *llo3420* |  | Highly Similar to H+-transporting ATP synthase chain gamma | *atpG* |
| *llo3421* |  | Highly Similar to H+-transporting ATP synthase chain alpha | *atpA* |
| *llo3422* |  | Highly Similar to H+-transporting ATP synthase chain delta | *atpH* |
| *llo3423* |  | Highly Similar to H+-transporting ATP synthase chain b | *atpF* |
| *llo3424* |  | Highly Similar to H+-transporting ATP synthase chain c | *atpE* |
| *llo3449* |  | Similar to conserved hypothetical protein | *_* |
| *llo3450* |  | Similar to ribonuclease P protein component (RNase P) | *rnpA* |
| *llo3451* |  | 50S ribosomal subunit protein L34 | *rpmH* |
