# Supplementary material for: Analysis of the Legionella longbeachae Genome and Transcriptome Uncovers Unique Strategies to Cause Legionnaires' Disease
Source: PLoS Genet. 2010 Feb 19;6(2):e1000851. doi: 10.1371/journal.pgen.1000851 (PMC2824747; doi:10.1371/journal.pgen.1000851)
Supplement: Table S8 — Genes upregulated in L. longbeachae in post-exponetial growth phase. (0.35 MB DOC) [file pgen.1000851.s014.doc]

**Table S8 :** **Genes upregulated in *L. longbeachae* in post-exponetial growth phase**

| **Gene** | **NSW150 specific** | | **Predicted fucntion** | **Gene name** |
| --- | --- | --- | --- | --- |
| *llo0022* |  | | Putative cation transport ATPase | *_* |
| *llo0040* | specific | | Hypothetical protein | *_* |
| *llo0041* | specific | | Hypothetical protein | *_* |
| *llo0053* |  | | Similar to amino acid permease | *_* |
| *llo0069* |  | | Putative transmembrane protein, Similar to Hypothetical membrane-bound serine protease | *nfeD* |
| *llo0070* |  | | Similar to protease | *_* |
| *llo0078* | specific | | Hypothetical protein | *_* |
| *llo0090* |  | | Putative regulatory protein (GGDEF, PAS, PAC domains) | *_* |
| *llo0091* |  | | Similar to conserved Hypothetical protein | *_* |
| *llo0105* | specific | | Putative membrane protein | *_* |
| *llo0115* |  | | Ankyrin repeat protein | *_* |
| *llo0120* | specific | | Putative capsule biosynthesis protein | *_* |
| *llo0200* |  | | Hypothetical protein | *_* |
| *llo0203* |  | | Putative lipase | *_* |
| *llo0241* |  | | MFS superfamily drug efflux transporter | *_* |
| *llo0265* |  | | Glycine-rich protein | *_* |
| *llo0310* |  | | Fused tRNA nucleotidyl transferase |  |
| *llo0315* | specific | | Putative V-type ATP synthase, subunit b | *atpB* |
| *llo0316* | specific | | Putative V-type ATP synthase, subunit d | *_* |
| *llo0319* | specific | | Putative V-type ATP synthase, subunit e | *_* |
| *llo0320* | specific | | Putative V-type ATP synthase, subunit a | *_* |
| *llo0383* | specific | | Putative UDP-glucose/GDP-mannose dehydrogenase | *capL* |
| *llo0424* |  | | Homologous to SidE substrate of Dot/Icm secretion system | *_* |
| *llo0426* |  | | Homologous to SidE substrate of Dot/Icm secretion system | *_* |
| *llo0429* |  | | Putative NAD-dependent malic enzyme | *sfcA* |
| *llo0446* | specific | | Hypothetical protein | *_* |
| *llo0451* |  | | Putative glutathione S-transferase | *yqjG* |
| *llo0452* | specific | | Putative aconitate hydratase | *_* |
| *llo0453* |  | | Putative phosphoribosyltransferase | *_* |
| *llo0455* | specific | | Hypothetical protein | *_* |
| *llo0456* |  | | Hypothetical protein | *_* |
| *llo0512* | specific | | Putative transposase (truncated) | *_* |
| *llo0552* |  | | Hypothetical protein | *_* |
| *llo0554* |  | | Highly Similar to *C. burnetii* heat shock protein HtpX | *_* |
| *llo0562* |  | | Putative membrane-associated metalloprotease proteins | *_* |
| *llo0569* |  | | Similar to type II secretion system protein-like protein and twitching mobility protein | *uptC* |
| *llo0608* | specific | | Hypothetical protein | *_* |
| *llo0619* |  | | Putative acetoacetate decarboxylase | *_* |
| *llo0621* |  | | Conserved exported protein of unknown function | *sdhC-2* |
| *llo0625* | specific | | Hypothetical protein | *_* |
| *llo0635* |  | | Hypothetical Protein | *_* |
| *llo0641* | specific | | Weakly similar to eukaryotic proteins | *_* |
| *llo0642* | specific | | Weakly similar to eukaryotic proteins | *_* |
| *llo0643* | specific | | Conserved Hypothetical protein | *_* |
| *llo0644* | specific | | Hypothetical protein | *_* |
| *llo0647* | specific | | Putative cytidine/deoxycytidylate deaminase | *_* |
| *llo0655* | specific | | Conserved Hypothetical protein | *_* |
| *llo0659* |  | | Putative NADP-dependent malic enzyme | *_* |
| *llo0661* |  | | Succinate dehydrogenase, FeS subunit | *sdhD-2* |
| *llo0662* |  | | Succinate dehydrogenase, membrane subunit, binds cytochrome b556 | *sdhA-2* |
| *llo0663* |  | | Succinate dehydrogenase, membrane subunit, binds cytochrome b556 | *rpoE* |
| *llo0664* |  | | Succinate dehydrogenase, flavoprotein subunit | *_* |
| *llo0665* |  | | Putative DNA-3-methyladenine glycosylase | *_* |
| *llo0666* |  | | Conserved Hypothetical protein | *_* |
| *llo0667* |  | | Aconitate hydratase (AcnA) homolog | *acnA* |
| *llo0690* |  | | Putative sensor histidine kinase | *_* |
| *llo0723* |  | | putative copper efflux ATPase | *_* |
| *llo0724* | specific | | Hypothetical integral membrane protein | *_* |
| *llo0726* | specific | | Putative D-isomer specific 2-hydroxyacid dehydrogenase | *_* |
| *llo0728* | specific | | Sulfate permease and related transporters | *_* |
| *llo0730* | specific | | Putative erythromycin esterase | *_* |
| *llo0743* |  | | Hypothetical protein | *_* |
| *llo0744* | specific | | Putative nitroreductase | *_* |
| *llo0747* | specific | | Putative muramoyl-tetrapeptide carboxypeptidase | *_* |
| *llo0748* | specific | | Putative coiled-coil protein | *_* |
| *llo0766* | specific | | Hypothetical protein | *_* |
| *llo0778* | specific | | Putative helicase, DEAD/DEAH box family | *_* |
| *llo0779* |  | | Putative pyoverdine biosynthesis regulatory protein SyrP-like | *_* |
| *llo0780* |  | | Hypothetical protein | *_* |
| *llo0794* |  | | Similar to C-terminal part of conserved Hypothetical protein | *_* |
| *llo0798* |  | | Putative coiled-coil protein | *_* |
| *llo0831* |  | | Hypothetical protein | *_* |
| *llo0852* |  | | Enhanced entry protein EnhA homolog | *_* |
| *llo0900* | specific | | Hypothetical protein | *_* |
| *llo0930* |  | | Putative multicopper oxidase | *cueO* |
| *llo0941* | specific | | Hypothetical protein | *_* |
| *llo0942* | specific | | Hypothetical protein | *_* |
| *llo0943* | specific | | Putative prophage maintenance system killer protein | *_* |
| *llo0944* | specific | | Hypothetical protein | *_* |
| *llo0948* |  | | Hypothetical protein | *_* |
| *llo0954a* | | | Hypothetical protein (N-terminal part) | *_* |
| *llo0960* | |  | Putative cation transporter/ATPase | *pacL* |
| *llo0964* | | specific | Hypothetical protein | *_* |
| *llo0967* | | specific | Hypothetical protein | *_* |
| *llo0992* | | specific | Hypothetical protein | *_* |
| *llo0993* | | specific | Hypothetical protein | *_* |
| *llo1001* | |  | Hypothetical protein | *yjgR* |
| *llo1004* | |  | Hypothetical protein | *_* |
| *llo1011* | |  | Hypothetical protein (fragment) | *_* |
| *llo1025* | |  | Hypothetical protein | *_* |
| *llo1042* | |  | Some Similarity with eukaryotic proteins | *_* |
| *llo1126* | |  | Hypothetical protein | *_* |
| *llo1135* | |  | Similar to transcriptional regulator, LysR family | *_* |
| *llo1137* | | specific | Hypothetical protein (cysteine rich repeat) | *_* |
| *llo1138* | | specific | Hypothetical protein | *_* |
| *llo1139* | | specific | Putative serine/threonine protein kinase | *_* |
| *llo1140* | |  | Putative glyoxalase/bleomycin resistance protein | *_* |
| *llo1169* | | specific | Hypothetical protein, weakly similar to eukaryotic proteins | *_* |
| *llo1180* | |  | Hypothetical protein | *_* |
| *llo1184* | | specific | Hypothetical protein | *_* |
| *llo1197* | | specific | Putative adenylate/guanylate cyclase | *_* |
| *llo1198* | | specific | Putative coiled-coil protein | *_* |
| *llo1225* | |  | Hypothetical protein | *_* |
| *llo1244* | | specific | Putative integral membrane protein | *_* |
| *llo1253* | |  | Regulatory protein (GGDEF domain) | *_* |
| *llo1254* | |  | Two-component sensor histidine kinase | *_* |
| *llo1255* | |  | Hypothetical protein | *_* |
| *llo1268* | | specific | Hypothetical protein | *_* |
| *llo1269* | | specific | Putative coiled-coil protein | *_* |
| *llo1272* | |  | Hypothetical protein | *_* |
| *llo1290* | | specific | Hypothetical protein | *_* |
| *llo1333* | |  | Putative alpha/beta hydrolase fold protein | *_* |
| *llo1336* | | specific | Hypothetical protein (cNMP_binding domain) | *_* |
| *llo1369* | | specific | Hypothetical protein, weakly similar to eukaryotic proteins | *_* |
| *llo1377* | |  | Putative sensory box/GGDEF domain protein | *_* |
| *llo1396* | | specific | Putative glutamine amidotransferase | *_* |
| *llo1409* | | specific | Putative mechanosensitive ion channel protein | *_* |
| *llo1435* | | specific | Putative secreted protein | *_* |
| *llo1473* | | specific | Hypothetical protein, weakly similar to eukaryotic proteins | *_* |
| *llo1474* | |  | Hypothetical protein | *_* |
| *llo1475* | |  | Some Similarity with EnhA protein | *_* |
| *llo1492* | |  | Similar to major facilitator membrane proteins | *phtH* |
| *llo1504* | |  | RNA polymerase, sigma 24 (sigma E) factor | *pilE* |
| *llo1539* | |  | Putative ABC transporter | *_* |
| *llo1541* | |  | Putative alpha/beta superfamily hydrolase | *_* |
| *llo1543* | | specific | Hypothetical protein | *_* |
| *llo1544* | | specific | Hypothetical protein | *_* |
| *llo1558* | | specific | Hypothetical protein, Similar to *P.luminescens* insecticidal toxin complex protein TcaZ | *_* |
| *llo1570* | |  | Similar to alcohol dehydrogenase | *_* |
| *llo1582* | |  | Hypothetical protein | *_* |
| *llo1604* | | specific | Hypothetical protein | *_* |
| *llo1608* | | specific | Putative methyltransferase | *_* |
| *llo1621* | | specific | Hypothetical protein | *_* |
| *llo1622* | | specific | Hypothetical protein | *_* |
| *llo1624* | | specific | Hypothetical protein | *_* |
| *llo1626* | | specific | Hypothetical protein | *_* |
| *llo1627* | | specific | Hypothetical protein | *_* |
| *llo1628* | | specific | Hypothetical protein | *_* |
| *llo1631* | | specific | Putative coiled coil protein, weakly similar to eukaryotic protein | *_* |
| *llo1641* | | specific | Hypothetical protein, weakly similar to eukaryotic proteins | *_* |
| *llo1646* | | specific | Ankyrin repeat protein | *_* |
| *llo1654* | |  | Hypothetical protein | *_* |
| *llo1655* | |  | Similar to other protein | *_* |
| *llo1656* | | specific | Hypothetical protein | *_* |
| *llo1659* | | specific | Hypothetical protein | *_* |
| *llo1669* | |  | Putative adenylate/guanilate cyclase | *_* |
| *llo1715* | | specific | Ankyrin repeat protein | *_* |
| *llo1737* | | specific | Hypothetical protein | *_* |
| *llo1825* | | specific | Putative methyltransferase | *_* |
| *llo1828* | | specific | Hypothetical protein, weakly similar to eukaryotic proteins | *_* |
| *llo1829* | | specific | Hypothetical protein | *_* |
| *llo1854* | | specific | Hypothetical protein | *_* |
| *llo1855* | | specific | Hypothetical protein | *_* |
| *llo1856* | | specific | Hypothetical protein | *_* |
| *llo1857* | | specific | Putative hydrolase | *_* |
| *llo1858* | | specific | Hypothetical protein | *_* |
| *llo1863* | |  | Putative SOS (Error prone) mutagenesis protein UmuD | *_* |
| *llo1864* | |  | Hypothetical protein | *_* |
| *llo1866* | | specific | Hypothetical protein | *_* |
| *llo1870* | | specific | Putative membrane protein | *_* |
| *llo1898* | | specific | Hypothetical protein | *_* |
| *llo1907* | | specific | Hypothetical protein | *_* |
| *llo1908* | |  | Hypothetical protein | *_* |
| *llo1956* | |  | Putative transcriptional regulator, LysR family | *_* |
| *llo1975* | | specific | Putative oxidoreductase | *zupT* |
| *llo1984* | | specific | Putative protein kinase | *_* |
| *llo1987* | |  | Similar to UmuD protein | *_* |
| *llo1988* | |  | Similar to conserved Hypothetical protein | *_* |
| *llo2005* | | specific | Putative regulatory protein (GGDEF domain) | *_* |
| *llo2058* | |  | Hypothetical protein | *_* |
| *llo2060* | | specific | Hypothetical protein | *_* |
| *llo2062* | | specific | Hypothetical protein | *_* |
| *llo2066* | | specific | Hypothetical protein | *_* |
| *llo2080* | |  | Similar to poly(3-hydroxyalkanoate) synthetase | *phbC* |
| *llo2119* | |  | Similar to cytochrome d ubiquinol oxidase subunit II | *qxtB* |
| *llo2120* | |  | Similar to cytochrome d ubiquinol oxidase subunit I | *qxtA* |
| *llo2141* | |  | Putative cAMP/cGMP binding protein | *_* |
| *llo2150* | |  | Putative ABC transporter, ATP-binding protein | *_* |
| *llo2152* | | specific | Hypothetical protein | *_* |
| *llo2165* | | specific | Hypothetical protein, LRR and tropomodulin domains | *_* |
| *llo2170* | |  | Hypothetical protein | *_* |
| *llo2171* | |  | Putative carboxylate-amine ligase | *_* |
| *llo2172* | |  | Hypothetical protein | *_* |
| *llo2173* | | specific | Putative coiled-coil protein | *_* |
| *llo2178* | | specific | Hypothetical protein | *_* |
| *llo2179* | | specific | Hypothetical protein | *_* |
| *llo2180* | | specific | Putative coiled-coil protein | *_* |
| *llo2197* | |  | Putative metallo-beta-lactamase family protein | *_* |
| *llo2198* | |  | Putative thymidine/pyrimidine-nucleoside phosphorylase | *_* |
| *llo2199* | |  | Putative ribose-phosphate pyrophosphokinase | *_* |
| *llo2210* | |  | Hypothetical protein, some Similarities with *L. pneumophila* SidE protein | *_* |
| *llo2216* | |  | Putative response regulator | *_* |
| *llo2223* | |  | Putative non-ribosomal peptide synthetase | *_* |
| *llo2229* | | specific | Putative amidohydrolase | *_* |
| *llo2230* | | specific | Hypothetical protein | *_* |
| *llo2248* | | specific | Hypothetical protein | *_* |
| *llo2257* | | specific | Putative proline-rich exported protein | *_* |
| *llo2269* | | specific | Hypothetical protein | *_* |
| *llo2290* | |  | Putative fatty acid desaturase (N-terminal) | *_* |
| *llo2291* | | specific | Putative fatty acid desaturase | *_* |
| *llo2298* | | specific | Hypothetical protein | *_* |
| *llo2300* | | specific | Hypothetical protein | *_* |
| *llo2309* | |  | Putative iron-sulpher cluster proteins NifU | *_* |
| *llo2313* | | specific | Putative coiled-coil protein, similar to eukaryotic protein | *_* |
| *llo2326* | |  | Similar to leucyl/phenylalanyl-tRNA-protein transferase | *aat* |
| *llo2343* | |  | Similar to enhanced entry protein EnhA | *_* |
| *llo2355* | | specific | Putative glycoside hydrolase (family 45), Similar to eukaryotic endoglucanase | *_* |
| *llo2383* | |  | Similar to polyhydroxyalkanoic-acid-synthase | *phbC* |
| *llo2384* | | specific | Hypothetical protein | *_* |
| *llo2385* | | specific | Integral membrane protein | *_* |
| *llo2387* | |  | Putative cAMP/cGMP binding protein | *_* |
| *llo2388* | |  | Similar to potassium uptake protein | *_* |
| *llo2397* | |  | Putative membrane protein | *_* |
| *llo2399* | | specific | Hypothetical protein | *_* |
| *llo2401* | | specific | Hypothetical protein | *_* |
| *llo2439* | |  | Hypothetical protein, Similar to SdhB (putative substrate of the Dot/Icm system) | *_* |
| *llo2456* | | specific | Putative coiled-coil protein | *_* |
| *llo2457* | | specific | Hypothetical protein | *_* |
| *llo2458* | | specific | Hypothetical protein | *_* |
| *llo2465* | |  | Hypothetical protein | *_* |
| *llo2466* | |  | Hypothetical protein | *_* |
| *llo2468* | | specific | Putative potassium-transporting ATPase, A subunit | *kdpA* |
| *llo2469* | | specific | Potassium-transporting ATPase B chain | *kdpB* |
| *llo2470* | | specific | Putative potassium-transporting ATPase B chain | *kdpC* |
| *llo2471* | | specific | Putative osmosensitive K+ channel His-kinase sensor | *_* |
| *llo2484* | | specific | Hypothetical protein | *_* |
| *llo2508* | |  | Hypothetical protein | *_* |
| *llo2509a* | | specific | Putative transposase (fragment) | *_* |
| *llo2522* | | specific | Hypothetical protein, weakly similar to eukaryotic proteins | *_* |
| *llo2525* | |  | Hypothetical protein | *_* |
| *llo2534* | |  | Conserved exported protein of unknown function | *_* |
| *llo2537* | | specific | Putative cyanophycin synthetase | *cphA* |
| *llo2538* | | specific | Hypothetical protein (PRC-barrel domain) | *_* |
| *llo2563* | |  | Putative ferritin, Dps family protein | *_* |
| *llo2581* | |  | Type-IV pilin | *_* |
| *llo2588* | |  | Similar to *E. coli* Ada protein (O6-methylguanine-DNA methyltransferase) | *_* |
| *llo2595* | | specific | Hypothetical protein | *_* |
| *llo2598* | | specific | Hypothetical protein | *_* |
| *llo2618* | |  | Putative serine transporter | *sdaC* |
| *llo2631* | | specific | Putative pyruvate phosphate dikinase | *_* |
| *llo2682* | | specific | Hypothetical protein | *_* |
| *llo2770* | |  | Putative alkyl hydroperoxide reductase D, carboxymuconolactonedecarboxylase | *_* |
| *llo2771* | |  | Putative alkyl hydroperoxide reductase/ thiol specific antioxidant | *ahpC* |
| *llo2826* | | specific | Putative pyoverdine biosynthesis protein PvcA | *_* |
| *llo2827* | |  | Hypothetical protein | *_* |
| *llo2836* | | specific | Hypothetical protein | *_* |
| *llo2837* | | specific | Hypothetical protein | *_* |
| *llo2850* | |  | Hypothetical protein | *_* |
| *llo2868* | | specific | Hypothetical protein | *_* |
| *llo2897* | | specific | Hypothetical protein | *_* |
| *llo2920* | |  | Putative aconitate hydratase | *acnA* |
| *llo2925* | | specific | Hypothetical protein | *_* |
| *llo2927* | |  | Putative universal stress protein family | *_* |
| *llo2935* | | specific | Hypothetical protein | *_* |
| *llo2936* | |  | Hypothetical protein | *_* |
| *llo2938* | |  | Hypothetical protein | *_* |
| *llo2940* | | specific | Hypothetical protein | *_* |
| *llo2951* | | specific | Hypothetical protein | *_* |
| *llo2954* | |  | Putative universal stress family protein | *_* |
| *llo2966* | | specific | Hypothetical protein (fragment) | *_* |
| *llo2971* | | specific | Putative ABC transporter ATP-binding protein | *_* |
| *llo2973* | | specific | Putative transmembrane protein | *_* |
| *llo2974* | |  | Putative small heat shock protein | *_* |
| *llo2976* | |  | Putative cation transport ATPase | *_* |
| *llo2977* | | specific | Putative membrane protein | *_* |
| *llo2983* | |  | Hypothetical protein | *_* |
| *llo2985* | | specific | Hypothetical protein | *_* |
| *llo2994* | | specific | Similar to eukaryotic glutamate decarboxylase | *gadB* |
| *llo2995* | | specific | Multiple antibiotic resistance (MarC)-related protein | *_* |
| *llo2996* | | specific | Hypothetical protein | *_* |
| *llo3001* | | specific | Hypothetical protein | *_* |
| *llo3032* | |  | Similar to eukaryotic cytokinin oxidase | *_* |
| *llo3033* | |  | Similar to unknown proteins | *_* |
| *llo3042* | | specific | Hypothetical protein | *_* |
| *llo3057* | |  | Protein with TPR motifs (protein-protein interaction motif) | *_* |
| *llo3060* | |  | Putative sodium/hydrogen exchanger | *ybaL* |
| *llo3092* | |  | Homologous to SidE substrate of Dot/Icm secretion system | *_* |
| *llo3095* | |  | Similar to Sid proteins | *_* |
| *llo3098* | |  | Similar to Sid proteins | *sidC* |
| *llo3109* | | specific | Fragment of Putative heat shock protein (N-terminal part)(part 1) | *_* |
| *llo3110* | |  | Putative ATP-dependent protease La, Lon superfamily | *_* |
| *llo3117* | | specific | Putative coiled-coil protein | *_* |
| *llo3119* | | specific | Putative coiled-coil protein | *_* |
| *llo3125* | |  | regulatory protein (GGDEF and EAL domains) | *_* |
| *llo3132* | |  | Putative prolyl oligopeptidase | *_* |
| *llo3135* | | specific | Hypothetical protein | *_* |
| *llo3136* | | specific | Hypothetical protein | *_* |
| *llo3137* | | specific | Hypothetical protein | *_* |
| *llo3140* | | specific | Putative aldehyde dehydrogenase family protein | *_* |
| *llo3217* | |  | Putative tRNA nucleotidyltransferase | *cca* |
| *llo3228* | |  | Putative citrate transporter | *_* |
| *llo3242* | | specific | Hypothetical protein | *_* |
| *llo3243* | | specific | Hypothetical protein | *_* |
| *llo3317* | |  | Putative two-component response regulator | *_* |
| *llo3321* | |  | Hypothetical protein | *_* |
| *llo3335* | |  | Hypothetical protein | *_* |
| *llo3342* | |  | Putative homospermidine synthase | *hss* |
| *llo3387* | |  | Putative dioxygenase, ferredoxin subunit | *_* |
| *llo3392* | |  | Putative regulatory protein (GGDEF and EAL domains) | *_* |
| *llo3395* | |  | Putative cyclic nucleotide-binding domain | *_* |
| *llo3396* | | specific | Hypothetical membrane protein | *_* |
| *llo3397* | | specific | Hypothetical protein | *_* |
| *llo3409* | | specific | Hypothetical protein | *_* |
| *llo3414* | |  | Putative regulatory protein (GGDEF domain) | *_* |
| *llo3429* | |  | Hypothetical protein | *_* |
| *llo3441* | | specific | Putative transmembrane protein | *_* |
| *llo3442* | |  | Putative sensory box histidine kinase/response regulator | *_* |
| *llo3443* | |  | Putative polyketide synthase | *_* |
| *llo3445* | | specific | Hypothetical protein, weak Similarity to eukaryotic proteins | *_* |
| *llo3446* | | specific | Hypothetical protein | *_* |
| *plllo0067* | |  | Hypothetical protein | *_* |
| *pllo0048* | |  | Enhanced entry protein EnhA homolog | *_* |
| *pllo0066* | |  | Peptidase S24 family protein | *_* |
