# Supplementary material for: Analysis of the Legionella longbeachae Genome and Transcriptome Uncovers Unique Strategies to Cause Legionnaires' Disease
Source: PLoS Genet. 2010 Feb 19;6(2):e1000851. doi: 10.1371/journal.pgen.1000851 (PMC2824747; doi:10.1371/journal.pgen.1000851)
Supplement: Table S9 — Sequence of primers used to amplify putative flagella gene encoding regions. (0.04 MB DOC) [file pgen.1000851.s015.doc]

**Table S9: Sequence of primers used to amplify putative flagella gene encoding regions**

| **Primer** | ***L. longbeachae*** | **Primer** | **control on *L.pneumophila* strain Paris** |
| --- | --- | --- | --- |
| LlofleR-fleS1.for | GTCCAGCTGCACAAAAGTTG | LppfleR-fleS1.for | CCAGTACCACCCTGTTTCCA |
| LLofleR-fleS1.rev | GGACTCCTGGCTGTATTTGC | LppfleR-fleS1.rev | TGACTTTATCAGCGCAATCG |
| llofleR-fleS2.for | ACGGCGTATTTTGAGAATGG | LpfleR-fleS2.for | CAGATGTCACCTCTCGCAAT |
| LlofleR-fleS2.rev | TTCTGATTTGATGGGCAAGC | LpfleR-fleS2.rev | GCGAAGACAAAGGGTACGAG |
| LlofliA-fliN1.for | ATAGCGAATCATTGCGAACC | LpfliA-fliN1.for | TGAACCGCACAGGAATGTTA |
| LlofliA-fliN1.rev | GGAGGAGAAAGCTGGGTCAT | LpfliA-fliN1.rev | CAATGTCGATGATGGTTTGC |
| LlofliA-fliN2.for | TGAGCTTGGTGTATCGCTTG | LpfliA-fliN2.for | CCTAAATCGGCATCCAGAAG |
| LlofliA-fliN2.rev | CTGCTCACCCCAACGTAAAT | LpfliA-fliN2.rev | GTCGAAACTTTGGCGGAATA |
| LloflgD.for | ATGCATTGAAACCCTCGAAC | LpflgD.for | TGTGGTTGCAGAGGATCTTG |
| LloflgD2.rev | GCCTGCAATCCTCTTCAAAC | LpflgD2.rev | GATACCCCTAATGCGCTGAA |
| LloflgD1.rev | AAGCACGCAACATGCTAAGA | |  |
